# Ongoing global and regional adaptive evolution of SARS-CoV-2

**DOI:** 10.1073/pnas.2104241118

**Published:** 2021-07-02

**Authors:** Nash D. Rochman, Yuri I. Wolf, Guilhem Faure, Pascal Mutz, Feng Zhang, Eugene V. Koonin

**Affiliations:** ^a^National Center for Biotechnology Information, National Library of Medicine, Bethesda, MD 20894;; ^b^Broad Institute of MIT and Harvard, Cambridge, MA 02142;; ^c^HHMI, Massachusetts Institute of Technology, Cambridge, MA 02139;; ^d^McGovern Institute for Brain Research, Massachusetts Institute of Technology, Cambridge, MA 02139;; ^e^Department of Brain and Cognitive Sciences, Massachusetts Institute of Technology, Cambridge, MA 02139;; ^f^Department of Biological Engineering, Massachusetts Institute of Technology, Cambridge, MA 02139

**Keywords:** SARS-Cov-2, phylogeny, ancestral reconstruction, epistasis, globalization

## Abstract

Understanding the trends in severe acute respiratory syndrome coronavirus 2 (SARS-CoV-2) evolution is paramount to control the COVID-19 pandemic. We analyzed more than 300,000 high-quality genome sequences of SARS-CoV-2 variants available as of January 2021. The results show that the ongoing evolution of SARS-CoV-2 during the pandemic is characterized primarily by purifying selection, but a small set of sites appear to evolve under positive selection. The receptor-binding domain of the spike protein and the region of the nucleocapsid protein associated with nuclear localization signals (NLS) are enriched with positively selected amino acid replacements. These replacements form a strongly connected network of apparent epistatic interactions and are signatures of major partitions in the SARS-CoV-2 phylogeny. Virus diversity within each geographic region has been steadily growing for the entirety of the pandemic, but analysis of the phylogenetic distances between pairs of regions reveals four distinct periods based on global partitioning of the tree and the emergence of key mutations. The initial period of rapid diversification into region-specific phylogenies that ended in February 2020 was followed by a major extinction event and global homogenization concomitant with the spread of D614G in the spike protein, ending in March 2020. The NLS-associated variants across multiple partitions rose to global prominence in March to July, during a period of stasis in terms of interregional diversity. Finally, beginning in July 2020, multiple mutations, some of which have since been demonstrated to enable antibody evasion, began to emerge associated with ongoing regional diversification, which might be indicative of speciation.

## Significance

Understanding the ongoing evolution of SARS-CoV-2 is essential to control and ultimately end the pandemic. We analyzed more than 300,000 SARS-CoV-2 genomes available as of January 2021 and demonstrate adaptive evolution of the virus that affects, primarily, multiple sites in the spike and nucleocapsid protein. Selection appears to act on combinations of mutations in these and other SARS-CoV-2 genes. Evolution of the virus is accompanied by ongoing adaptive diversification within and between geographic regions. This diversification could substantially prolong the pandemic and the vaccination campaign, in which variant-specific vaccines are likely to be required.

High mutation rates of RNA viruses enable adaptation to hosts at a staggering pace (1–4). Nevertheless, robust sequence conservation indicates that purifying selection is the principal force shaping the evolution of virus populations, with positive selection affecting only relatively small subsets of sites directly involved in virus−host coevolution (5–8). The fate of a novel zoonotic virus is, in part, determined by the race between public health intervention and virus diversification. Even intermittent periods of positive selection can result in lasting immune evasion, leading to oscillations in the size of the susceptible population and, ultimately, a regular pattern of repeating epidemics, as has been amply demonstrated for influenza (9–11).

During the current coronavirus pandemic (COVID-19), understanding the degree and dynamics of the diversification of severe acute respiratory syndrome coronavirus 2 (SARS-Cov-2) and identification of sites subject to positive selection are essential for establishing practicable, proportionate public health responses, from guidelines on isolation and quarantine to vaccination (12). To investigate the evolution of SARS-CoV-2, we collected all available SARS-Cov-2 genomes as of January 8, 2021, and constructed a global phylogenetic tree using a “divide and conquer” approach. Patterns of repeated mutations fixed along the tree were analyzed in order to identify the sites subject to positive selection. These sites form a network of potential epistatic interactions. Analysis of the putative adaptive mutations provides for the identification of signatures of evolutionary partitions of SARS-CoV-2. The dynamics of these partitions over the course of the pandemic reveals alternating periods of globalization and regional diversification.

## Results and Discussion

### Global Multiple Sequence Alignment of the SARS-CoV-2 Genomes.

To investigate the evolution of SARS-CoV-2, we analyzed all SARS-Cov-2 genomes, as of January 8, 2021, in the Gisaid (13) database, which included the vast majority of SARS-CoV-2 sequences available at that time. From the total of 321,096 submissions in Gisaid, 175,857 unique SARS-Cov-2 genome sequences were identified, and 98,090 high-quality sequences were incorporated into a global multisequence alignment (MSA) consisting of the concatenated open reading frames with stop codons trimmed. The vast majority of the sequences excluded from the MSA were removed due to a preponderance of ambiguous characters (see *Brief Methods*). The sequences in the final MSA correspond to 233,181 isolates with associated date and location metadata.

### Tree Construction.

Several methods for coronavirus phylogenetic tree inference have been tested (14, 15). The construction of a single high-quality tree from nearly 200,000 30-kilobase (kb) sequences using any of the existing advanced methods is computationally prohibitive. Iterative construction of the complete phylogeny would seem an obvious solution such that a global topology would be obtained based on a subset of sequences available at an earlier date, and later sequences would be incorporated into the existing tree. However, this approach induces artifacts through the inheritance of deep topologies that differ substantially from any maximum likelihood solution corresponding to the complete alignment.

Therefore, building on the available techniques, we utilized a “divide and conquer” approach which is not subject to these artifacts and furthermore can be employed for datasets that cannot be structured by sequencing date, including metagenomic analyses. This approach leverages two ideas. First, for any alignment, a diverse representative subset of sequences can be used to establish a deep topology, the tree “skeleton,” that corresponds to a maximum likelihood solution over the entire alignment. Second, deep branches in an unrooted tree are primarily determined by common substitutions relative to consensus. In other words, rare substitutions are unlikely to affect deep splits or branch lengths. We adopted the following steps to resolve the global phylogeny for SARS-CoV-2 (see *Brief Methods* for details).

The first step is to construct sets of diverse representative sequences such that the topologies inferred from each subset share the same tree “skeleton” corresponding to that of the global topology. Sequence diversity is measured by the hamming distance between pairs of sequences; however, maximizing hamming distances among a set of representative sequences does not guarantee maximization of the tree distances in the resultant global topology and so does not guarantee the maximum likelihood topology of this subset would share the global tree skeleton. Therefore, a reduced alignment containing only the top 5% of sites (ignoring nearly invariant sites; see below) with the most common substitutions relative to consensus was constructed. Sequences redundant over this narrow alignment were removed. Sets of diverse—based on the hamming distances over this reduced alignment—representative sequences were then achieved, and all subtrees generated from each diverse subset were aggregated to constrain a single, composite tree.

**Fig. 1.**
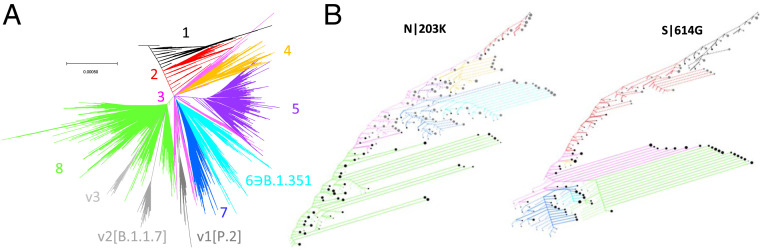
Global phylogeny of SARS-CoV-2. (*A*) Global tree reconstruction with eight principal partitions and three variant clades enumerated and color coded. (*B*) Site history trees for spike 614 and nucleocapsid 203 positions. Nodes were included in this reduced tree based on the following criteria: those immediately succeeding a substitution; those representing the last common ancestor of at least two substitutions; or terminal nodes representing branches of five sequences or more (approximately, based on tree weight). Edges are colored according to their position in the main partitions, and the line type corresponds to the target mutation (solid) or any other state (dashed). Synonymous mutations are not shown. These sites are largely binary, as are most sites in the genome. The terminal node sizes are proportional to the log of the weight descendent from that node beyond which no substitutions in the site occurred. Node color corresponds to target mutation (black) or any other state (gray).

This composite tree reflects the correct tree skeleton and could be used to constrain the global topology; however, due to numerous sequencing errors in this dataset, another intermediate step was taken. A second reduced alignment was constructed in which nearly invariant sites, which may represent sequencing errors and should not be used to infer tree topology, were omitted. As before, sequences redundant over this reduced alignment were removed, and a tree was then constructed from this alignment, constrained to maintain the topology of the composite tree wherever possible. This tree reflects the correct topology of the global tree but has incorrect branch lengths. Finally, the global tree with the correct branch lengths (Fig. 1*A*) was constructed over the whole alignment, constrained to maintain the topology of the previous tree wherever possible. A complete reconstruction of ancestral sequences was then performed by leveraging Fitch Traceback (16), enabling comprehensive identification of nucleotide and amino acid replacements across the tree (Fig. 1*B* shows the history of the ancestral states for the key sites in the N and S proteins, N|203 and S|614).

We identified eight principal partitions within this tree, in general agreement with other work (17–19), along with three divergent clades (Fig. 1*A*) that, as discussed below, are important for the interpretation of the metadata. In particular, v1 corresponds to variant of concern (VOC) P.1; v2 corresponds to VOC B.1.1.7, and VOC B.1.351 is contained within partition 6. Given the short evolutionary distances between SARS-CoV-2 isolates, despite the efforts described above, the topology of the global tree is a cause of legitimate concern (15, 20–22). For the analyses presented below, we rely on a single, explicit tree topology which is probably one of many equally likely estimates (15). Therefore, we sought to validate the robustness of the major partitions of the virus genomes, using a phylogeny-free approach. To this end, pairwise Hamming distances were computed for all sequences in the MSA, and the resulting distance matrix was embedded in a three-dimensional subspace using classical multidimensional scaling. In this embedding, the eight partitions are separated, and the optimal clustering, determined by *k* means, returned five categories (see *Brief Methods* and *SI Appendix*, Fig. S1), of which four correspond to partitions 5 and 8, and the divergent clades v1 and v2. These findings indicate that an alternative tree with a comparable likelihood but a dramatically different coarse-grain topology, most likely, cannot be constructed from this MSA.

### Mutational Signatures and Biases and Estimation of Selection.

**Fig. 2.**
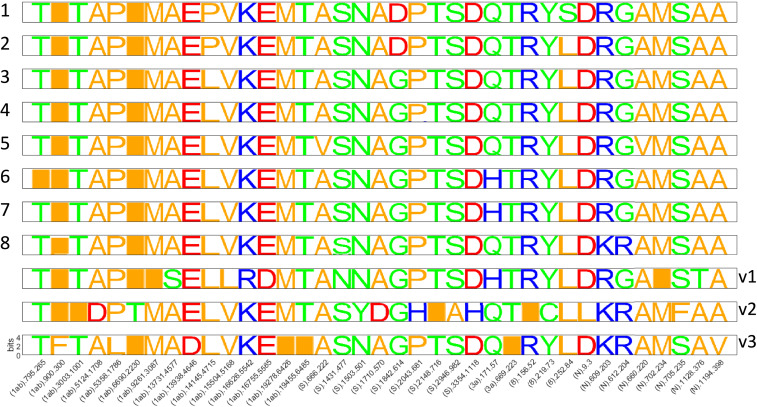
SARS-CoV-2 signature mutations. Signatures of amino acid replacements for each partition. Sites are ordered as they appear in the genome. The proteins, along with the nucleotide and amino acid numbers, are indicated underneath each column.

**Fig. 3.**
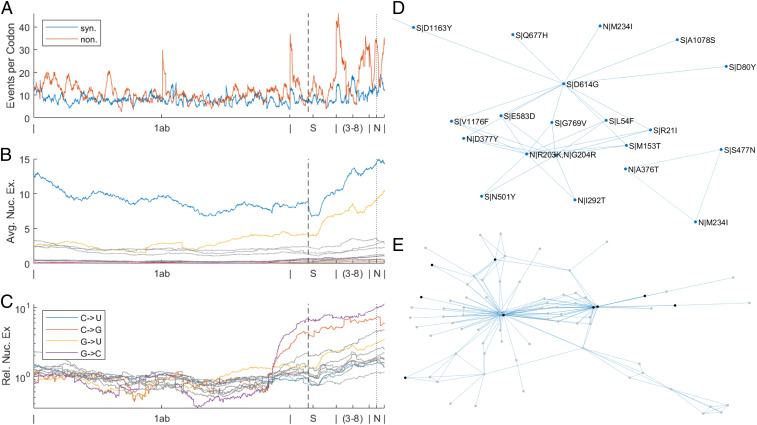
Global phylogeny of SARS-CoV-2. (*A*) Moving averages, respecting segment boundaries, across a 100-codon window for synonymous and nonsynonymous substitutions per site. There are several regions in the genome with an apparent dramatic excess of synonymous substitutions, including 5′ end of orf1ab gene, most of the M gene, and 3′ half of the N gene. There are also regions with substantially elevated rate of amino acid substitutions, including most of the orf3a gene, most of the orf7a gene, most of the orf8 gene, and several regions in the N gene. (*B*) Moving average over a window of 1,000 codons, not respecting segment boundaries, of the total number of nucleotide substitutions n1→n2 summed over all substitutions. (*C*) Moving average over a window of 1,000 codons, not respecting segment boundaries, of the total number of nucleotide substitutions n1→n2 summed over all substitutions (as in *B*) normalized by the median over all windows. (*D*) Network of putative epistatic interactions for likely positively selected residues in the N and S proteins. (*E*) Network of putative epistatic interactions for mutations meeting all other criteria for positive selection, regardless of the NCN context. Mutations in the polyprotein are not displayed. Black nodes correspond to key amino acid substitutions S|L18F, S|A222V, S|S477N, S|N501Y, S|D614G, S|P681H, S|T716I, N|R203K, and N|G204R. See *SI Appendix*, Fig. S10 for labeled graph.

Each of the eight partitions and three variant clades can be characterized by a specific amino acid replacement signature (Fig. 2), generally, corresponding to the most prominent amino acid replacements across the tree (*SI Appendix*, Table S1), some of which are shared by two or more partitions and appear independently many times, consistent with other reports (23). The receptor-binding domain (RBD) of the spike protein and a region of the nucleocapsid protein associated with nuclear localization signals (NLS) (24) are enriched with these signature replacements, but they are also found in the nonstructural proteins 1ab, 3a, and 8. The identification of these prevailing nonsynonymous substitutions and an additional set of frequent synonymous substitutions suggested that certain sites in the SARS-CoV-2 genome might be evolving under positive selection. However, uncovering the selective pressures affecting virus evolution was complicated by nonnegligible mutational biases. The distributions of the numbers of both synonymous and nonsynonymous substitutions across the genome were found to be substantially overdispersed compared to both the Poisson and normal expectations (Fig. 3*A* and *SI Appendix*, Fig. S2). Examination of the relative frequencies of all 12 possible nucleotide substitutions indicated a significant genome-wide excess of C to U mutations, approximately threefold higher than any other nucleotide substitution, with the exception of G to U, as well as some region-specific trends. Specifically, G to U mutations increase steadily in frequency throughout the second half of the genome, and the distribution of nucleotide substitutions over the polyprotein is dramatically different from other open reading frames (ORFs) (Fig. 3 *B* and *C* and *SI Appendix*, Fig. S3).

Motivated by the observation of the mutational biases, we compared the trinucleotide contexts of synonymous and nonsynonymous substitutions as well as the contexts of low- and high-frequency substitutions. The contexts of high-frequency events, both synonymous and nonsynonymous, were found to be dramatically different from the background frequencies. The NCN context (that is, all C→D mutations) harbors substantially more events than other contexts (all 16 NCN triplets are within the top 20 most high-frequency biased; see *Brief Methods* and *SI Appendix*, Fig. S4) and is enriched in mutations uniformly across the genome, primarily among high-frequency sites. This pattern suggests a mechanistic bias of the errors made by the coronavirus RNA-dependent RNA polymerase (RdRP). Evidently, such a bias that increases the likelihood of observing multiple, independent substitutions in the NCN context complicates the detection of selection pressures. However, only two of the nine contexts with an excess of nonsynonymous events are NCN (gct,tct; *SI Appendix*, Fig. S4), suggesting that at least some of these repeated, nonsynonymous mutations are driven by other mechanisms. Thus, we excluded all synonymous substitutions and nonsynonymous substitutions with the NCN context from further consideration in the determination of candidate sites evolving under positive selection.

Beyond this specific context, the presence of any hypervariable sites complicates the computation of the *dN/dS* ratio, the gauge of protein-level selection (25), which requires enumerating the number of synonymous and nonsynonymous substitutions within each gene. Hypervariable sites bias this analysis, and, therefore, we used two methods to ensure reliable estimation of *dN/dS*. For each protein-coding gene of SARS-CoV-2 (splitting the long orf1ab into 15 constituent nonstructural proteins), we obtained both a maximum likelihood estimate of *dN/dS* across 10 subalignments and an approximation computed from the global ancestral reconstruction (see *Brief Methods*). This approach was required due to the size of the alignment, which makes a global maximum likelihood estimation computationally prohibitive. The high sampling density and the low likelihood of undercounting mutations within the SARS-CoV-2 phylogeny provide for accurate approximations from the global ancestral reconstruction, which requires direct counting of mutations. Related methods that further develop this approach have been previously applied to the study of influenza virus evolution (26).

Despite considerable variability among the genes, we obtained estimates of substantial purifying selection (0.1< *dN/dS* < 0.5) across most of the genome (*SI Appendix*, Fig. S5), with a reasonable agreement between the two methods. This estimate is compatible with previous demonstrations of purifying selection affecting about 50% of the sites surveyed or more (5), among diverse RNA viruses (6). Direct enumeration of nucleotide substitutions in the SARS-CoV-2 phylogeny yields a number of nonsynonymous substitutions exceeding the number of synonymous substitutions (nN/nS > 1), which is not the typical picture for evolution under purifying selection (27). As discussed below, this could be largely due to ongoing, rapid host adaptation as well as the extremely high sampling density. The latter could result in the identification of many nonsynonymous mutations, which are transient polymorphisms and would be lost at a longer sampling interval, being purged by purifying selection. Furthermore, neither of these *dN/dS* estimates explicitly correct for the mutational biases, so that, if mutations within the NCN context were excluded, the *dN/dS* estimate across the genome might increase.

### Evidence of Positive Selection.

As shown in the previous section, evolution of SARS-CoV-2 is likely primarily driven by substantial purifying selection. However, more than 100 nonsynonymous substitutions appeared to have emerged multiple times, independently, covering a substantial portion of the tree equivalent to ∼200 or more terminal branches or “leaves,” and were not subject to an overt mechanistic bias. Due to the existence of many equally likely trees, in principle, in one or more such trees, any of these mutations could resolve to a single event. However, such a resolution would be at the cost of inducing multiple parallel substitutions for other mutations, and thus we conclude that more than 100 codons in the genome that are not subject to an overt mechanistic bias underwent multiple parallel mutations in the course of SARS-CoV-2 evolution during the COVID-19 pandemic.

One immediate explanation of this observation is that these sites evolve under positive selection. The possible alternatives could be that these sites are mutational hotspots or that the appearance of multiple parallel mutations was caused by numerous recombination events (either real or artifacts caused by incorrect genome assembly from mixed infections) in the respective genomic regions. Contrary to what one would expect under the hotspot scenario, we found that codons with many synonymous substitutions tend to harbor few nonsynonymous substitutions, and vice versa (*SI Appendix*, Fig. S6*A*). When a moving average with increasing window size was computed, only a weak positive correlation was observed between the numbers of synonymous and nonsynonymous substitutions (*SI Appendix*, Figs. S6 *B* and *C* and S7). Most sites in the virus genome are highly conserved, the sites with most substitutions tend to reside in conserved neighborhoods, and the local fraction of sites that harbor at least one mutation strongly correlates with the moving average (*SI Appendix*, Fig. S8). Together, these observations indicate that SARS-CoV-2 genomes are subject to diverse site-specific and regional selection pressures, but we did not detect regions of substantially elevated mutation or recombination, in general agreement with other studies (28), despite the role recombination might have played in zoonosis (29–34).

### Positively Selected Sites in SARS-CoV-2 Proteins.

Given the widespread purifying selection affecting evolving SARS-CoV-2 genomes, substantially relaxed selection at any site is expected to permit multiple, parallel, nonsynonymous mutations to the same degree that any site harbors multiple, parallel, synonymous mutations. Thus, seeking to identify sites subject to positive selection, we focused only on those nonsynonymous substitutions that independently occurred more frequently than 90% of all synonymous substitutions excluding the mutagenic NCN context (see *Brief Methods*). Most if not all sites in the SARS-CoV-2 genome that we found to harbor such frequent, parallel, nonsynonymous substitutions outside of the NCN context can be inferred to evolve under positive selection (*SI Appendix*, Table S2, List 1). The positively selected residues form a cooccurrence network that likely reflects epistatic interactions (Fig. 3*D* and *SI Appendix*, Table S3; see *Brief Methods*), in which the central hubs are D614G in the spike (S) protein and two adjacent substitutions in the nucleocapsid (N) protein, R203K and G204R, the three most common positively selected mutations (Figs. 1*B* and 3*D*) (35).

### Positively Selected Amino Acid Replacements in the RBD of the Spike Protein.

Spike D614G appears to boost the infectivity of the virus, possibly by increasing the binding affinity between the spike protein and the cell surface receptor of SARS-CoV-2, ACE2 (36). Conclusively demonstrating selection for a single site has proven challenging (37), even within this robust dataset. Although the emergence of this mutation corresponds to the extinction of partitions lacking 614G (see below), the possibility remains that this mutation is a passenger to some other mutagenic or epidemiological event. The 614 site of the S protein is evolutionarily labile, so that the ancestral reconstruction includes multiple gains of 614D after a previous loss. As a result, the reverse replacement G614D appears often enough to pass our statistical criteria for positive selection. Although severely biasing against recent events, one can additionally require that the mean tree fraction descendant from each candidate positively selected amino acid replacement be sufficiently large, removing from consideration events which are frequent but shallow (see *Brief Methods*). The addition of this criterion results in a “shortlist” of 22 residues subject to the strongest selection (*SI Appendix*, Table S2, List 2) that do not include 614D.

Additionally, apart from the selective advantage of a single replacement, it should be emphasized that D614G (but not G614D) is a central hub of the epistatic network (Fig. 3*D*). Conceivably, epistatic interactions with this residue can result in ensembles of mutations which substantially increase fitness. The ubiquitous epistasis throughout molecular evolution (38–41) suggests the possibility that many if not most mutations, which confer a substantial selective advantage, do so only within a broader epistatic context, not in isolation. By increasing the receptor affinity, D614G apparently opens up new adaptive routes for later steps in the viral lifecycle. The specific mechanisms of such hypothetical enhancement of virus reproduction remain to be investigated experimentally.

In addition to 614G, 31 spike mutations, most within the RBD, are signature mutations for divergent clades v1 to v3; emergent variants vAfrica or vOceania (see below); or established variants B.1.1.7, B.1.1.7_E484K, B.1.258_delta, B.1.351, B.1.429, P.1, or P.2 (42–47) (*SI Appendix*, Table S4, List 1). Most of the mutations in the VOCs were not phylogenetically informative, having emerged independently multiple times in more than one partition, and not all VOC sequences clustered within the tree. The notable exceptions are P.2, which corresponds to v1, and B.1.1.7, which corresponds to v2. Additionally, although the mutations that characterize B.1.351 do not form a clade, they are largely or exclusively represented within partition 6 and correspond to the emergent variant vAfrica defined below through trends in the metadata.

Three of these signature mutations pass the strict criteria for positive selection—S|N501Y, S|S477N, and S|V1176F—and S|N501Y makes the shortlist of the 22 strongest candidates. H69del/V70del are signature mutations for variant B.1.258_delta and have been previously observed to have rapidly emerged in an outbreak among minks (48, 49). A two−amino acid deletion (in our alignment, this deletion resolves to sites 68/69 due to many ambiguous characters in this neighborhood) appears multiple times independently throughout the tree and is present in approximately one-third of the European sequences from January 2021 (*SI Appendix*, Fig. S9; deletions are not shown in Fig. 2; see below).

Two sites within the RBD, N331 and N343, have been shown to be important for the maintenance of infectivity (50). As could be expected, these amino acid residues are invariant. Four more substitutions in the RBD, among others, N234Q, L452R, A475V, and V483A, have been demonstrated to confer antibody resistance (50). N234Q, A475V, and V483A were never or rarely found in our alignment, but L452R is a signature of variant B.1.429. Although not meeting our criteria for positive selection, it appeared multiple times across the tree, including within partition 1. Of greatest concern is perhaps N501Y. This amino acid replacement is a signature of variants B.1.1.7, B.1.1.7_E484K, and B.1.351, P.1; divergent clade v2; and emergent variant vAfrica. N501Y is among the 22 strongest candidates for positive selection and has been demonstrated to escape neutralizing antibodies (51). N501T in the same site is of additional concern (52) and has also been observed in mink populations (53). Additionally, S|N439K, a signature mutation for variant B.1.258_DELTA that has been demonstrated to enable immune escape (54), is observed in a large portion of the tree.

The emergence of multiple mutations associated with immune evasion during a period of the pandemic when the majority of the global population had remained naïve is striking. Such adaptations are generally expected to emerge among host populations where many individuals have acquired immunity either through prior exposure or vaccination (55–58). Furthermore, this pattern of, most likely independent, emergence of persisting variants among both human and mink populations suggests the possibility that these mutations represent nonspecific adaptations acquired shortly after zoonosis. The factors underpinning the evolution of viral life history traits after zoonosis, especially virulence, remain poorly understood (59) but apparently result from selective pressures imposed by both epidemiological parameters (host behavior) (60, 61), which may be conserved across a variety of novel hosts, and specific properties of the host receptor. Whereas emergent mutations in the RBD of SARS-CoV-2 are, for obvious reasons, surveyed with great intensity, we have to emphasize the enrichment of positively selected residues in the N protein, which might relate to more deeply taxonomically conserved routes of host adaptation for beta-coronaviruses.

### Amino Acid Replacements Associated with the Nuclear Localization Signals in the Nucleocapsid Protein.

Evolution of beta coronaviruses with high case fatality rates, including SARS-CoV-2, was accompanied by accumulation of positive charges in the N protein that might enhance its transport to the nucleus (62). Thirteen amino acid replacements in the N protein are signatures among the variants or major partitions discussed here, seven of which—203K, 204R, 205I, 206F, 220V, 234I, and 235F—are in the vicinity of the known NLS motifs or other regions responsible for nuclear shuttling (24). Two additional substitutions, 194L and 199L, rose to prominence in multiple regions during the summer of 2020. Two of these NLS-adjacent amino acid replacements, R(agg)203K(aaa) and G(gga)204R(cga), almost always appear together. This pair of substitutions includes the second and third most common positively selected sites after S614, and, although another adjacent site, S(agt)202N(aat) is not a signature mutation, it is the eighth most common positively selected residue. Among the 22 nonsynonymous substitutions that are apparently subject to the strongest selection (*SI Appendix*, Table S2, List 2), 6 are in the N protein (202N, 203K, 204R, 234I, 292T, and 376T).

The replacements R(agg)203K(aaa) and G(gga)204R(cga) occur via three adjacent nucleotide substitutions. R(agg)203K(aaa) resolves to two independent mutations in the ancestral reconstruction: first, R(agg)203K(aag), then K(aag)203K(aaa). Furthermore, the rapid rise of 220V (excluded from consideration as a candidate for positive selection in our analysis due to its NCN context) in a European cohort during the summer of 2020 might be related to a transmission advantage of the variant carrying this substitution (63). These substitutions, in particular, G(gga)204R(cga), which increases the positive charge, might contribute to the nuclear localization of the N protein as well. This highly unusual cluster of multiple signature and positively selected mutations across five adjacent residues in the N protein is a strong candidate for experimental study that could illuminate the evolution and perhaps the mechanisms of SARS-CoV-2 pathogenicity.

In addition to the many mutations of interest in the N and S proteins, Orf3a|Q57H is a signature mutation for partitions 6, 7, and v1. Q57H is the fourth most common positively selected mutation. Although not considered a candidate for positive selection in our analysis due to its NCN context, ORF8 S84L is a hub in the larger epistatic network including all strongly associated residues (Fig. 3*E* and *SI Appendix*, Fig. S10).

We also identified numerous nonsense mutations. Of particular interest seems to be ORF8|Q27*, which is a signature for variants B.1.1.7 and B.1.1.7_E484K and could be epistatically linked to positively selected residues including N|R203K and S|D614G. ORF8 has been implicated in the modulation of host immunity by SARS-CoV-2, so these truncations might play a role in immune evasion (64, 65).

### Potential Role of Epistasis in the Evolution of SARS-CoV-2.

Epistasis in RNA virus evolution, as demonstrated for influenza, can constrain the evolutionary landscape and promote compensatory variation in coupled sites, providing an adaptive advantage which would otherwise impose a prohibitive fitness cost (66–68). Because even sites subject to purifying selection can play an adaptive role through interactions with other residues in the epistatic network (69), the networks presented here Fig. 3 *D* and *E* (*SI Appendix*, Fig. S10) likely underrepresent the extent of epistatic interactions occurring during SARS-CoV-2 evolution. The early evolutionary events that shaped the epistatic network likely laid the foundation for the diversification of the virus relevant to virulence, immune evasion, and transmission. As discussed above, these early mutations (including S|G614D) might provide only a modest selective advantage in isolation but exert a much greater effect through multiple epistatic interactions.

The epistatic network will continue to evolve through the entirety of the pandemic, and, indeed, all emerging variants at the time of this writing are defined not by a single mutation but by an ensemble of signature mutations. Moreover, in addition to the apparent widespread intraprotein epistasis, there seem to exist multiple epistatic interactions between the N and S proteins. In particular, S|N501Y and N|S235F are both signature mutations for variants B.1.1.7 and B.1.1.7_E484K (*SI Appendix*, Table S4, List 2), and this pair is in the top 25% of cooccurring pairs in our network, ranked by lowest probability of random cooccurrence.

As with early founder mutations, when a new variant emerges with multiple signature mutations, it is unclear which, if any, confer a fitness advantage. Although it is natural to focus on substitutions within the RBD, we emphasize that all emergent variants contain substitutions in in the vicinity of known NLS motifs. In fact, the most statistically significant signature mutation (based on the Kullback−Leibler divergence) for vAfrica (consistent with variant B.1.351; see below) is N|T205I. As we suggest for S|D614G, these variant signature mutations are likely to exert a greater influence through multiple epistatic interactions than in isolation, and each signature mutation can be a member of multiple epistatic ensembles beyond the group of signature mutations within which it was originally identified. Indeed, signature mutations are shared among defined variants, and we find evidence for an additional 18 putative epistatic interactions between variant signature mutations and other events throughout the tree which are not identified as signature mutations for any defined variant (*SI Appendix*, Table S4, List 3). The growing ensemble of signature mutations that appear to be subject to positive selection, and the existence of a robust network of putative epistatic interactions including these signatures, suggest that ongoing virus diversification is driven by host adaptation rather than occurring simply by neutral drift.

### Epidemiological Trends and Ongoing Diversification of SARS-CoV-2.

Analysis of within-patient genetic diversity of SARS-CoV-2 has shown that the most common mutations are highly diverse within individuals (70–72). Such diversity could either result from multiple infections or, otherwise, could point to an even greater role of positive selection affecting a larger number of sites than inferred from our tree. Similarly to the case of influenza, positive selection on these sites could drive virus diversification and might support a regular pattern of repeat epidemics, with grave implications for public health. An analysis of the relationships between the sequencing date and location of each isolate and its position within the tree can determine whether diversification is already apparent within the evolutionary history of SARS-CoV-2.

**Fig. 4.**
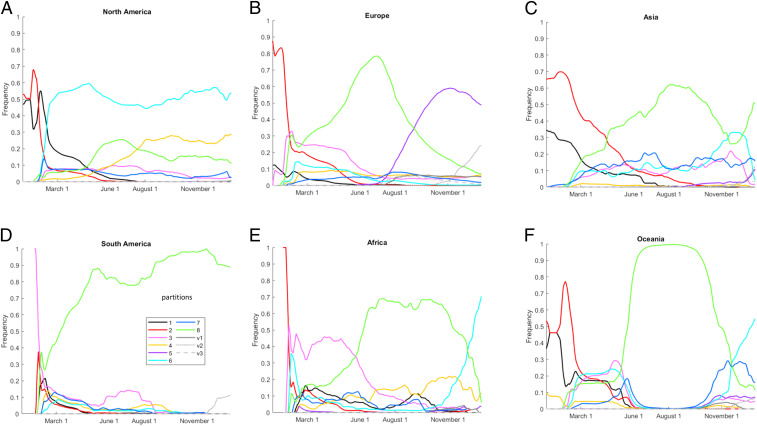
Regional SARS-CoV-2 partition dynamics during the COVID-19 pandemic: (*A*) North America, (*B*) Europe, (*C*) Asia, (*D*) South America, (*E*) Africa, and (*F*) Oceania. Probability distributions shown; for the absolute number of sequences, see *SI Appendix*, Fig. S15. Different colors within this figure denote regions and not partitions as they had in Figs. 1–3.

**Fig. 5.**
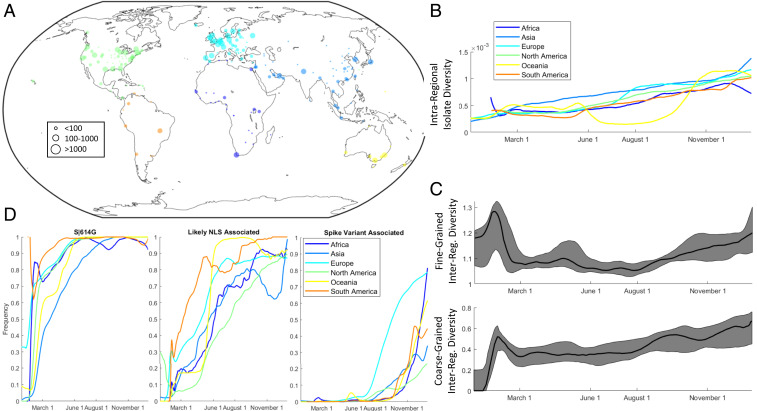
Global and regional trends in SARS-CoV-2 evolution. (*A*) Global distribution of sequences with sequencing locations in each of the six regions considered. Color scheme is for visual distinction only. (*B*) Intraregional diversity measured by the mean tree distance for pairs of isolates. (*C*) (*Top*) The Hellinger distance for all pairs of regions over the 11 partition/clade distribution; 25th, 50th, and 75th percentiles are shown. (*Bottom*) The ratio of the mean tree distance for pairs of isolates between regions vs. isolates within regions; 25th, 50th, and 75th percentiles are shown. (*D*) The frequency of S|614G, at least one NLS-associated variant (N|194L, N119L, N203K, N205I, and N220V), and at least one emerging spike variant (*SI Appendix*, Fig. S23, excluding S|477N). Different colors within this figure denote regions and not partitions.

We first demonstrated a strong correlation between the sequencing date of SARS-CoV-2 genomes and the distance to the tree root (*SI Appendix*, Fig. S11), indicating a sufficiently low level of noise in the data for subsequent analyses. Examination of the global distribution of each of the major SARS-CoV-2 partitions (*SI Appendix*, Figs. S12–S14) indicates dramatic regional differences and distinct temporal dynamics (Fig. 4). A measure of virus diversity is necessary to map to these trends. We considered two modes of diversity. Intraregional diversity reflects the mutational repertoire of the virus circulating in any individual region within any window of time. To measure intraregional diversity, we sampled pairs of isolates from each region and time point and computed the mean tree distance for a representative ensemble of these pairs. We found that intraregional diversity has been steadily increasing throughout the entirety of the pandemic, with the exception of Oceania from June to August 2020 (Fig. 5 *A* and *B*), which corresponds to the period following a bottleneck in the total number of infections (*SI Appendix*, Fig. S15) within that region. This unabated intraregional diversification is further evidence of a large repertoire of host-adaptive mutations of SARS-CoV-2 evolving within the human population.

The interregional diversity measures the degree to which the virus can be categorized into region-specific subtypes. A demonstration of substantial interregional diversity would perhaps constitute the most compelling and concerning evidence of the potential for repeat epidemics. We developed two measures of interregional diversity. The first one is analogous to the intraregional diversity measure. We sampled pairs of isolates within each region and between each pair of regions within the same time window, computed the mean tree distance for both representative ensembles of these pairs (intraregional and interregional pairs), and calculated the ratio of interregional and intraregional values (*SI Appendix*, Fig. S16). The second one is a partition-level measure. For every pair of regions over each time window, we computed the Hellinger distance of the 11-group frequency distribution between all pairs of regions over each time window (see *Brief Methods* for details).

Both measures of interregional diversity support the division of the pandemic, through the beginning of 2021, into four periods (Fig. 5*C*). The first period that ended in February 2020 represents rapid diversification into region-specific phylogenies. This period was followed by a major extinction event and global homogenization ending in March 2020. The following 5 mo, March to July, represented a period of stasis, in terms of interregional diversity. Finally, July 2020 was the start of the ongoing period of interregional diversification.

The extinction of the earliest partitions, 1 and 2, corresponds to the advent of S|D614G, which became fixed in all other partitions and was globally ubiquitous by June 2020 (Fig. 5*D*). Partition 8, the only partition where N|203K and 204R were fixed, became dominant in every region outside of North America in the period that followed (*SI Appendix*, Fig. S17). However, this did not result in a global selective sweep that would involve the extinction of partitions 1 to 7. Instead, multiple NLS-associated mutations rose to prominence across different partitions, becoming globally dominant by September (Fig. 5*D* and *SI Appendix*, S18). To resolve this trend, at least two principal variants, N|203K/204R in partition 8 and N|220V in partition 5, have to be considered, and we identified six key amino acid replacements of interest for this period (N|203K/204R, N|220V, N|199L, N|194L, N205I, and N206F).

In the next phase of the pandemic, partition 8 dramatically fell from dominance in two regions, Africa and Oceania, replaced by partitions 6 and 7. Although we did not find a distinct mutational signature associated with the rise of partition 7 in Oceania (*SI Appendix*, Fig. S19), signatures associated with the rise of partition 6 were identified in both regions (*SI Appendix*, Figs. S20 and S21). Neither of these two groups of sequences (late sequences from partition 6, Oceania and Africa, respectively) form topologically distinct clades; however, due to the conserved mutational signatures, we considered both groups to represent distinct emerging variants, vOceania and vAfrica. The signature for vAfrica is consistent with variant B.1.351. Additionally, two divergent clades within partition 8 and one clade within partition 3 emerged.

The most prominent is clade v2 with a signature consistent with variant B.1.1.7. Altogether, resolving this trend of emerging substitutions in the RBD (Fig. 5*D* and *SI Appendix*, S22–S24) requires the consideration of at least three variants and includes 59 signature mutations. Clade v1 appeared first in Europe in April 2020; v2 also appeared in Europe, in September 2020; and v3 appeared in Asia and North America, in April 2020 (*SI Appendix*, Fig. S25). As discussed above, many VOC mutations appeared multiple times in more than one partition, and few of these mutations are phylogenetically informative. However, some key mutations show clear-cut epidemiological trends, which can be regionally defined. The region where a key mutation first emerged is not always the region where it first rises to prominence.

Thus, the key mutation S|501Y first emerged in May 2020 in Oceania, months before rising to global prominence in November. Additionally, S|501T (in the same site) began to rise to prominence in Oceania in November. S|484K first rose to prominence in South America in September 2020, while appearing in sequences from Europe and North America as early as July. Also, notably, although S|477N initially appeared in February/March 2020 in Europe, Oceania, and North America, it dramatically rose to prominence in Oceania in April, about 3 mo before this mutation became prominent elsewhere. S|477N is a signature mutation for v1 stemming from partition 3; however, the sequences from Oceania bearing this mutation from summer 2020 are in partition 8. The dramatic diversity of signature mutations among these variants decreases the likelihood of future selective sweeps (in the absence of bottlenecks in the total number of infected hosts) and increases the likelihood of repeat epidemics.

### The Impact of SARS-CoV-2 Diversification on Testing and Vaccination.

The ongoing diversification of SARS-CoV2 poses problems for both testing and vaccination. Substitutions in the E protein have already been demonstrated to interfere with a common PCR assay (73). Generally, ORF1ab is more conserved than the S protein, which itself is more conserved than the remaining ORFs (*SI Appendix*, Figs. S2 and S3). Using our SARS-CoV-2 MSA, we surveyed 10 regions from ORF1ab (5), N(4), and E(1) genes that are commonly used in PCR assays (74) for substitutions relative to the reference sequence. Among the more than 175,000 genome sequences, there were thousands of nucleotide substitutions in each of these regions, but those in ORF1ab were markedly less variable than those in N (*SI Appendix*, Table S5), with one region in N demonstrating variability in nearly one-third of all isolates. It can be expected that most targets within the polyprotein will remain subject to the fewest polymorphism-induced false negatives even as the virus continues to diversify.

Of the nine primary vaccines/candidates (75), three are inactivated whole virus (Sinovac, Wuhan Institute of Biological Products/Sinopharm, and Beijing Institute of Biological Products/Sinopharm), five utilize the entire spike protein as the antigen (Moderna/NIAID, CanSino Biological Inc./Beijing Institute of Biotechnology, University of Oxford/AstraZeneca, Gamaleya Research Institute, and Janssen Pharmaceutical Companies), and one utilizes only the RBD (Pfizer/Fosun Pharma/BioNTech). In addition to the greater sequence conservation of the spike protein relative to all other ORFs outside of the polyprotein, it is the principal host-interacting protein of SARS-CoV-2, making both the whole protein and the RBD obvious antigenic candidates. Most mutations in the RBD were demonstrated to decrease infectivity, but some conferred resistance to neutralizing antibodies (49). Multiple mutations in the RBD are signature mutations in emerging variants, and some have been demonstrated to result in neutralizing antibody evasion (51). Different choices of the antigen could result in more or less generalizable immunity to these variants.

## Conclusions

Virus evolution during a pandemic is a fast-moving target, so that, unavoidably, aspects of this analysis will be outdated by the time of publication. Nevertheless, several trends revealed here appear general and robust. Although it is difficult to ascertain positive selection for individual sites, the overall adaptive character of SARS-CoV-2 evolution involving multiple amino acid replacements appears to be beyond reasonable doubt. As expected, there are multiple positively selected sites in the S protein, but, more surprisingly, N protein includes several sites that appear to be strongly selected as well. The involvement of these adaptive substitutions in the nuclear localization of the N protein appears likely. Importantly, some of the mutations, for which positive selection was inferred, cooccur on multiple occasions and seem to form a robust epistatic network. Most likely, the effect of positive selection is manifested primarily at the level of epistatic interactions.

Clearly, despite the dramatic reduction in global travel (76), the evolution of SARS-Cov-2 is partly shaped by globalizing factors, including the increased virus fitness conferred by S|D614G, N|R203K&G204R, and other positively selected amino acid substitutions. However, we obtained strong evidence of both continuous virus diversification within geographic regions and “speciation,” that is, formation of stable, diverging, region-specific variants. This ongoing adaptive diversification could substantially prolong the pandemic and the vaccination campaign, in which variant-specific vaccines are likely to be required.

## Brief Methods

Please see *SI Appendix* for detailed methods. Genomes available as of January 8, 2021 were retrieved from the Gisaid (13) database, which includes most GenBank (77) and China National Center for Bioinformation (CNCB) (78) submissions. Sequences were clustered according to 100% identity with no coverage threshold, using the Cluster Database at High Identity with Tolerance (CD-HIT) software (79, 80), with ambiguous characters masked, and sequences shorter than 25,120 nucleotides were discarded. Sequences with more than 10 remaining interior, ambiguous characters were discarded. The alignment was constructed using a multithreaded compilation of the Multiple Alignment using Fast Fourier Transform (MAFFT) software (81), and sites corresponding to protein-coding ORFs were mapped to the alignment from the reference sequence NC_045512.2 excluding stop codons. The remaining sites were discarded, and out-of-frame gaps in the resulting alignment were amended through conversion to ambiguous characters or conversion of an entire codon to gaps as appropriate.

A fast, approximate phylogenetic tree was built using FastTree (82), defining an outgroup consisting of neighboring viral sequences, such as bat coronaviruses. Outliers based on the Hamming distance in the main group were discarded. Two reduced alignments were then constructed, with (nearly) invariant sites removed from the first one, and only the top 5% of sites with the most common substitutions relative to consensus retained in the second one.

Tree topology was iteratively established using both FastTree (82) and IQ-TREE (83). First, an ensemble of maximally diverse subtrees was constructed to span the smaller of the two reduced alignments, with topology optimized, using IQ-TREE. These trees were then converted into constraint files and merged to generate a single global constraint file for analysis with FastTree. This tree was then used to constrain the larger of the two reduced alignments, which, in turn, was used to constrain the global topology. The final tree was rooted at the outgroup. The coarse-grained tree topology was verified through a projection of the pairwise Hamming distance matrix, using the MATLAB (84) routine “cmdscale.”

Ancestral states were estimated using Fitch Traceback (16). Statistical associations between mutations were computed largely as previously described (35) Fig. 3 *D* and *E* (*SI Appendix*, Table S3 and Fig. S10). To assess the contribution of trinucleotide mutagenic contexts, mutations were divided into four classes: synonymous vs. nonsynonymous substitutions and substitutions with high vs. low frequency of independent occurrence (see *SI Appendix*, Fig. S4). This reconstruction was used to estimate *dN/dS* for all regions encoding mature proteins, in addition to Phylogenetic Analysis by Maximum Likelihood (PAML) (85).

Metadata were assigned according to Gisaid, with location abbreviations matched to latitude/longitude of a representative city for each location using simplemaps (https://simplemaps.com/data/world-cities) (86). Date and location data were used to construct time-varying intraregional and interregional phylogenetic divergence measures (Fig. 5*C* and *SI Appendix*, Fig. S16).

## Supplementary Material

Supplementary File

## Data Availability

The data has been deposited through Zenodo (10.5281/zenodo.5033811) (87). Previously published data were used for this work: GenBank (77), Gisaid (13), CNCB (78). In addition to *SI Appendix*, supplementary data can be found at https://ftp.ncbi.nih.gov/pub/wolf/_suppl/SARSevo21/ or https://doi.org/10.5281/zenodo.5033811 (87).
