# Supplementary material for: Ongoing global and regional adaptive evolution of SARS-CoV-2
Source: Proc Natl Acad Sci U S A. 2021 Jul 2;118(29):e2104241118. doi: 10.1073/pnas.2104241118 (PMC8307621; doi:10.1073/pnas.2104241118)
Supplement: Supplementary File [file pnas.2104241118.sapp.pdf]

PNAS
www.pnas.org

Supplementary Information for

# Ongoing Global and Regional Adaptive Evolution of SARS-CoV-2

Nash D. Rochman, Yuri I. Wolf, Guilhem Faure, Pascal Mutz, Feng Zhang, and Eugene V. Koonin

Nash D. Rochman
Email: nash.rochman@nih.gov

Feng Zhang
Email: zhang@broadinstitute.org

Eugene V. Koonin
Email: koonin@ncbi.nlm.nih.gov

**This PDF file includes:**

Detailed Methods
Figures S1 to S25
Tables S1 to S5

**Additional supplementary information can be found at this address:**

https://ftp.ncbi.nih.gov/pub/wolf/_suppl/SARSevo21/

This repository includes:

The global tree in Newick format: global_2_13_21.main.tre
The global, ultrametric tree in Newick format: global_2_13_21.ultra.tre
All metadata used in this study including the GISAID acknowledgements: metadata.tgz
Tables S1-S4 in plain text format.

## Methods

### Multiple alignment of SARS-CoV-2 genomes

All SARS-CoV-2 genomes that were available as of January 8, 2021 were retrieved from the Gisaid(1) database, which at that time comprised the vast majority of available sequences. Earlier iterations of this protocol had been previously completed from all available sequences including Genbank(2) and CNCB(3) as well. Sequences with apparent anomalies (sequence inversion etc.) were immediately discarded. Sequences were harmonized to DNA (e.g. U was transformed to T to amend software compatibility) and clustered according to 100% identity with no coverage threshold using CD-HIT(4, 5), with ambiguous characters masked. All characters excepting ACGT were considered ambiguous. The least ambiguous sequence from each cluster was selected and sequences shorter than 25120 nucleotides were discarded.

Exterior ambiguous characters (preceding/succeeding the first/last defined nucleotide) were removed, and sequences with more than 10 remaining interior, ambiguous characters were discarded. A reference alignment was previously constructed using the same protocol as follows with the exception of the --keeplength specification in November, 2020. The updated database was aligned using multi-threaded MAFFT(6) with 80 cores (--thread 80, when more cores were allocated they were not utilized) and 3.8Tb of RAM to maintain usage of the normal DP algorithm(6) (--nomemsave) against this reference alignment (specifying --keeplength). Aligning “from scratch” without --keeplength proved to be prohibitively slow so we recommend first constructing a reference alignment from a suitable subset of sequences. Sequences sourced from non-human hosts were manually identified from the metadata and those excluded at the previous step were added to the alignment using MAFFT, (again specifying --keeplength). Note that use of the --keeplength option will not include insertions relative to the reference alignment.

Sites corresponding to protein-coding ORFs were then mapped to the alignment from the reference sequence NC_045512.2 excluding stop codons as follows: 266-13468+13468-21552, orf1ab; 21563-25381, S; 25393-26217, orf3a; 26245-26469, E; 26523-27188, M; 27202-27384, orf6; 27394-27756, orf7a; 27756-27884, orf7b; 27894-28256, orf8; and 28274-29530, N. The remaining sites were discarded.

The resulting alignment contained out-of-frame gaps. Gaps in the reference sequence, corresponding to insertions, were found to correspond to gaps in all but fewer than 1% of the remaining sequences (all gaps in the reference sequence correspond to gaps in the alignment from November, 2020, the use of --keeplength prohibited the recognition of any insertions relative to the reference sequence which were not present in this reference alignment). These sites were discarded. The remaining gaps, corresponding to deletions relative to the

reference sequence, shorter than three nucleotides were replaced with the ambiguous character, N. Longer gaps were shifted into frame and padded with ambiguous characters on either end of the gap, minimizing the number of sites altered.

A fast, approximate tree was then built using FastTree(7) (parameters: -nt -gtr -gamma -nosupport -fastest) to unambiguously define two clusters of sequences: an outgroup consisting of 14 sequences sourced from non-human hosts prior to 2020 and the main group. The tree construction requires the resolution of very short branch lengths which makes it necessary to compile FastTree at double precision. Outliers from the remaining sequences were then identified based on the Hamming distance (excluding gaps and ambiguous characters) to the nearest neighbor, the Hamming distance to the consensus, and the degree to which those substitutions relative to consensus were clustered in the genome. At this step, 81 sequences were removed.

The resulting alignment, consisting of 98,090 sequences and 29,119 sites, was maintained for the construction of the global tree and ancestral sequence reconstruction. In an effort to minimize the impact of sequencing error on the tree topology, as well as to decrease computational costs, a reduced alignment was then constructed through the removal of 1) invariant sites, 2) sites invariant with the exception of a single sequence, and 3) sites invariant throughout the main group with the exception of at most one sequence representing each minority nucleotide. Removing these sites created substantial redundancy, so a representative sequence was selected for each cluster of 100% identity to yield an alignment consisting of 90,585 sequences and 16,487 sites. As described below and in the main text, a third alignment was constructed including only the top 5% of sites with the most common substitutions relative to consensus (of this second alignment) and again removing redundant sequences to yield 32,563 sequences and 834 sites.

## Tree Construction

We sought to optimize tree topology with IQ-TREE(8); however, building the global tree was computationally prohibitive, and thus, we proceeded to subsample the smallest alignment (834 sites) as follows. First, a core set of maximally diverse sequences is selected. The set is initialized with a pair of sequences: a sequence maximizing the number of substitutions relative to consensus and a paired sequence which maximizes the Hamming distance to itself. Sequences are then added to this core set one at a time maximizing the minimum Hamming distance to any representative of the set until *N* sequences are incorporated. Next, $ceil\left(L/(M-N)\right)$ resulting sets are initialized with this core set where *M* is the target number of sequences and *L* is the total number of sequences in the alignment (32,363). Then, sequences that have not yet been incorporated into any resulting set are added to each resulting set, again one at a time, maximizing the minimum distance to any representative of the set until *M*

sequences are incorporated. The order of the resulting sets is randomized at each iteration without repeats. Once every (main group) sequence has been incorporated into at least one resulting set, sequences are randomly incorporated into each set until every set contains *M* sequences. Finally, the outgroup is added to each resulting set. We chose *M*=3,000 in an effort to optimize computational efficiency and *N*=300.

Note that while increasing *N* increases the number of sets required for alignment coverage, and thus compute time, insufficient overlap between the sequences assigned each sub-alignment greatly affects the results of subsequent steps. As discussed in the main text, executing this protocol on an alignment containing most or all sites may not yield a consistent deep tree topology or "skeleton" since maximizing the hamming distance of any subset over all sites does not guarantee maximizing the tree distance in the resultant global topology. This is why limiting the alignment to sites with common substitutions relative to consensus is essential at this step.

A tree was then built, using IQ-TREE, for each maximally diverse set, with the evolutionary model fixed to GTR+F+G4 and the minimum branch length decreased from the default 10e-6 to 10e-7, according to the results of previous parameter studies(9). These trees were then converted into constraint files and merged to generate a single global constraint file for use within FastTree (parameters: -nt -gtr -gamma -cat 4 -nosupport -constraints).
The remaining sequences excluded from this tree but present in the second alignment (90,585 sequences and 16,487 sites) were then reintroduced as unresolved multifurcations and a new constraint file from the multifurcated tree was constructed. A second iteration of FastTree was initiated on the second alignment to produce an intermediate tree. This tree was primarily constructed as an intermediate step to limit the impact of sequencing errors on the final topology as mentioned in the main text; however, it is also less computationally intensive. The last step was then repeated on this intermediate tree to construct the global topology for the whole alignment. The final, global tree was rooted at the outgroup.

## Reconstruction of Ancestral Genome Sequences

Ancestral states were estimated by Fitch Traceback(10). Briefly, character sets were constructed from leaf to root where each node was assigned the intersection of the descendant character sets if not empty and the union otherwise. Then, moving from root to leaf, nodes with more than one character in their set were assigned the consensus character if present in their set or a randomly chosen representative character otherwise. Substitutions between states were identified and placed in the middle of the branch bridging the pair of nodes.

Statistical associations between mutations were computed in a manner similar to that previously described(11). Briefly, sequences were leaf-weighted based on the branch lengths of the ultrameterized, tree. Every mutation present across the tree at 200 mean leaf-weight equivalents or more was considered. The probability of independent co-occurrence between any pair was estimated in two ways. An arbitrary member of the pair was selected as the ancestral mutation, and the binomial probability:

$$\sum_{k=N_{pair}}^{N_{total}} \binom{N_{total}}{k} F^k (1-F)^{N_{total}-k}$$

was computed where *N_total* is the number of substitutions to the descendant mutation across the entire ancestral record, *N_pair* is the number of substitutions to the descendant which succeed or appear simultaneously with a substitution to the ancestral mutation, and *F* is the fraction of the tree (fraction of all applicable branch lengths) occupied by the ancestral mutation. The ancestral/descendent designation was then reversed and the “binomial score” was constructed as the negative log of the product of these two terms. Additionally, for each pair, the observed and expected (product of the tree fractions) tree intersections were calculated and the “Poisson score” (analogous to the log-odds ratio) was calculated:

$$\begin{cases} -\ln\left(1-PCDF(exp,obs)\right), obs > exp \\ \ln\left(PCDF(exp,obs)\right), obs < exp \end{cases}$$

where PCDF(exp,obs) is the cumulative probability of a Poisson distribution with mean “exp”, the expected value of the data, and evaluated at “obs”, the observed value of the data. Both scores are reported. SI Appendix, Table S3 displays putative positively selected mutations with both scores above 5 or at least two simultaneous substitutions. Fig. 1D only displays associations between mutations in the N or S proteins. SI Appendix, Fig. S10 does not exclude mutations with NCN context but meets all other statistical criteria for positive selection and does not display mutations in the polyprotein.

## Classical Multidimensional Scaling of the MSA

Pairwise Hamming distances were computed for all pairs of rows in the global MSA ignoring gaps and ambiguous characters i.e. the sequences *X*=”ATN-A” and *Y*=”NTAAT” would be assigned a distance of 1. The resulting distance matrix was embedded in three dimensions with the MATLAB(12) routine “cmdscale”. 100 rounds of stochastically initiated k-means clustering of the embedding was conducted and the optimum cluster number was determined to be 5 on the basis of the silhouette score distribution (SI Appendix, Fig S1). The silhouette score measures the similarity of each point to its own cluster relative to points in other clusters. For each point, it is defined as: min_over_clusters[(<d_other>-

<d_self>)/max(<d_other>,<d_self>)] where <d_other> is the mean distance from that value to values in a different cluster and <d_self> is the mean distance from that value to values in the same cluster. It ranges from -1 to 1.

## Validation of Mutagenic Contexts

Mutations were divided into four categories: synonymous vs non-synonymous substitutions and high vs low frequency of independent occurrence. For example, consider codon X with 3 non-synonymous substitutions gat->ggt and 1 non-synonymous substitution gat->cgt. In this context, a non-synonymous nucleotide substitution a->g of frequency 4 would be recorded in nucleotide (X-1)*3+2. The low vs high frequency threshold was determined by the 90$^{th}$ percentile of the synonymous mutation frequency distribution (operationally 7). For each mutation, the trinucleotide contexts from the ancestral reconstruction at the nodes where the mutation occurred were compared to the background genome-wide frequencies, computed for the inferred common ancestor of SARS-CoV-2.

The expected frequencies of the trinucleotides using the background distribution were tabulated; the Yates correction (+/-0.5 to the original count depending on whether the count is below or above the expectation) was applied to the observed frequencies; the log-odds ratios of the (corrected) observed frequencies to the expectation were computed; and CMDS was applied to the Euclidean distances between the log-odds vectors to embed the points onto a plane (SI Appendix, Fig. S4 A.). This analysis was then repeated, this time, distinguishing only between high and low frequency substitutions but not N and S (SI Appendix, Fig. S4 B). Finally, the differences in the contexts of high frequency synonymous vs non-synonymous events were considered in the same manner and the chi-square statistics ((observed-expected)^2/expected) were compared with the critical chi-square value (p=0.05/64, df=1, SI Appendix, Fig. S4 C.).

## Computation of *dN/dS*

For each of the 24 ORFs (splitting orf1ab into 15 segments corresponding to the 15 mature proteins, nsp11 and nsp12 combined), 10 reduced alignments were constructed as follows. Sequences were ordered based on diversity, in the same order with which they were included in the constraint trees. The first 10 sequences are conserved across every alignment and the remaining 40 are unique to each alignment. The reference sequence, NC_045512.2, was additionally added to each reduced alignment. PAML(13) was then used to estimate tN, tS, *dN/dS*, N, S, and N/S for each segment and every reduced alignment.

Given the global ancestral reconstruction from Fitch traceback, the total number of non-synonymous and synonymous substitutions (nN and nS, respectively) as well as these tallies normalized by the respective segment length (tN, and tS, respectively) were retrieved for each segment. A hybrid *dN/dS* value for each

segment was estimated to be (nN/nS)/(N/S)* where (N/S)* is the median value of N/S across all repeats for the segment.

## Metadata Assignment

Headers for all isolates belonging to CD-HIT clusters with a representative incorporated into the alignment with fewer than 10 interior ambiguous characters were processed to extract the sequencing date and location. Sequencing location abbreviations were matched to full names and the latitude/longitude of a representative city for each location was retrieved from simplemaps (https://simplemaps.com/data/world-cities)(14).

## Regional Divergence Analysis

Two approaches, one partition dependent and one partition independent, were used as described in the main text. The Hellinger distance between regions over a sliding time window was computed between regions for the 11 (partitions/variant clades) group distribution. Next, 400 isolates were randomly selected from each region over a sliding window and 200 pairs within each region as well as 200 pairs between each pair of regions were composed. The tree distance between each pair was computed and the mean for each inter- and intra-regional pair tree-distance distribution was recorded. In Figs. 3C and S16, the 25$^{th}$, 50$^{th}$, and 75$^{th}$ percentiles are shown of the 15 possible pairs of (6) regions. Regions are selected based on GISAID metadata. The inter-regional tree divergence (Figs. 3C, top and SI Appendix, S16C) is reported as the ratio between the mean of the inter-regional pair tree-distance and the mean of the intra-regional pair tree distances across both regions.

## Supplemental Figures

**Figure S1.** 25th, median (solid line), and 75th percentiles of the silhouette score distribution for 100 stochastically initiated rounds of k-means clustering for 2-16 clusters and a projection of the 3D embedding of the pairwise Hamming distance matrix between SARC-CoV-2 genomes. Partitions are color-coded and wires enclose the convex hulls for each of the five optimal clusters.

**Figure S2. A.** Distributions of the moving average, respecting segment boundaries, across a 100 codon window for synonymous (blue) and nonsynonymous (amino acid replacing) substitutions (orange). Solid lines: normal approximations of the distributions (same median and interquartile distance); solid lines: approximation with the same median and theoretical (Poisson) variance. **B.** Moving averages, respecting segment boundaries, across a 100 codon window for synonymous and nonsynonymous substitutions per site, raw (top) and normalized by the median (bottom). There are several regions in the genome with an apparent dramatic excess of synonymous substitutions: 5' end of orf1ab gene; most of the M gene; 3'-half of the N gene, as well as amino acid substitutions: most of the orf3a gene; most of the orf7a gene; most of the orf8 gene; and several regions in of the N gene.

**Figure S3.** Moving average over a window of 1000 codons, not respecting segment boundaries, of the total number of nucleotide exchanges n1->n2 summed over all substitutions. The ratio to the median over the entire alignment is also displayed as well as the normalized exchange distribution *(i.e.* #c->u/(#c->u+#c->g+#c->a)).

**Figure S4 A.** Two dimensional embedding of the Euclidean distances between the log-odds vectors of low and high frequency, nonsynonymous and synonymous mutations in the space of trinucleotide contexts relative to background expectation. The context of the high-frequency events (both S and N) is dramatically different from the background frequencies. There is a strong common component in the deviation of both kinds of high-frequency events. The context of the low-frequency events (both S and N) also differs slightly, in the same direction, from the background frequencies. There is a consistent distinction between synonymous and non-synonymous events, suggesting that a single mutagenic context or mechanistic bias does not account for both S and N events. **B.** Log odds ratio of low and high frequency mutations, both synonymous and nonsynonymous, relative to background expectation for each trinucleotide context. The NCN context (i.e. all mutations C->D) harbors dramatically more mutation events than the other contexts (all 16 NCN events are within the top 20 most-biased high-frequency events). The log-odds ratios for low-frequency events are poorly correlated with those for high-frequency events, suggesting that different mechanisms may be responsible for the strong bias observed among high frequency events and the weaker bias observed among low frequency events. **C.** Log odds ratio of high frequency nonsynonymous mutations

relative to the background expectation from the sum of both high synonymous and high nonsynonymous mutations vs. the sum + 1. There are 20 contexts where synonymous and non-synonymous events differ significantly (chi-sq> 11.28). 2/9 contexts with an excess of non-synonymous events are NCN (gct,tct). The remaining 7 are NGN (agt,gga,aga,ggt,agc,tgt). This additionally suggests that these non-synonymous events could be driven by other mechanisms. There is no correlation between the frequency of event context and the log-odds ratio for non-synonymous events, further suggesting that the log-odds ratio is not biased by hot-spot mutation context.

**Figure S5.** Correspondence between the “tree length for dN”, “tree length for dS”, and *dN/dS* between PAML and the results of the ancestral reconstruction utilizing Fitch traceback across 24 ORFs. Three high outliers in the PAML tS distribution are identified in the third plot and omitted from the first two.

**Figure S6. A.** The number of nonsynonymous events vs the number of synonymous events per codon. **B.** The moving average of 100 codons, respecting segment boundaries. **C.** The moving average after removing outlier high frequency events. Rho refers to Spearman. Dashed lines are 2/1.3*x reflecting the genome-wide ratio of nonsynonymous to synonymous substitutions, solid lines are linear best fit. Red points correspond to the middle third of the N protein.

**Figure S7.** Moving averages across a 100 codon window for synonymous and nonsynonymous substitutions per site in the N protein after removing outlier high frequency events. The nonsynonymous substitution frequencies in the center of the protein are not elevated relative to either terminus.

**Figure S8.** The fraction of sites with at least one substitution vs moving averages, respecting segment boundaries, over windows of 100 codons for synonymous and nonsynonymous substitutions.

**Figure S9.** Site history trees for spike 69 as drawn in Fig. 1C.

**Figure S10.** Epistatic network for the tree including mutations with NCN context and meeting all other criteria for positive selection. Mutations in the polyprotein are not displayed.

**Figures S11.** Correlation between sequencing date and tree distance to the root for all isolates with metadata as well as those which appear explicitly in the tree.

**Figures S12-14.** Global distribution of sequences. Color represents the number of sequences from that location and size represents the fraction of sequences from the clade displayed. Partition indices are in the top left corner of each map.

**Figure S15.** Regional SARS-CoV-2 partition dynamics during the COVID-19 pandemic (absolute number of sequences shown in contrast to Fig. 2).

**Figure S16.** The mean tree distance between pairs of isolates **A.** from different regions, **B.** within the same region (averaged over both regions in each pair) and **C.** The ratio over time (see Methods). 25$^{th}$, 50$^{th}$, and 75$^{th}$ percentiles of all 15 pairs of 6 regions. The ratio reported is between the mean of the inter-regional pair tree-distance and the mean of the intra-regional pair tree distances across both regions for each pair of regions.

**Figure S17.** Regional distributions of major partitions in the global topology March vs. July and July vs. November.

**Figure S18.** The frequencies of NLS-associated mutations N|194L, N119L, N203K, N205I, and N220V over time and across geographic regions along with S|614G for reference.

**Figure S19.** The Kullback-Leibler divergence and sequence logo for the 15 most divergent codons in sequences sourced after October 15, 2020 from Oceania in partition 7 vs. all sequences from Oceania in partition 7.

**Figure S20.** The Kullback-Leibler divergence and sequence logo for the 15 most divergent codons in sequences sourced after November 1, 2020 from Oceania in partition 6 vs. all sequences from Oceania in partition 6.

**Figure S21.** The Kullback-Leibler divergence and sequence logo for the 15 most divergent codons in sequences sourced after November 1, 2020 from Africa in partition 6 vs. all sequences from Africa in partition 6.

**Figures S22-24.** The frequencies of variant-associated mutations in the spike protein over time and geographic regions.

**Figure S25.** Regional SARS-CoV-2 variant clade dynamics during the COVID-19 pandemic (log of absolute number of sequences shown).

## Supplemental Tables

**Table S1.** The list of all mutations either in the top 100 most commonly observed or top 100 with the greatest number of parallel substitutions ordered as they appear in the genome.

**Table S2.** List of sites most likely to be evolving under positive selection. For List 2 the average tree fraction descendant from each candidate positively selected amino acid replacement must be sufficiently large(see Methods).

**Table S3.** All epistatic interactions among states meeting the criteria outlined in the main text for likely positive selection with binomial/Poisson scores greater than 5 or at least 2 simultaneous substitutions. Each mutation must have a minimum weight of approximately 200 leaves and each pair, 100 leaves. Each pair is arbitrarily ordered and the numbers of simultaneous, descendant, and independent substitutions are tabulated.

**Table S4. List 1**. List of variant mutations and variant IDs sorted by the number of variant ID's assigned to each mutation. **List 2**. List of all pairs of mutations associated with a single variant ID (internal variant ID's excluded. **List 3**. List of putative epistatic interactions between variant mutations and other states in the tree.

**Table S5.** The number of isolates (out of approximately 175k) observed to bear at least one substitution relative to the reference sequence, NC_045512.2, within the regions specified. These regions are commonly used within PCR assays for diagnostic testing.

## References


1. Elbe S & Buckland-Merrett G (2017) Data, disease and diplomacy: GISAID's innovative contribution to global health. *Global Challenges* 1(1):33-46.
2. Benson DA, *et al.* (2012) GenBank. *Nucleic acids research* 41(D1):D36-D42.
3. Zhao W-M, *et al.* (2020) The 2019 novel coronavirus resource. *Yi chuan= Hereditas* 42(2):212-221.
4. Fu L, Niu B, Zhu Z, Wu S, & Li W (2012) CD-HIT: accelerated for clustering the next-generation sequencing data. *Bioinformatics* 28(23):3150-3152.
5. Li W & Godzik A (2006) Cd-hit: a fast program for clustering and comparing large sets of protein or nucleotide sequences. *Bioinformatics* 22(13):1658-1659.
6. Katoh K, Misawa K, Kuma Ki, & Miyata T (2002) MAFFT: a novel method for rapid multiple sequence alignment based on fast Fourier transform. *Nucleic acids research* 30(14):3059-3066.
7. Price MN, Dehal PS, & Arkin AP (2010) FastTree 2–approximately maximum-likelihood trees for large alignments. *PloS one* 5(3):e9490.
8. Nguyen L-T, Schmidt HA, Von Haeseler A, & Minh BQ (2015) IQ-TREE: a fast and effective stochastic algorithm for estimating maximum-likelihood phylogenies. *Molecular biology and evolution* 32(1):268-274.
9. Morel B, *et al.* (2020) Phylogenetic analysis of SARS-CoV-2 data is difficult. *bioRxiv*.
10. Fitch WM (1971) Toward defining the course of evolution: minimum change for a specific tree topology. *Systematic Biology* 20(4):406-416.
11. Rochman ND, Wolf YI, & Koonin EV (2020) Deep phylogeny of cancer drivers and compensatory mutations. *Communications biology* 3(1):1-11.
12. MathWorks I (1992) *MATLAB, high-performance numeric computation and visualization software: reference guide* (MathWorks).
13. Yang Z (2007) PAML 4: phylogenetic analysis by maximum likelihood. *Molecular biology and evolution* 24(8):1586-1591.
14. Simplemaps (World Cities Database.

# S1

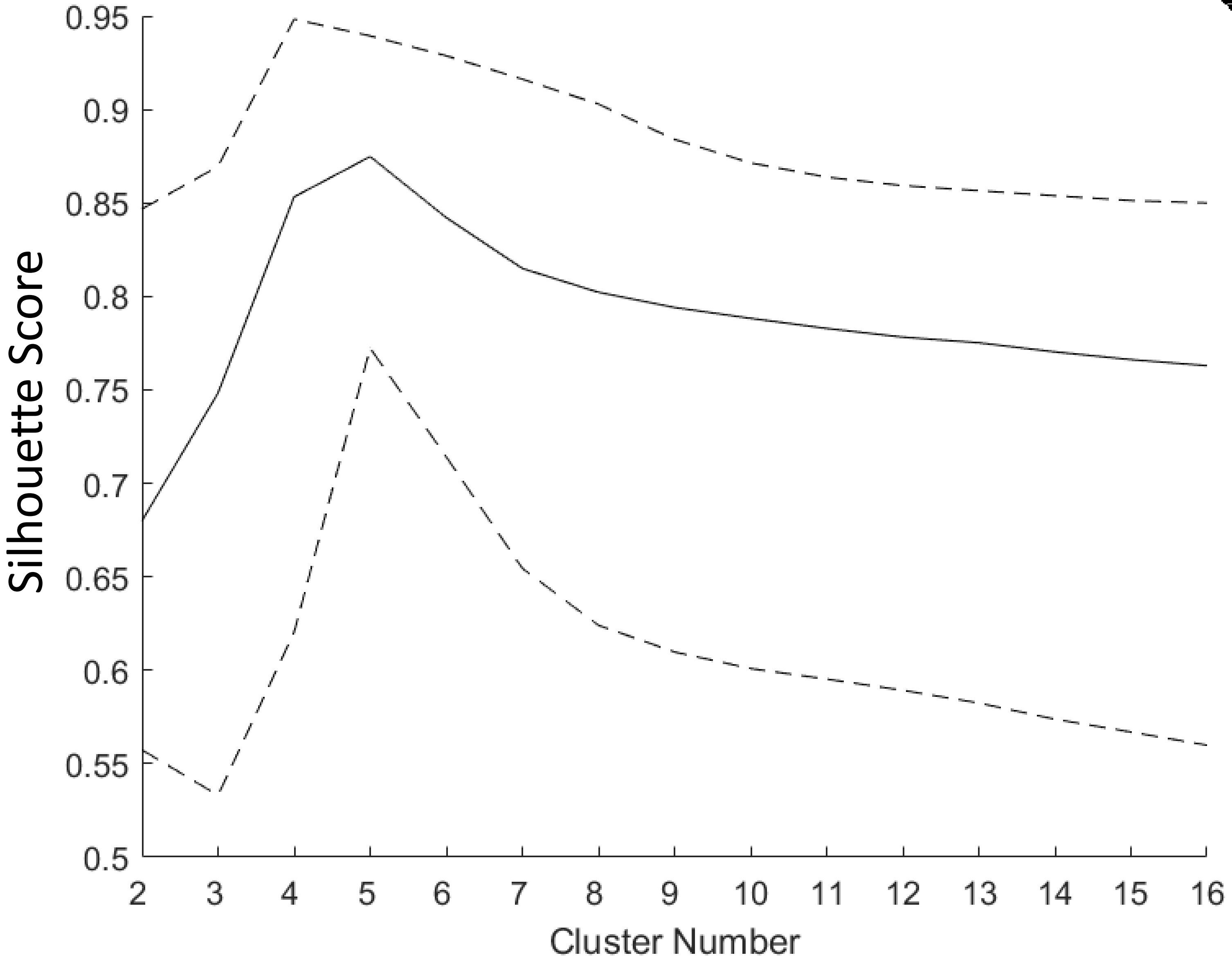
Silhouette Score
0.95
0.9
0.85
0.8
0.75
0.7
0.65
0.6
0.55
0.5
2
3
4
5
6
7
8
9
10
11
12
13
14
15
16
Cluster Number

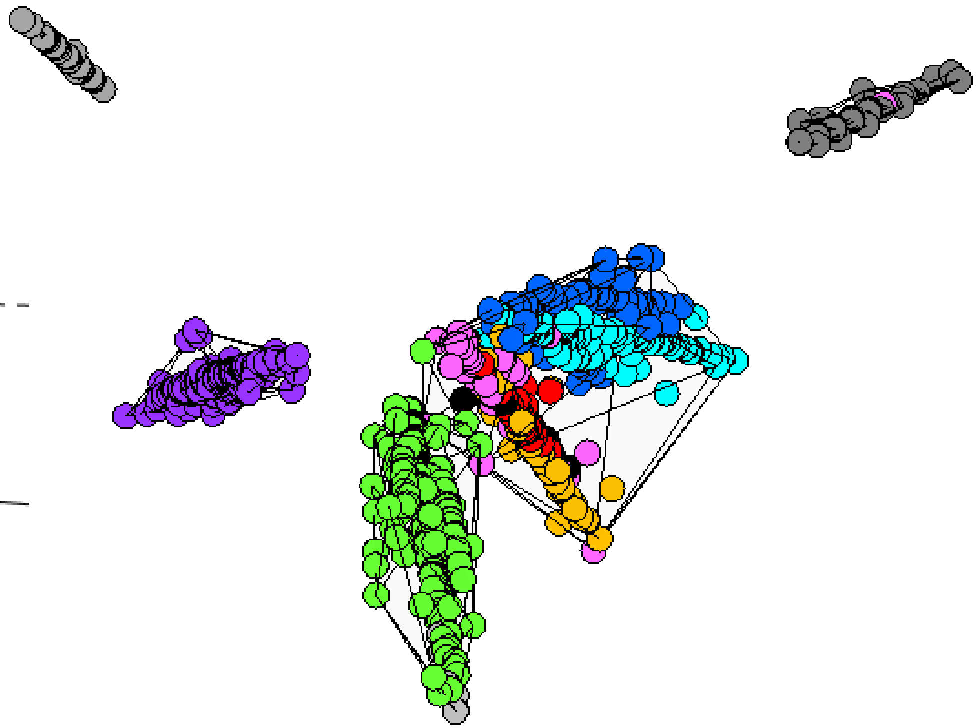

# S2

## A

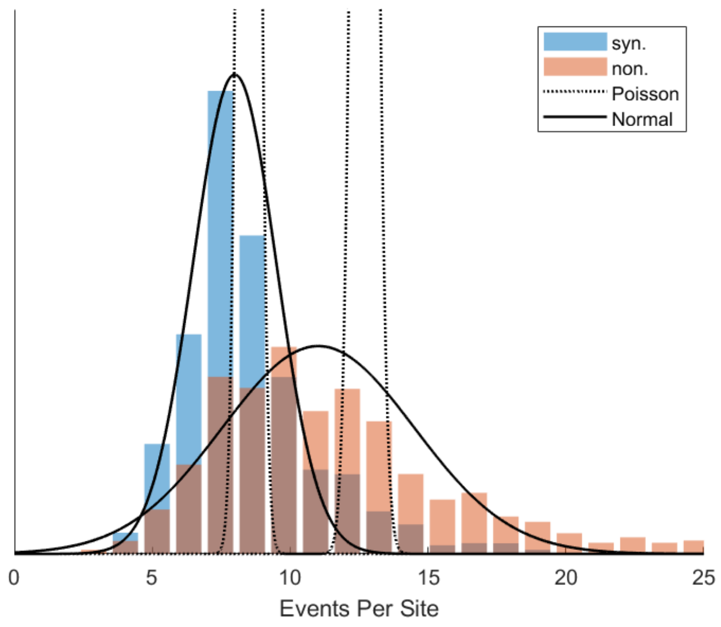

syn.
non.
Poisson
Normal
0
5
10
15
20
25
Events Per Site


## B

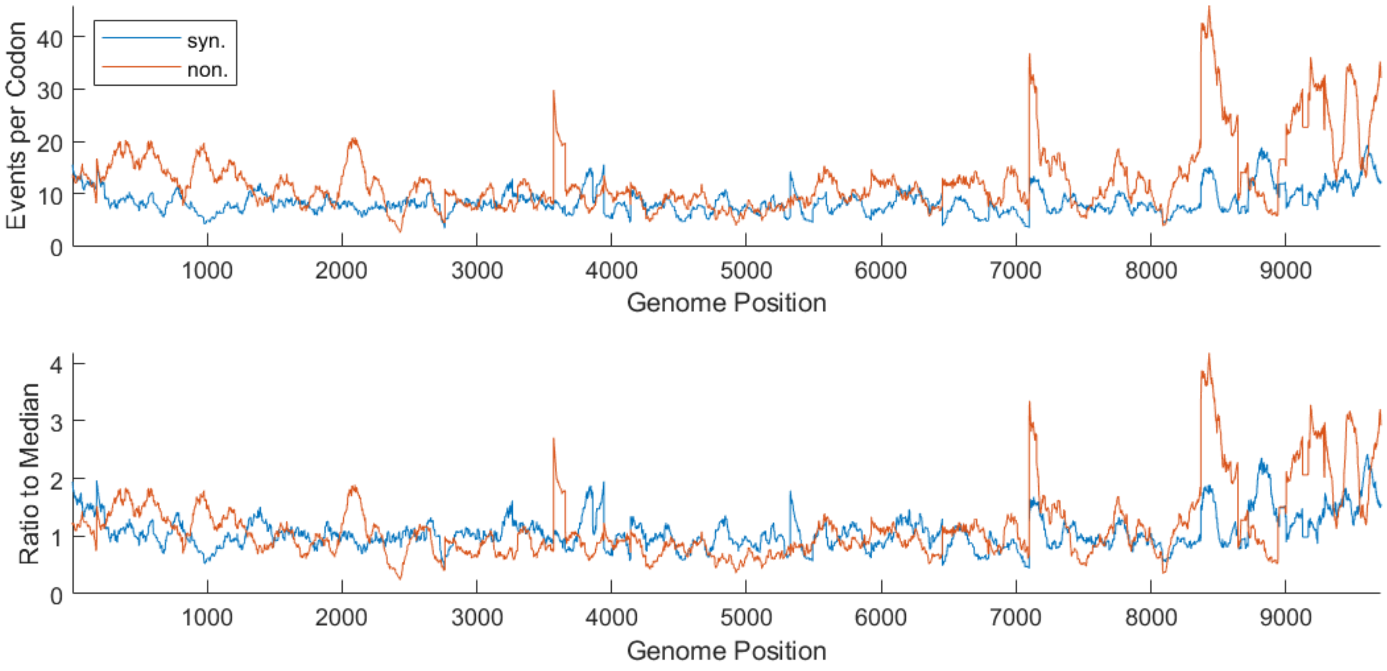

syn.
non.
Events per Codon
0
10
20
30
40
1000
2000
3000
4000
5000
6000
7000
8000
9000
Genome Position
Ratio to Median
0
1
2
3
4
1000
2000
3000
4000
5000
6000
7000
8000
9000
Genome Position

# S3

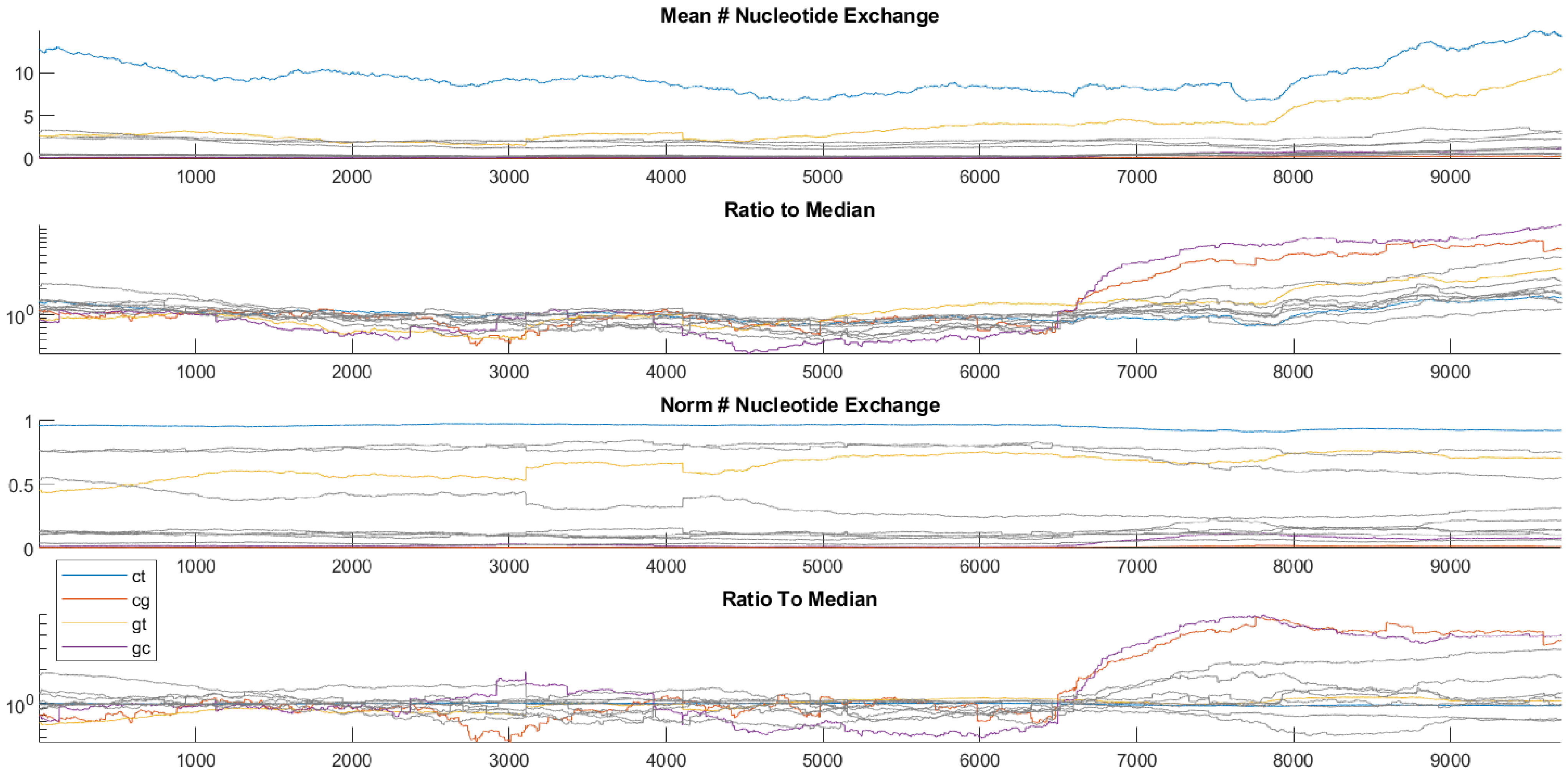

Mean # Nucleotide Exchange
10
5
0
1000
2000
3000
4000
5000
6000
7000
8000
9000
Ratio to Median
$10^0$
1000
2000
3000
4000
5000
6000
7000
8000
9000
Norm # Nucleotide Exchange
1
0.5
0
1000
2000
3000
4000
5000
6000
7000
8000
9000
ct
cg
gt
gc
Ratio To Median
$10^0$
1000
2000
3000
4000
5000
6000
7000
8000
9000

# S4

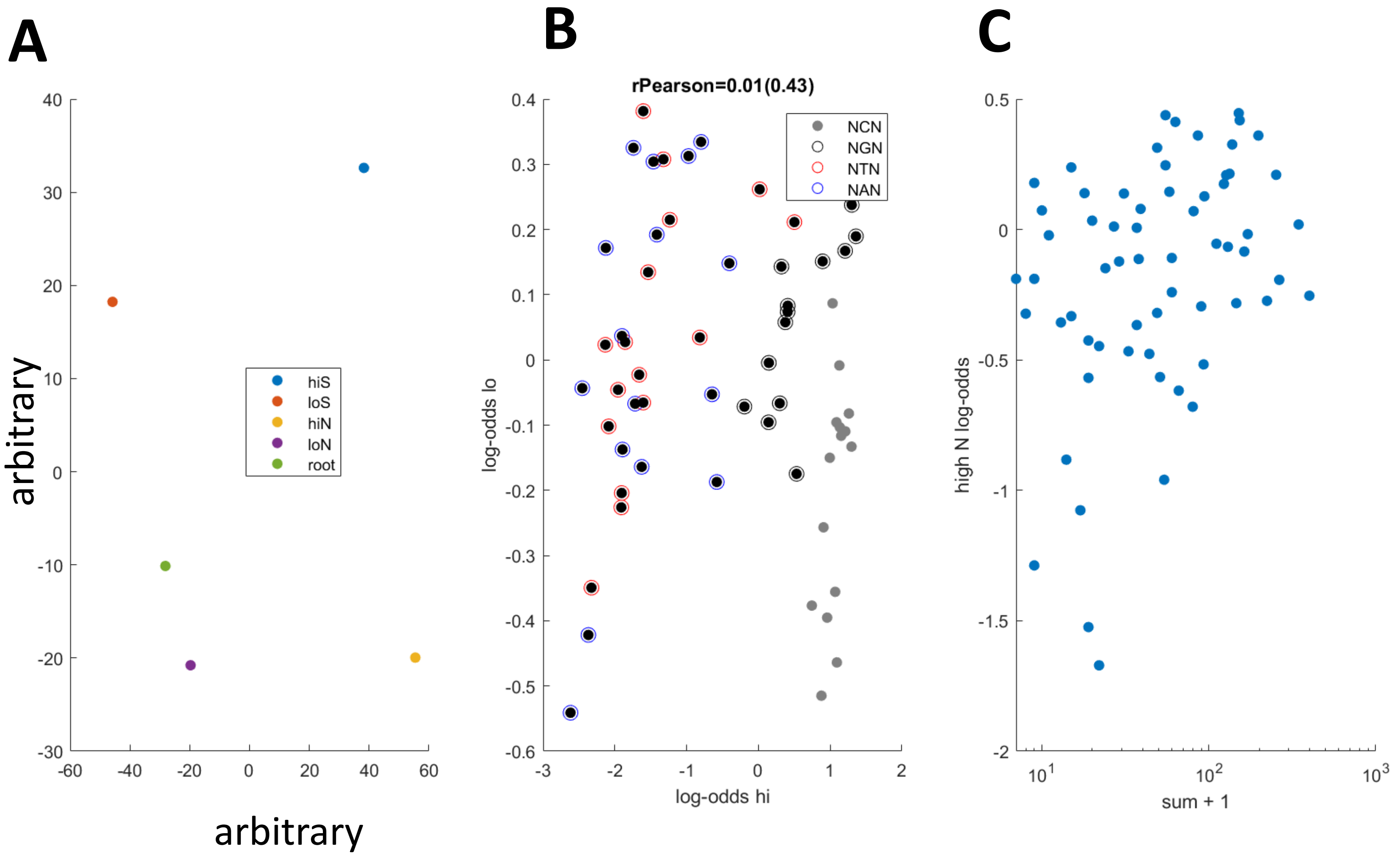


| context | log-odd S | log-odd N | chi-sq |
|---|---|---|---|
| agt | -2.04 | 0.45 | 72.63 |
| gga | -2.13 | 0.44 | 26.41 |
| aga | -1.63 | 0.42 | 62.85 |
| ggt | -1.68 | 0.41 | 25.9 |
| tgt | -1.09 | 0.36 | 55.91 |
| agc | -1.14 | 0.36 | 25.07 |
| tgc | -0.9 | 0.33 | 30.98 |
| gct | -0.42 | 0.21 | 19.83 |
| tct | 0.26 | -0.19 | 14.04 |
| aca | 0.32 | -0.25 | 34.32 |
| tca | 0.34 | -0.27 | 21.31 |
| cca | 0.34 | -0.28 | 14.46 |
| atc | 0.5 | -0.52 | 23.85 |
| gcg | 0.51 | -0.57 | 14.36 |
| tcg | 0.54 | -0.62 | 21.6 |
| ata | 0.58 | -0.68 | 30.33 |
| atg | 0.67 | -0.96 | 31.4 |
| cta | 0.76 | -1.52 | 16.31 |
| ttg | 0.79 | -1.67 | 20.8 |

# S5

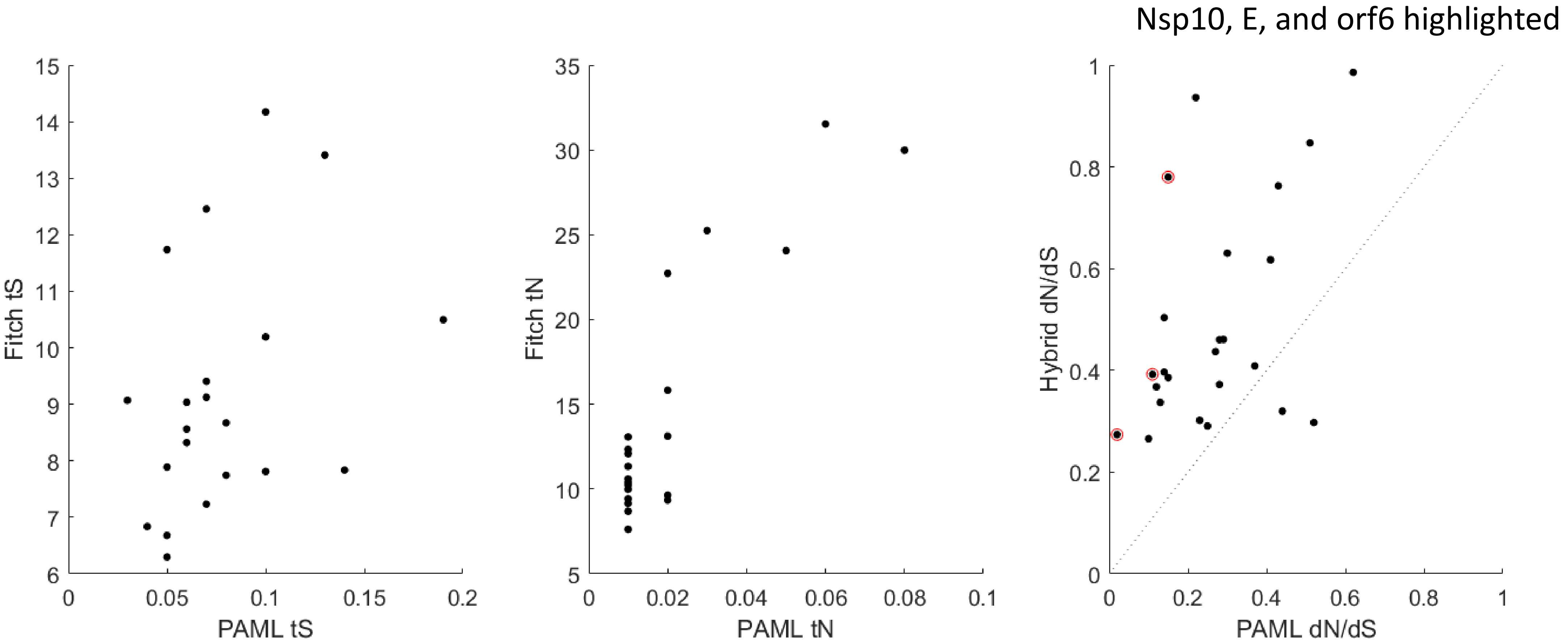

Nsp10, E, and orf6 highlighted
Fitch tS
PAML tS
Fitch tN
PAML tN
Hybrid dN/dS
PAML dN/dS

S6

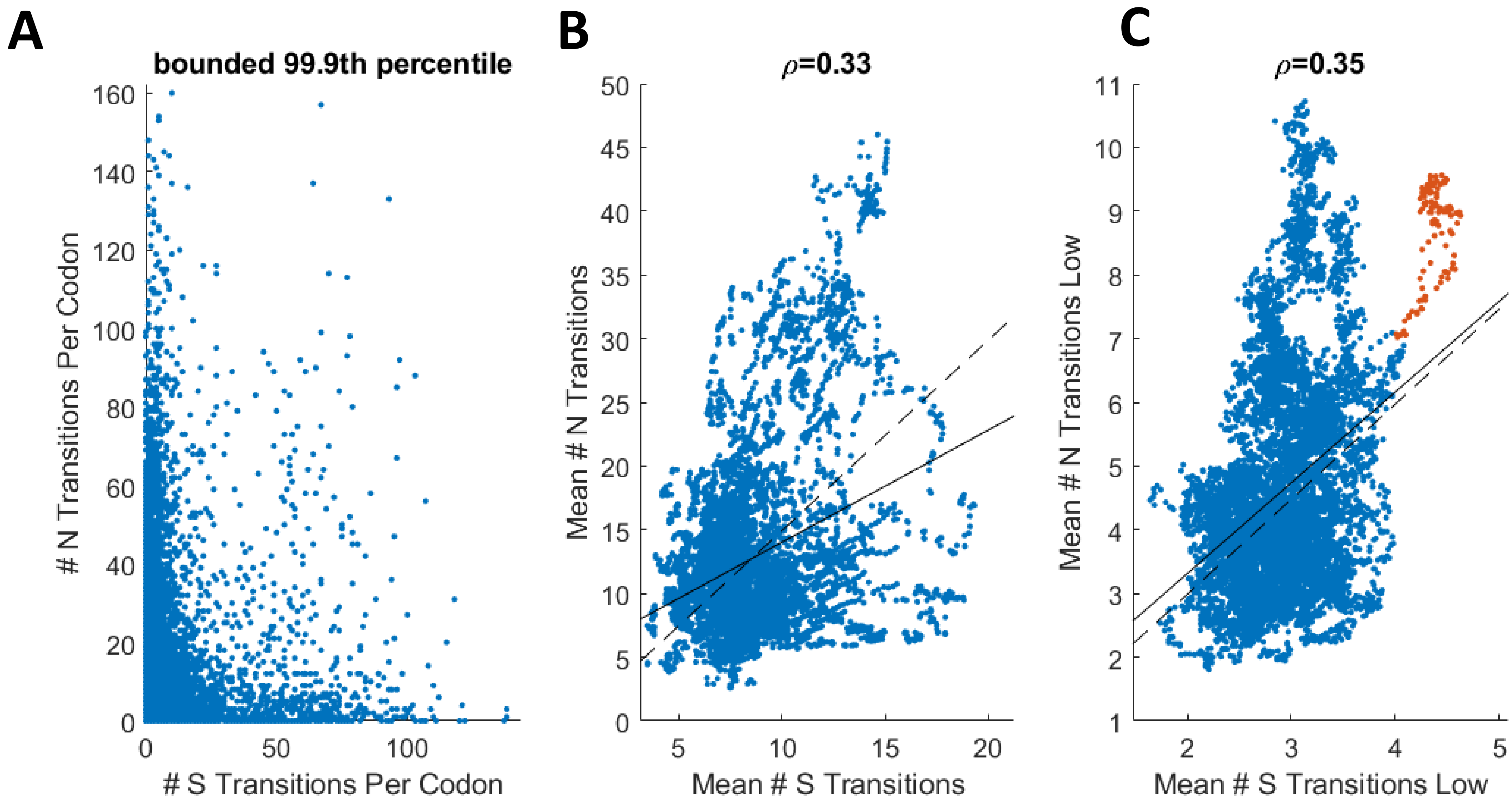
A
bounded 99.9th percentile
# N Transitions Per Codon
# S Transitions Per Codon
0
20
40
60
80
100
120
140
160
0
50
100
B
ρ=0.33
Mean # N Transitions
Mean # S Transitions
0
5
10
15
20
25
30
35
40
45
50
5
10
15
20
C
ρ=0.35
Mean # N Transitions Low
Mean # S Transitions Low
1
2
3
4
5
6
7
8
9
10
11
2
3
4
5

**S7**

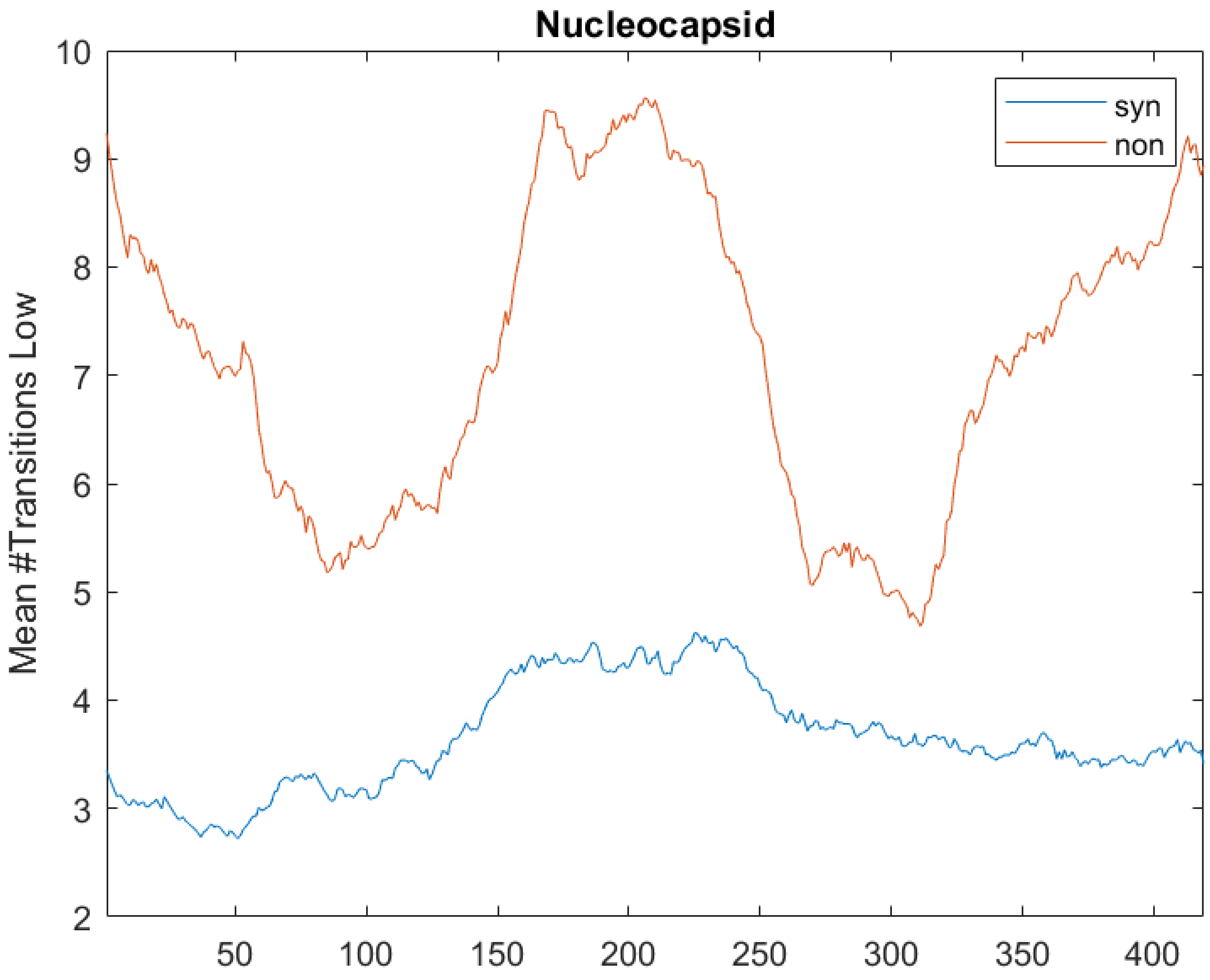
Nucleocapsid
Mean #Transitions Low
syn
non
10
9
8
7
6
5
4
3
2
50
100
150
200
250
300
350
400

**S8**

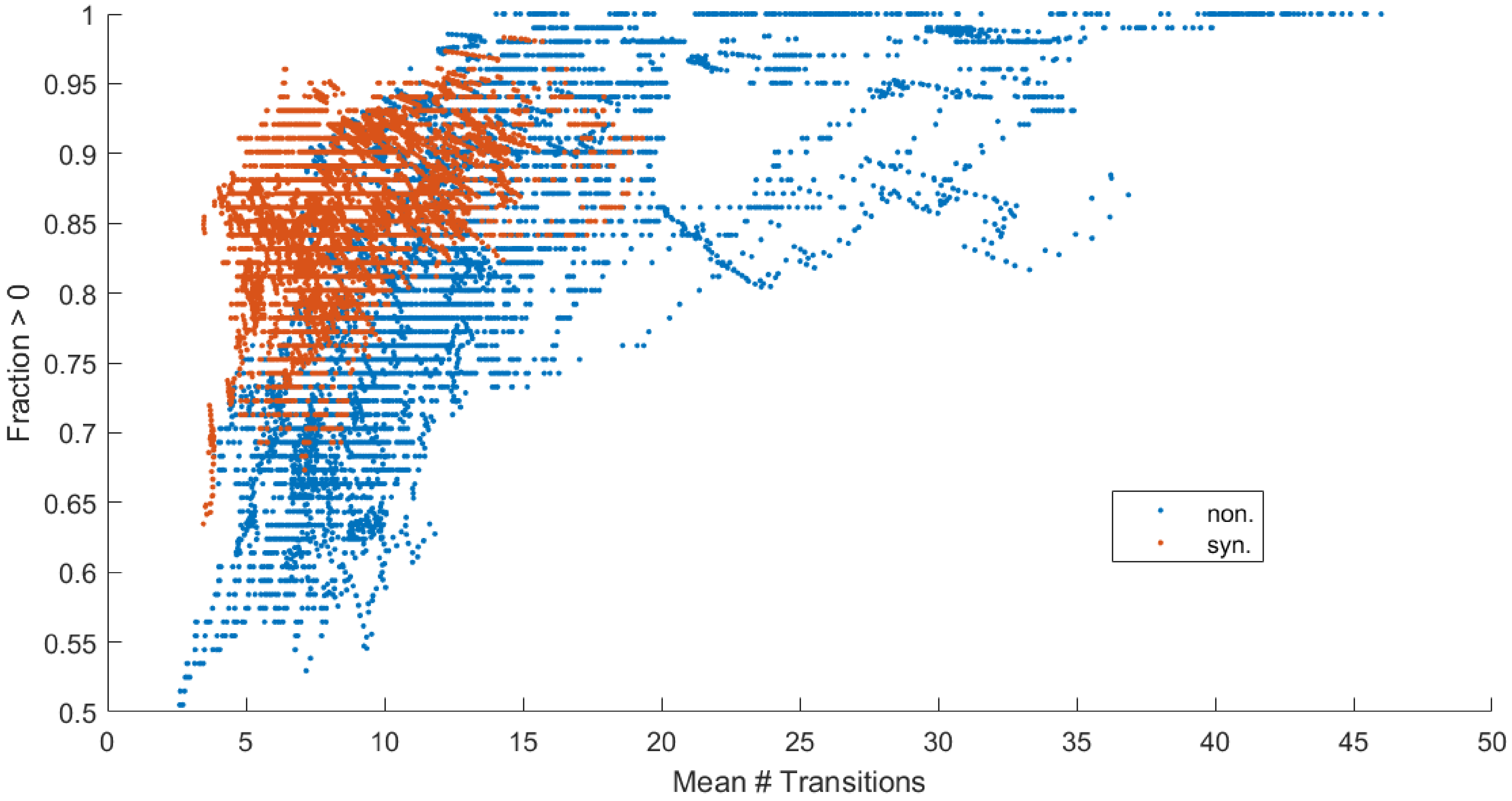

1
0.95
0.9
0.85
0.8
0.75
0.7
0.65
0.6
0.55
0.5
Fraction > 0
0
5
10
15
20
25
30
35
40
45
50
Mean # Transitions
non.
syn.

# S9

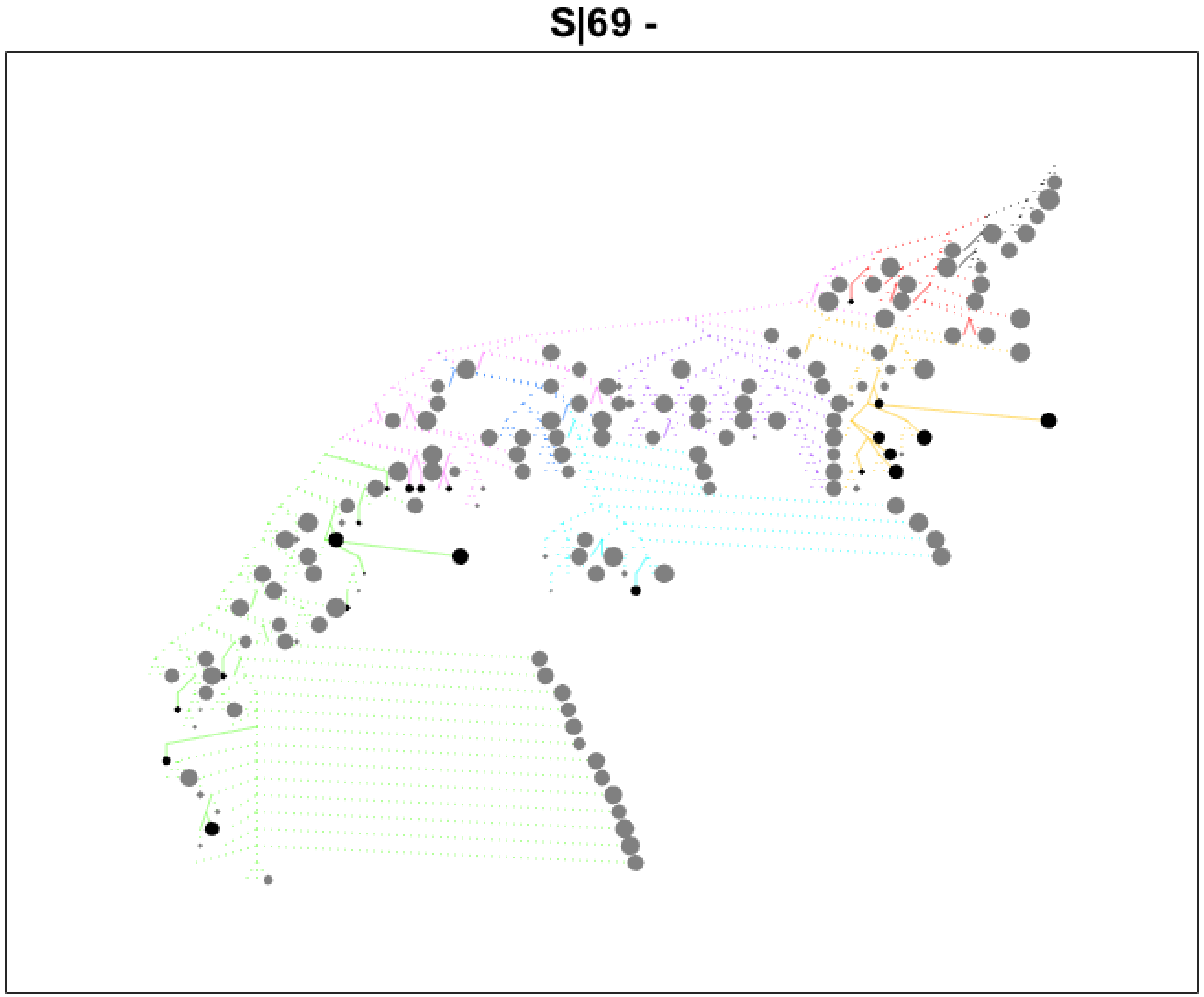

# S10

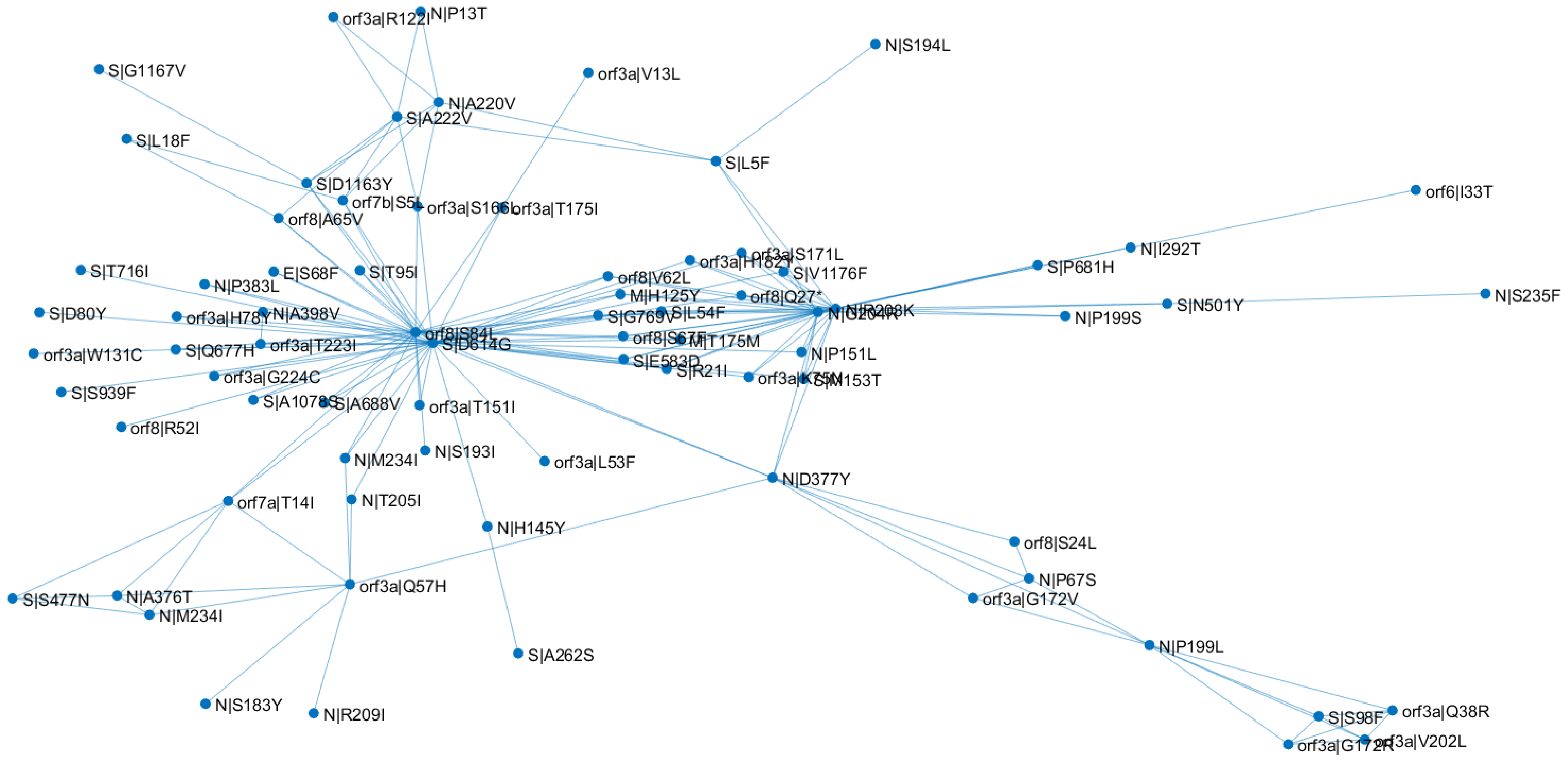

orf3a|R122I
N|P13T
N|S194L
S|G1167V
orf3a|V13L
N|A220V
S|A222V
S|L18F
S|L5F
S|D1163Y
orf7b|S5L
orf3a|S166L
orf3a|T175I
orf8|A65V
orf6|I33T
orf3a|S171L
orf3a|H182Y
N|I292T
S|T716I
E|S68F
S|T95I
orf8|V62L
S|V1176F
S|P681H
N|P383L
M|H125Y
orf8|Q27*
S|N501Y
N|S235F
S|D80Y
orf3a|H78Y
N|A398V
S|G769V
S|L54F
N|P199S
orf8|S84L
orf8|S67F
M|T175M
orf3a|W131C
S|Q677H
orf3a|T223I
S|D614G
N|P151L
S|E583D
orf3a|G224C
S|R21I
S|S939F
S|A1078S
S|A688V
orf3a|T151I
orf8|R52I
N|M234I
N|S193I
orf3a|L53F
N|D377Y
orf7a|T14I
N|T205I
N|H145Y
orf8|S24L
N|P67S
orf3a|G172V
S|S477N
N|A376T
orf3a|Q57H
N|M234I
N|P199L
S|A262S
S|S98F
orf3a|Q38R
orf3a|V202L
N|S183Y
N|R209I

# S11

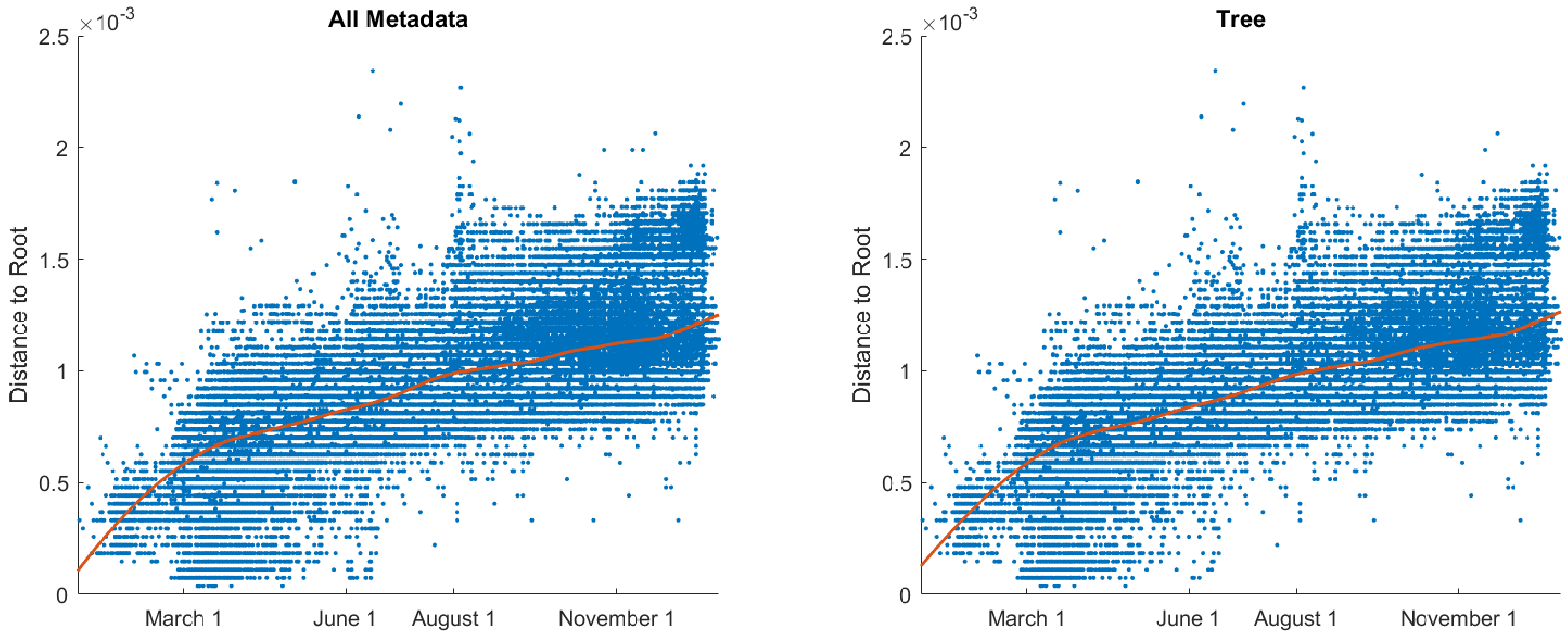
All Metadata
Tree
×10^-3
2.5
2
1.5
1
0.5
0
Distance to Root
March 1
June 1
August 1
November 1

# S12

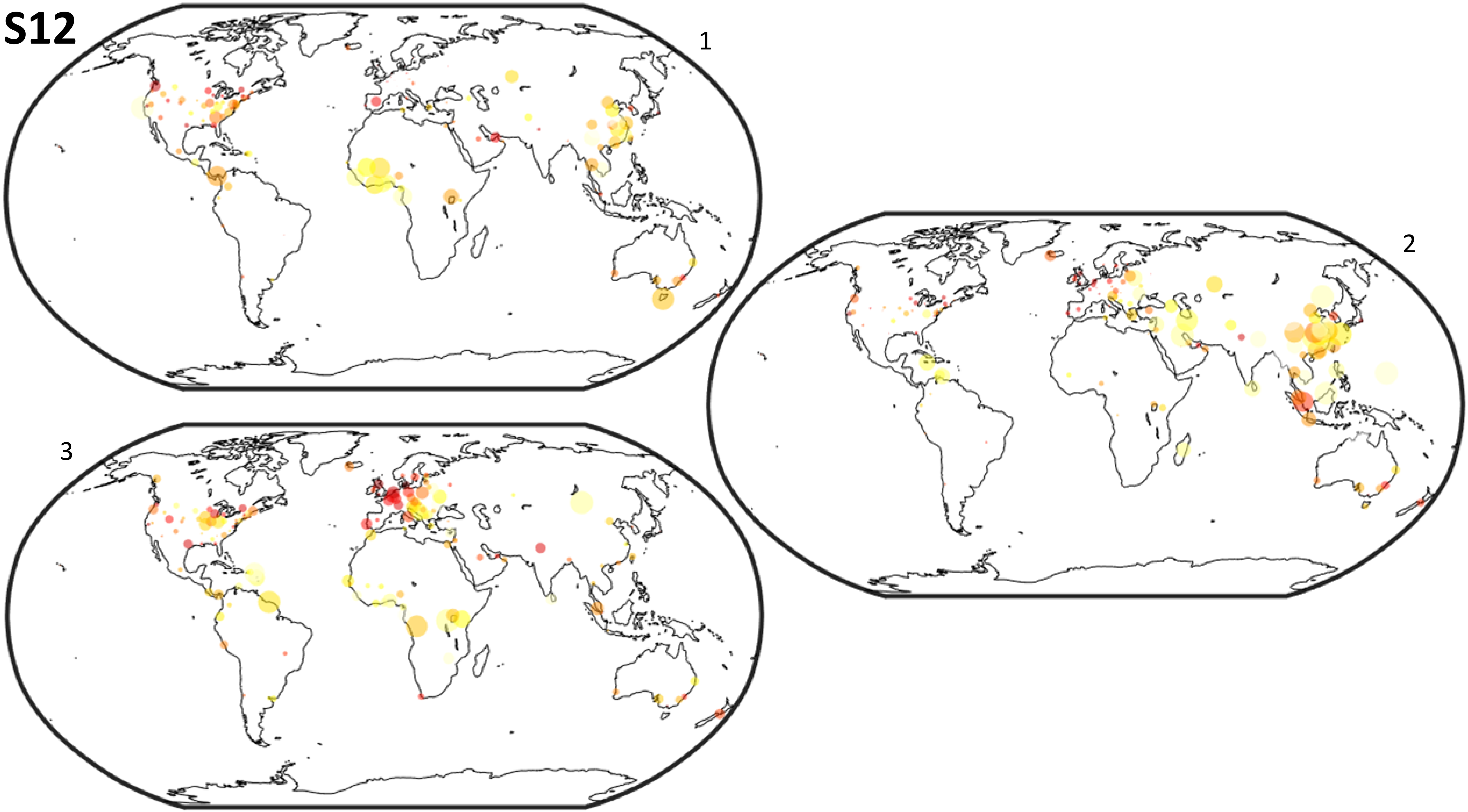

**S13**

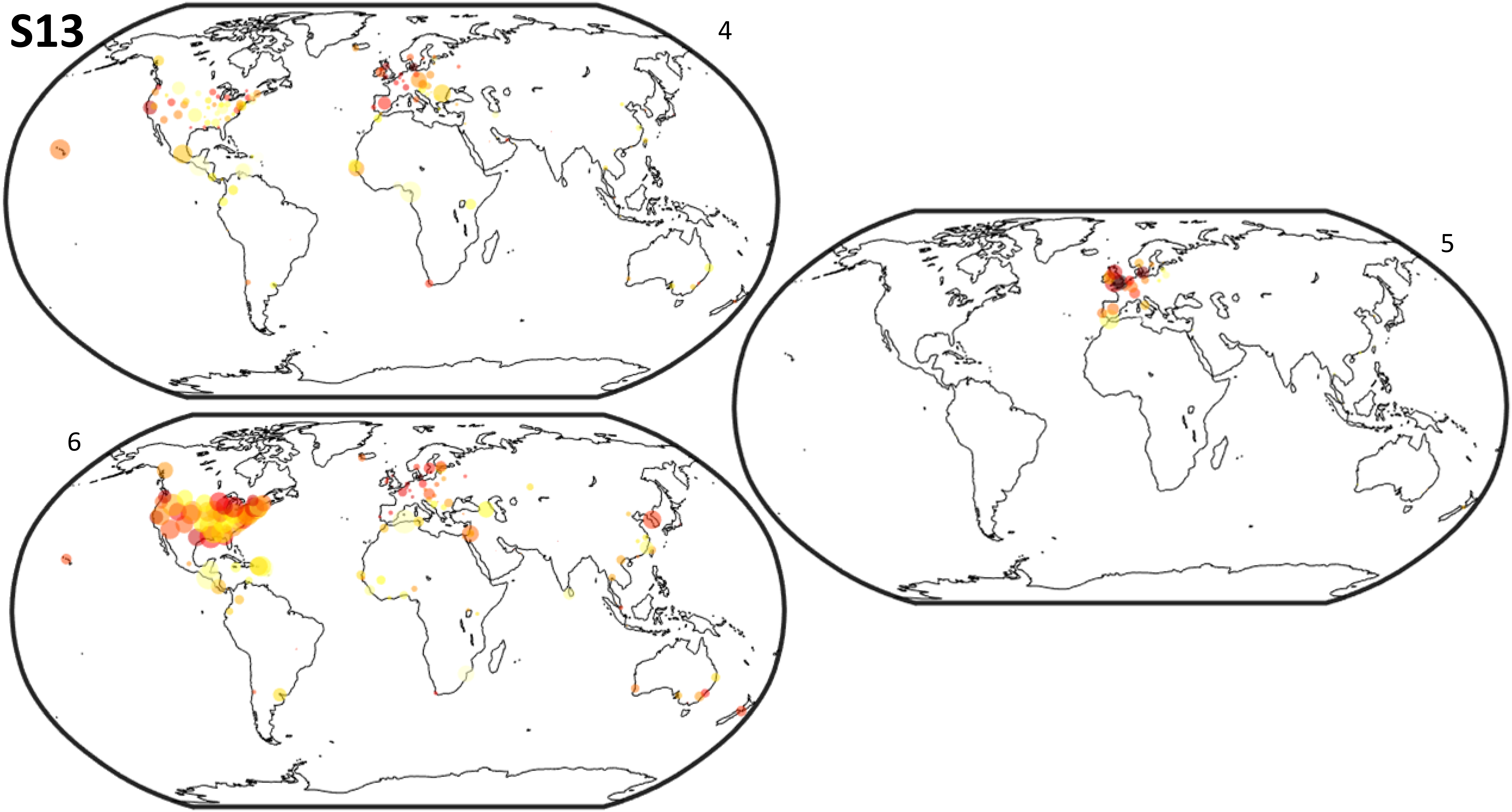

**S14**

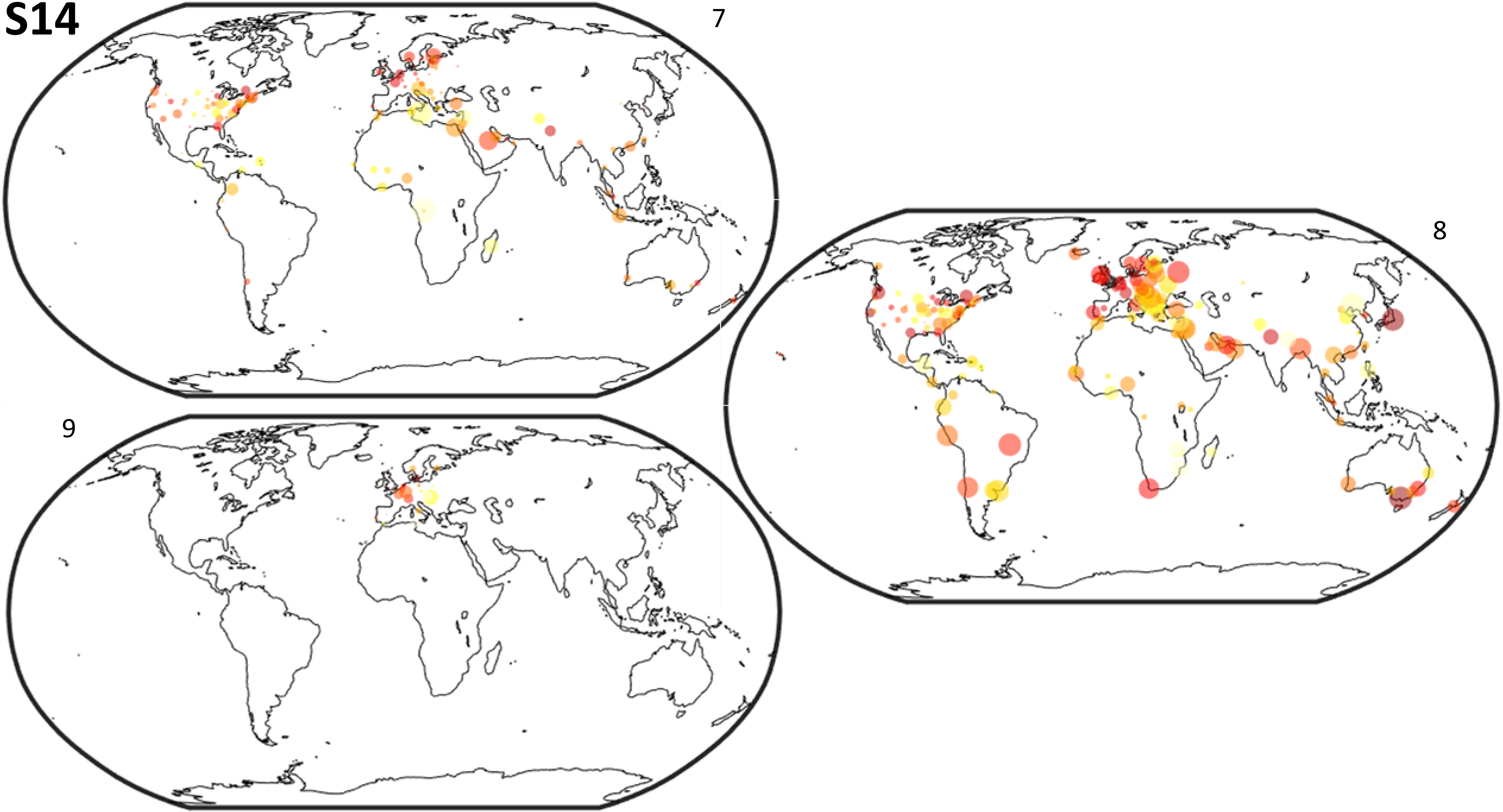

# S15

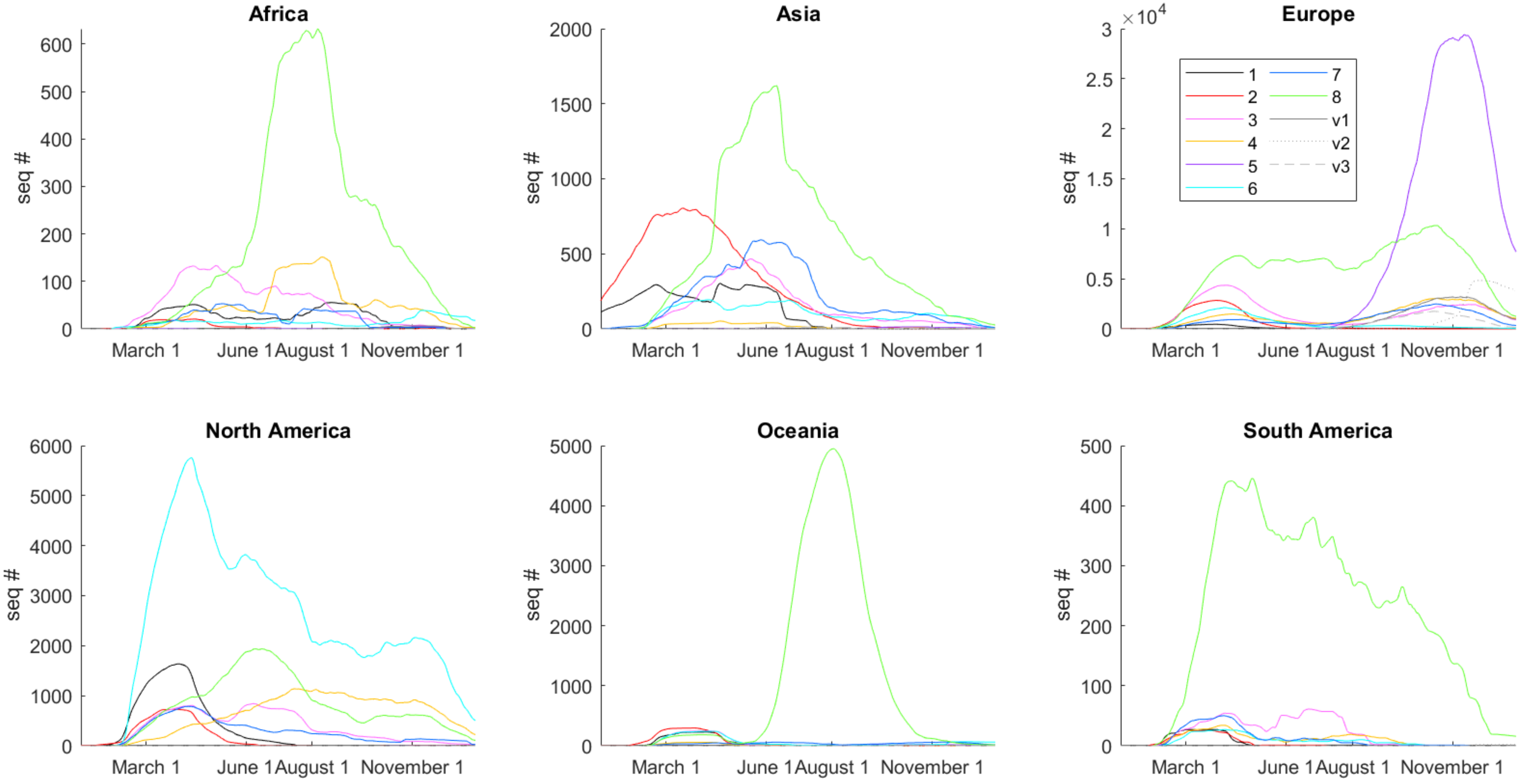

Africa
Asia
Europe
North America
Oceania
South America
seq #
$\times 10^4$
0
100
200
300
400
500
600
500
1000
1500
2000
0.5
1
1.5
2
2.5
3
3000
4000
5000
6000
March 1
June 1
August 1
November 1
1
2
3
4
5
6
7
8
v1
v2
v3

**S16**

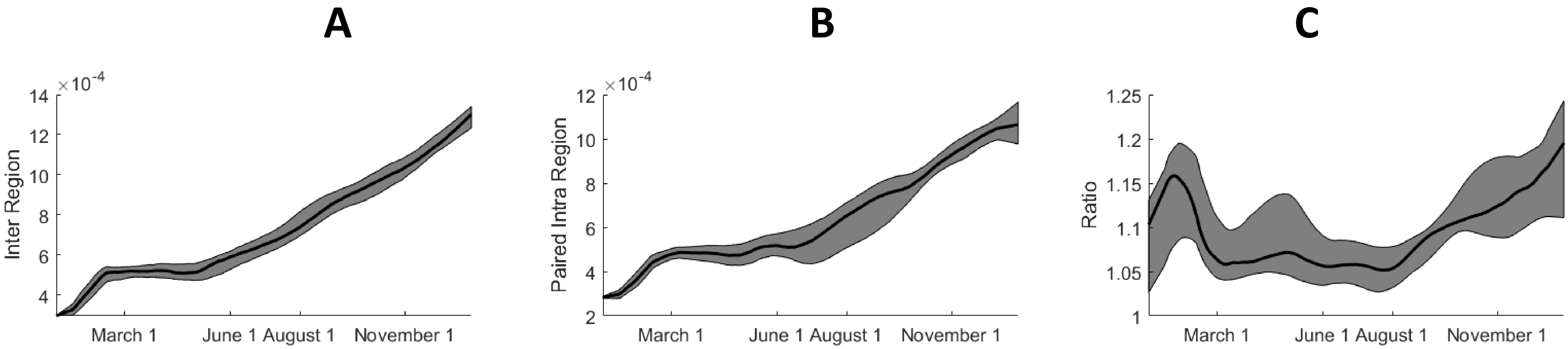

A
B
C
×10^-4
14
12
10
8
6
4
Inter Region
March 1
June 1 August 1
November 1
×10^-4
12
10
8
6
4
2
Paired Intra Region
March 1
June 1 August 1
November 1
1.25
1.2
1.15
1.1
1.05
1
Ratio
March 1
June 1 August 1
November 1

**S17**

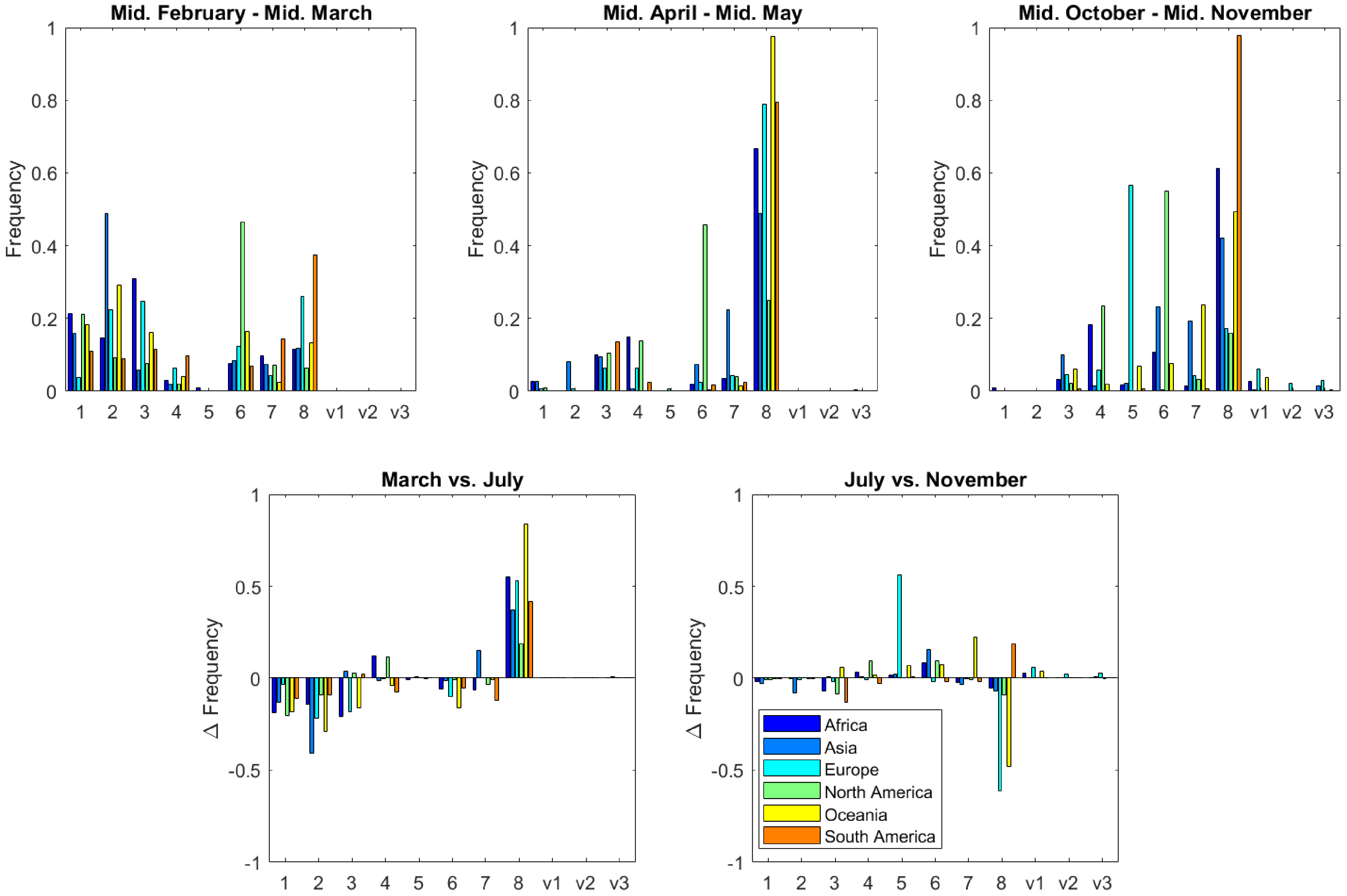
Mid. February - Mid. March
Mid. April - Mid. May
Mid. October - Mid. November
Frequency
1
0.8
0.6
0.4
0.2
0
1 2 3 4 5 6 7 8 v1 v2 v3
March vs. July
July vs. November
Δ Frequency
1
0.5
0
-0.5
-1
Africa
Asia
Europe
North America
Oceania
South America

# S18

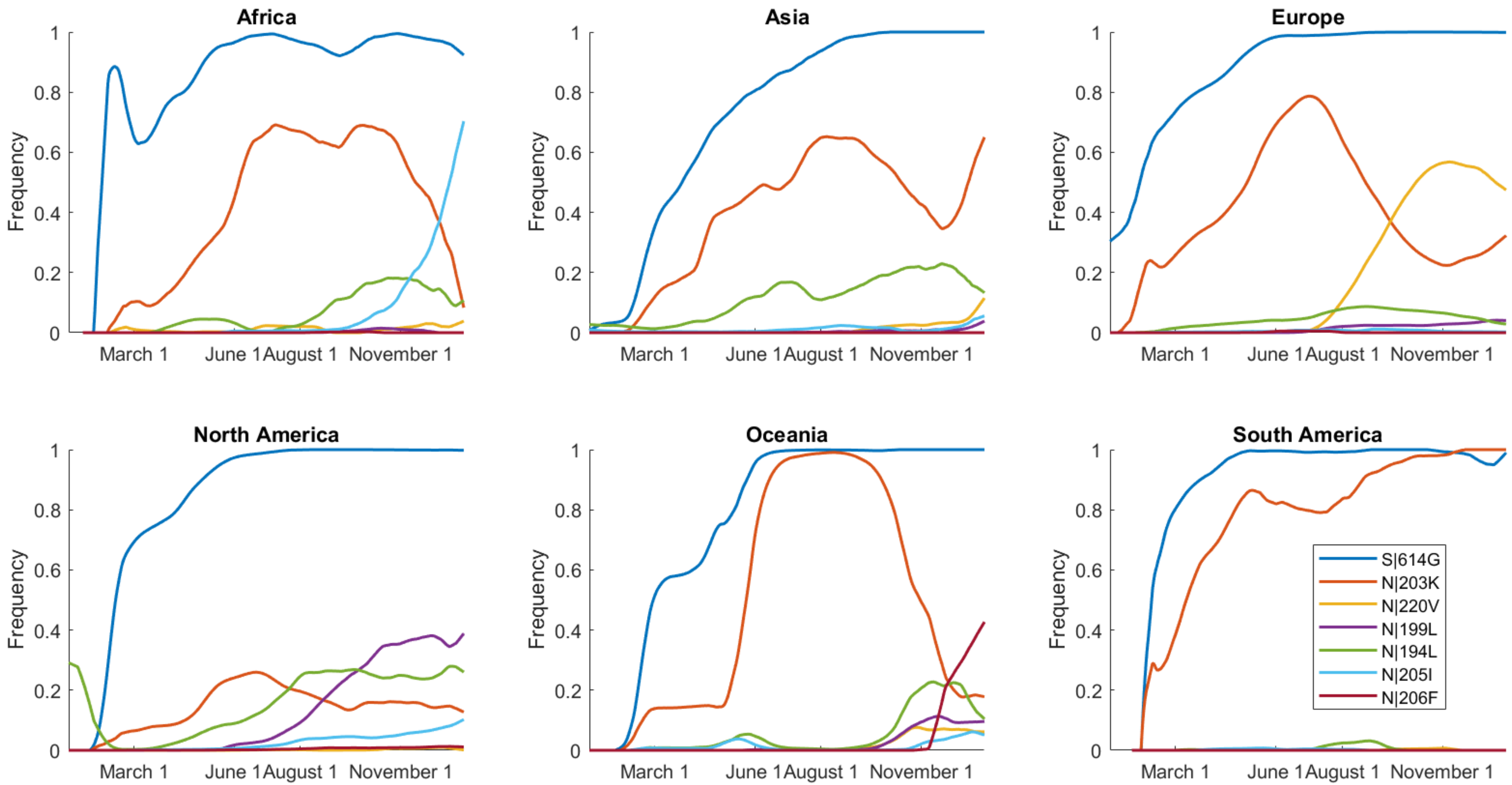

Africa
Asia
Europe
North America
Oceania
South America
Frequency
1
0.8
0.6
0.4
0.2
0
March 1
June 1
August 1
November 1
S|614G
N|203K
N|220V
N|199L
N|194L
N|205I
N|206F

# S19

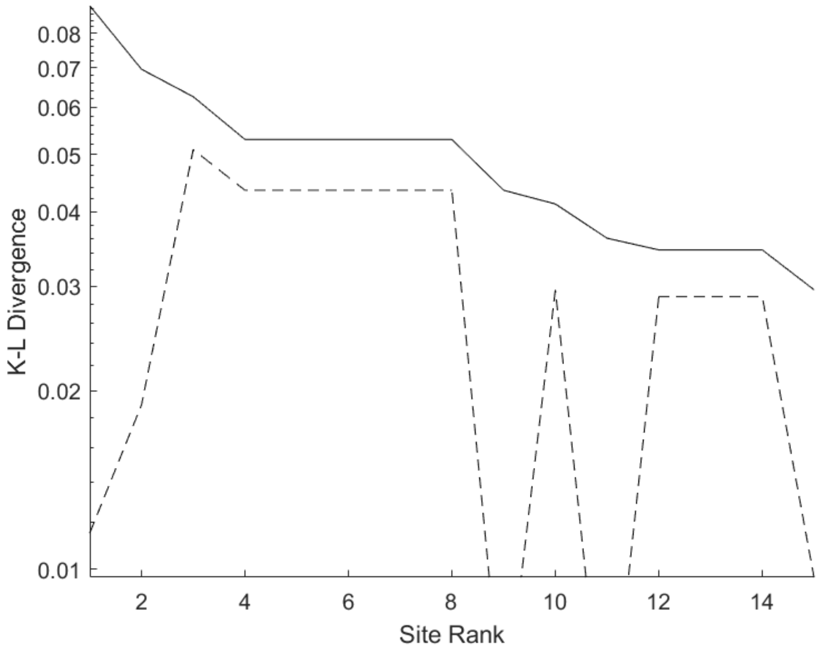


Partition 7, Late 2020, Oceania

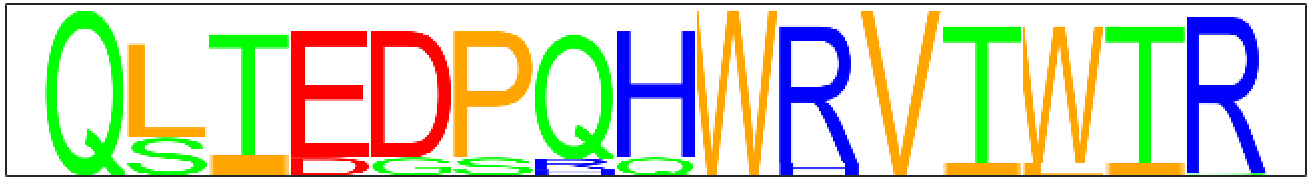

Partition 7, Oceania

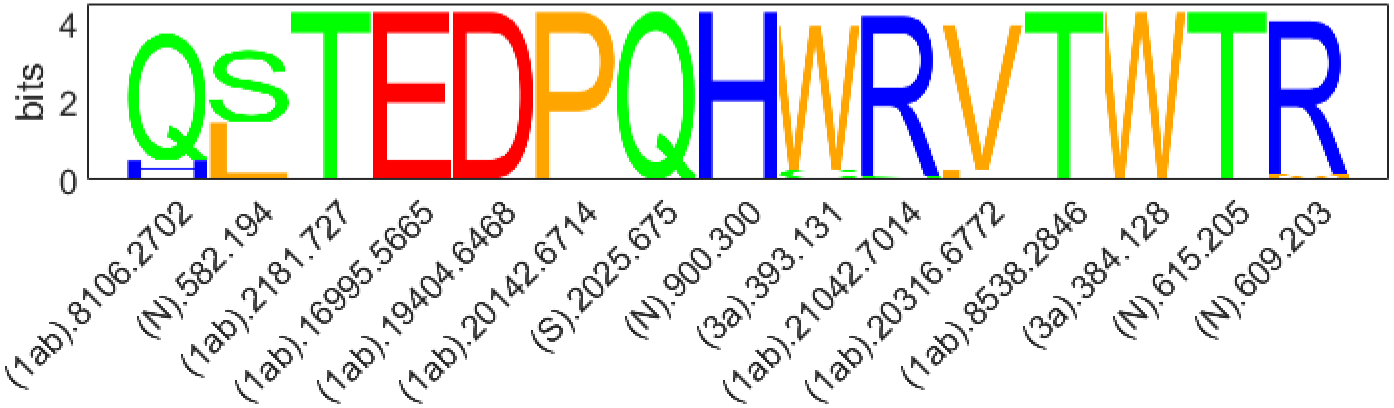

# S20

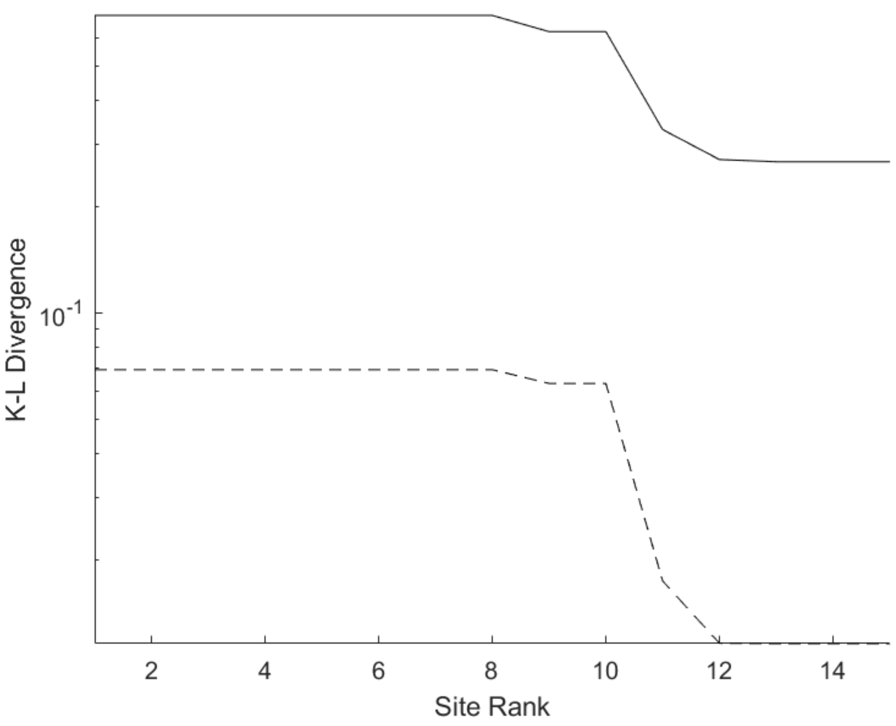


Partition 6, Late 2020, Oceania

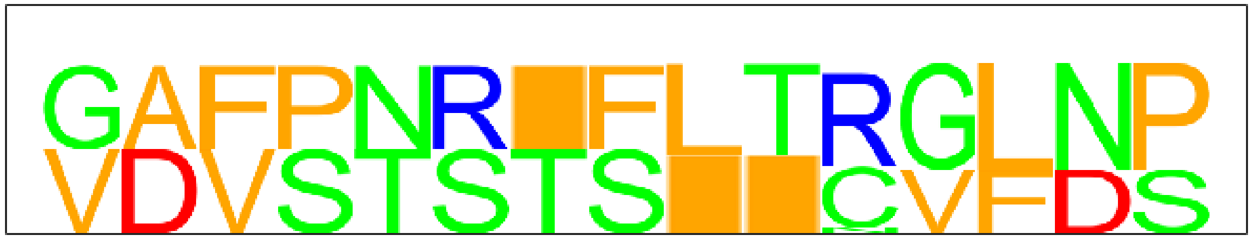

Partition 6, Oceania

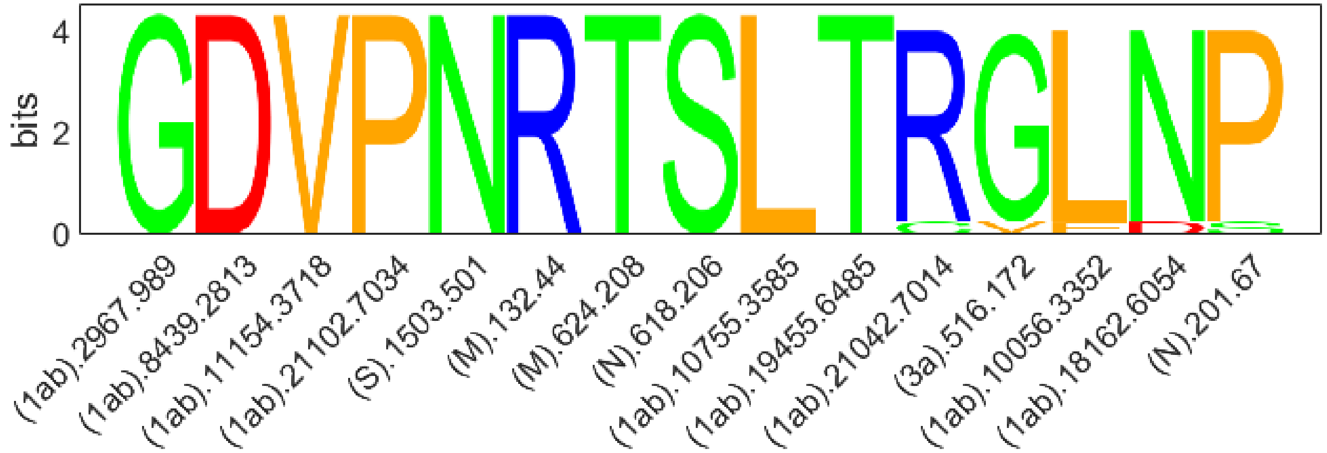

# S21

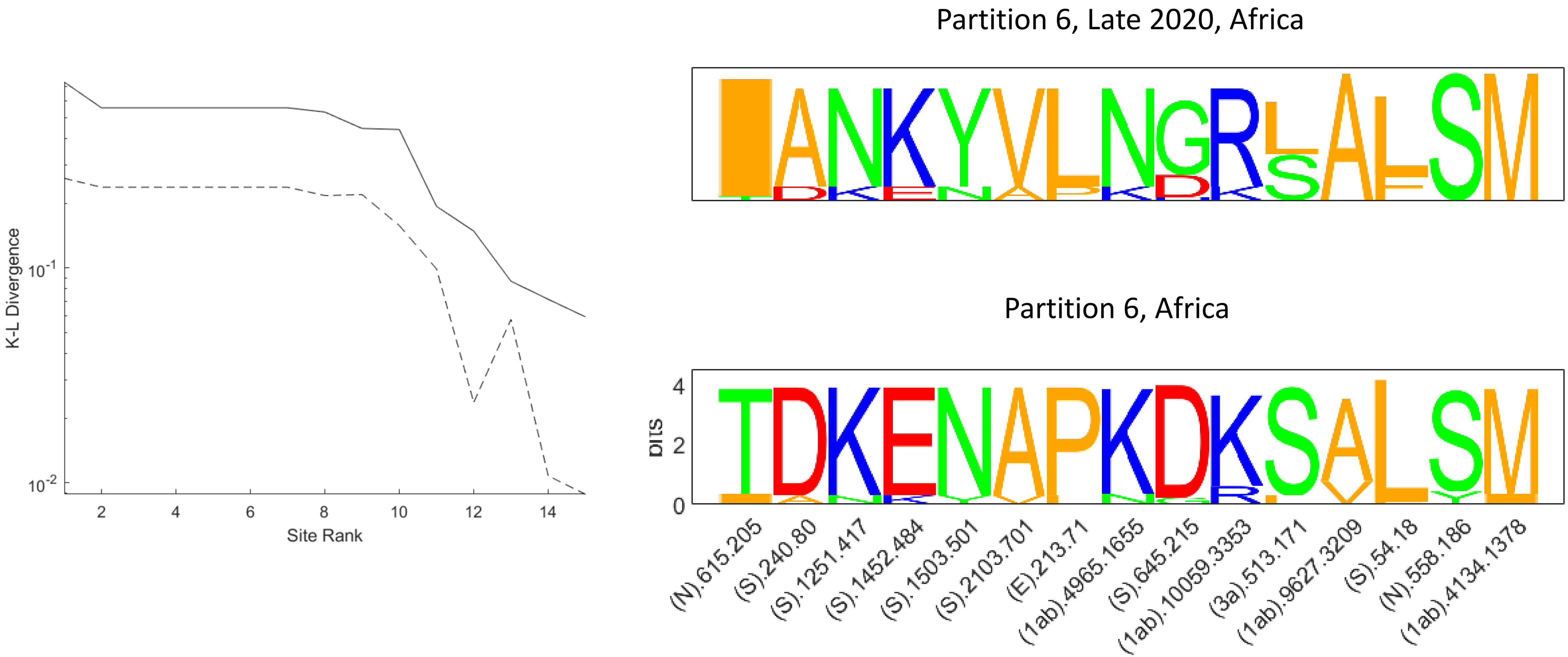
Partition 6, Late 2020, Africa
Partition 6, Africa
K-L Divergence
$10^{-1}$
$10^{-2}$
2
4
6
8
10
12
14
Site Rank
bits
4
2
0
(N).615.205
(S).240.80
(S).1251.417
(S).1452.484
(S).1503.501
(S).2103.701
(E).213.71
(1ab).4965.1655
(S).645.215
(1ab).10059.3353
(3a).513.171
(1ab).9627.3209
(S).54.18
(N).558.186
(1ab).4134.1378

# S22

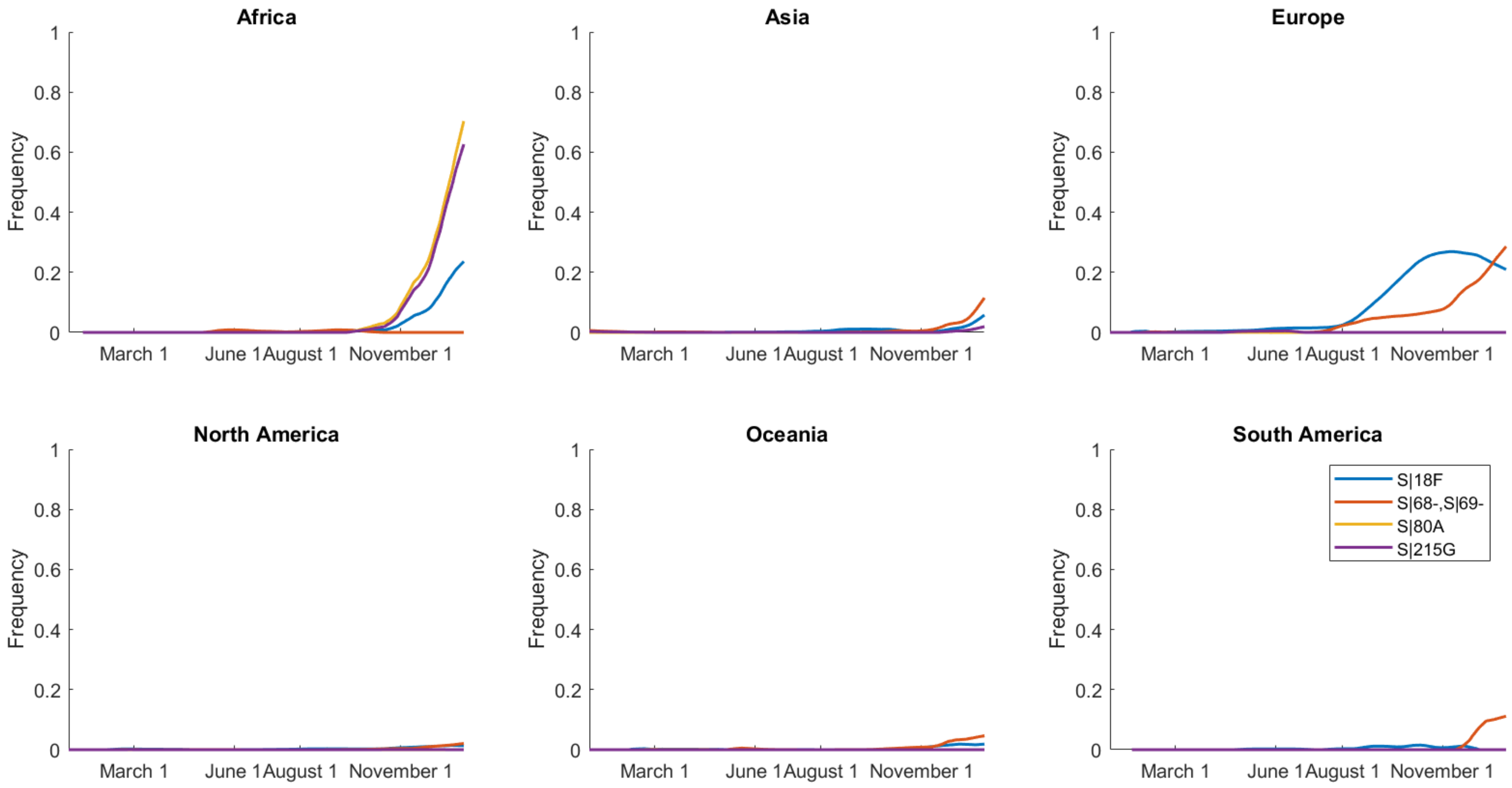
Africa
Asia
Europe
North America
Oceania
South America
Frequency
0
0.2
0.4
0.6
0.8
1
March 1
June 1
August 1
November 1
S|18F
S|68-,S|69-
S|80A
S|215G

# S23

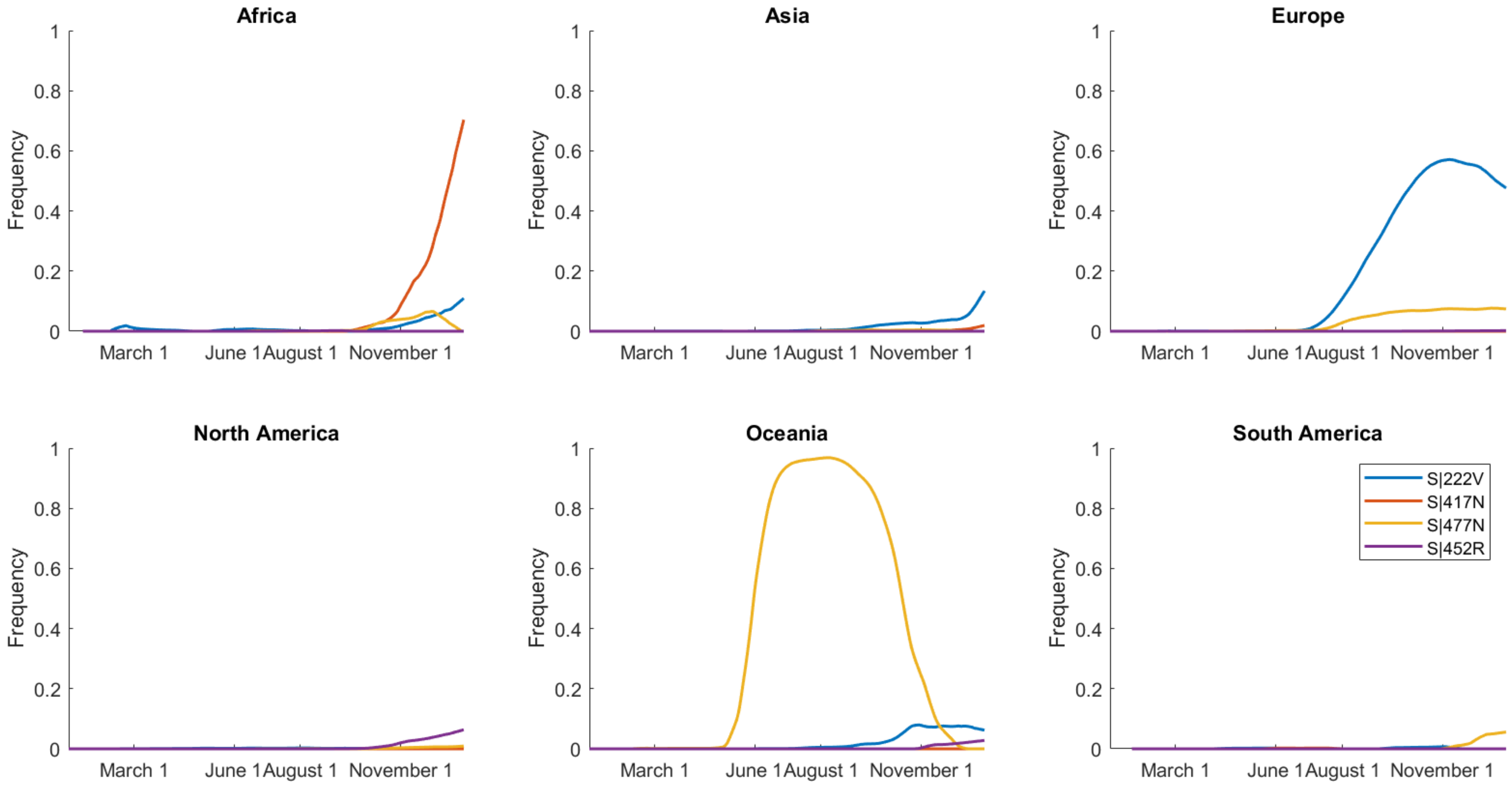
Africa
Asia
Europe
North America
Oceania
South America
Frequency
0
0.2
0.4
0.6
0.8
1
March 1
June 1
August 1
November 1
S|222V
S|417N
S|477N
S|452R

# S24

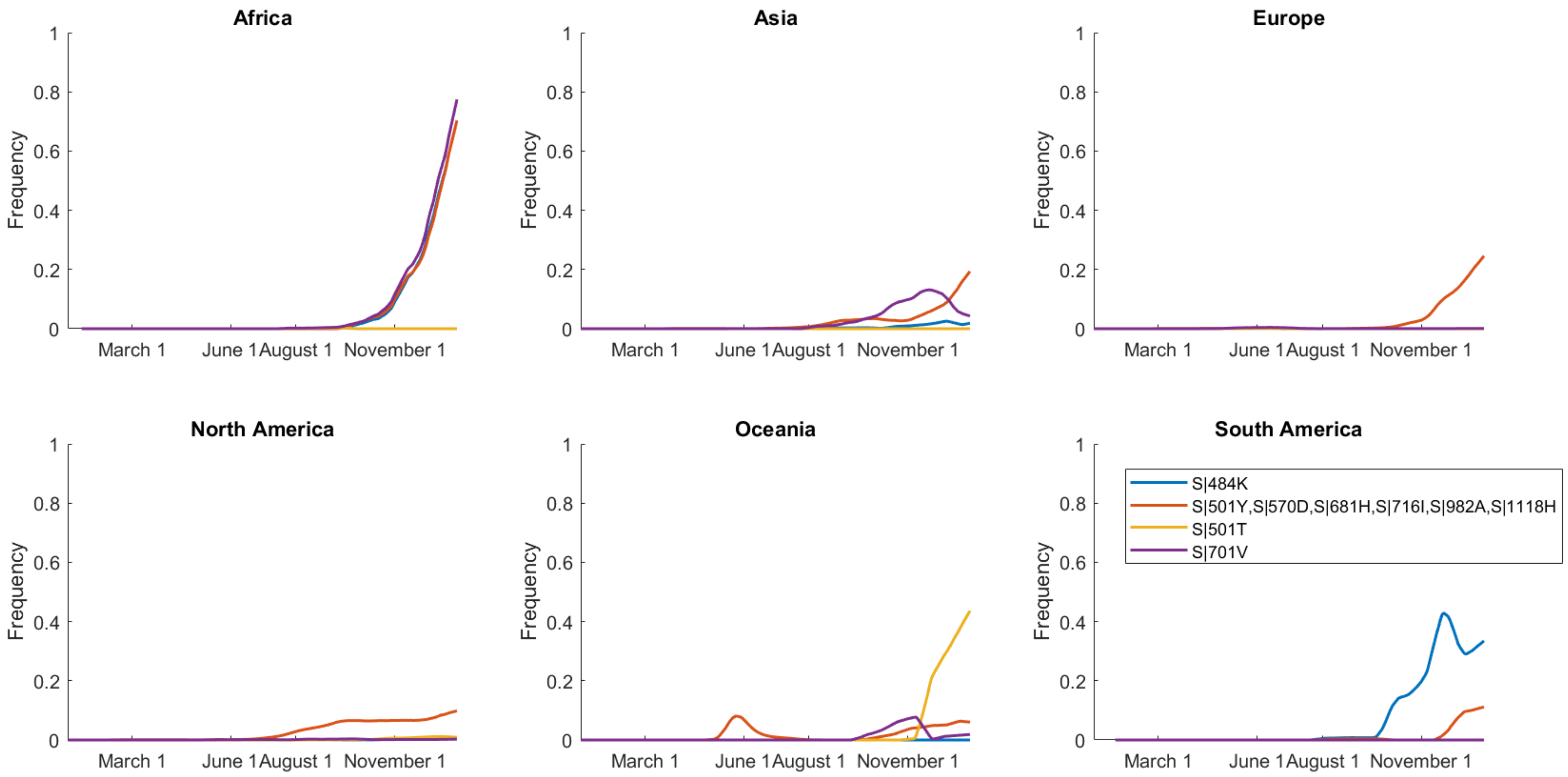
Africa
Asia
Europe
North America
Oceania
South America
Frequency
1
0.8
0.6
0.4
0.2
0
March 1
June 1
August 1
November 1
S|484K
S|501Y,S|570D,S|681H,S|716I,S|982A,S|1118H
S|501T
S|701V

# S25

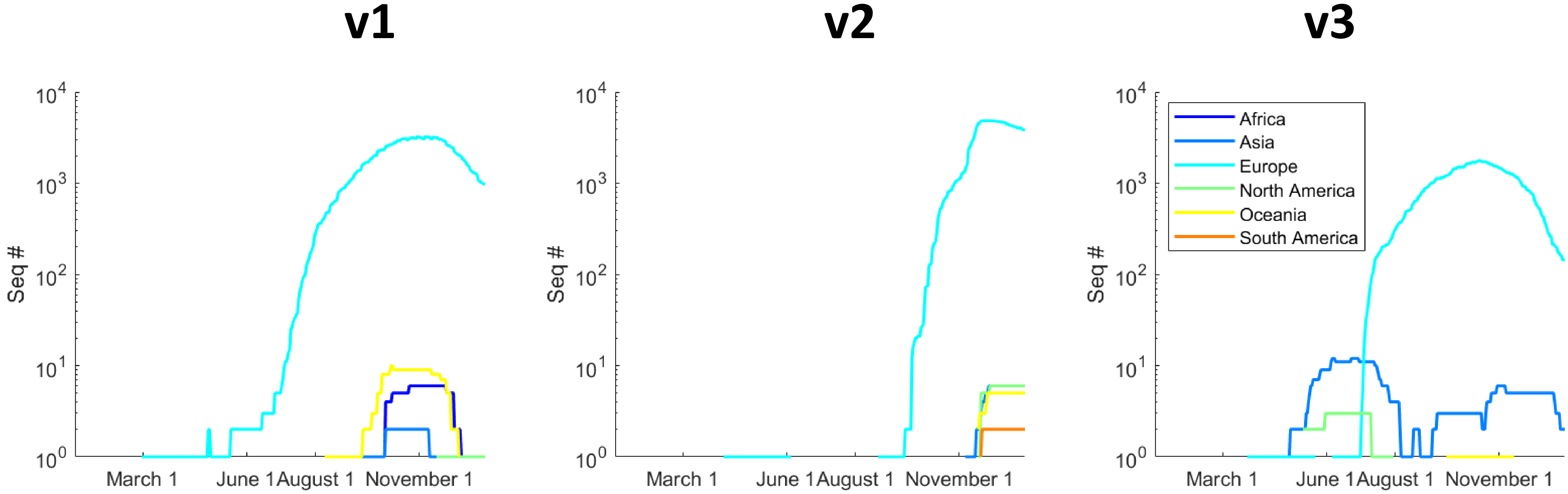
v1
v2
v3
Seq #
10^4
10^3
10^2
10^1
10^0
March 1
June 1
August 1
November 1
Africa
Asia
Europe
North America
Oceania
South America

**Table S1**

| Location | Codon1 | Codon2 | Weight | #Events |
|---|---|---|---|---|
| orf1ab, 16 | ctc(L) | ctt(L) | 0.023 | 127 |
| orf1ab, 24 | cgc(R) | tgc(C) | 0.004 | 85 |
| orf1ab, 60 | gtt(V) | gtc(V) | 0.111 | 11 |
| orf1ab, 140 | cta(L) | tta(L) | 0.003 | 87 |
| orf1ab, 186 | gtc(V) | gtt(V) | 0.002 | 98 |
| orf1ab, 265 | acc(T) | atc(I) | 0.131 | 71 |
| orf1ab, 300 | att(I) | ttt(F) | 0.012 | 50 |
| orf1ab, 309 | cca(P) | cta(L) | 0.003 | 96 |
| orf1ab, 443 | tcc(S) | ttc(F) | 0.001 | 84 |
| orf1ab, 473 | atc(I) | att(I) | 0.002 | 105 |
| orf1ab, 519 | ggt(G) | agt(S) | 0.004 | 125 |
| orf1ab, 540 | gct(A) | gtt(V) | 0.001 | 90 |
| orf1ab, 549 | tcc(S) | tct(S) | 0.004 | 141 |
| orf1ab, 717 | tac(Y) | tat(Y) | 0.017 | 20 |
| orf1ab, 924 | ttt(F) | ttc(F) | 0.005 | 89 |
| orf1ab, 924 | ttc(F) | ttt(F) | 0.784 | 20 |
| orf1ab, 944 | tca(S) | tta(L) | 0.003 | 139 |
| orf1ab,1113 | cac(H) | tac(Y) | 0.01 | 61 |
| orf1ab,1246 | act(T) | att(I) | 0.011 | 29 |
| orf1ab,1273 | gac(D) | gat(D) | 0.003 | 87 |
| orf1ab,1426 | acc(T) | act(T) | 0.013 | 67 |
| orf1ab,1627 | cta(L) | tta(L) | 0.003 | 91 |
| orf1ab,1788 | acg(T) | act(T) | 0.012 | 28 |
| orf1ab,1868 | tac(Y) | tat(Y) | 0.003 | 96 |
| orf1ab,1925 | ttc(F) | ttt(F) | 0.004 | 88 |
| orf1ab,2007 | acc(T) | act(T) | 0.111 | 46 |
| orf1ab,2016 | aca(T) | aaa(K) | 0.012 | 13 |

| | | | | |
|---|---|---|---|---|
| orf1ab,2124 | act(T) | att(I) | 0.002 | 96 |
| orf1ab,2221 | ctt(L) | cct(P) | 0.015 | 6 |
| orf1ab,2226 | ctg(L) | ttg(L) | 0.009 | 25 |
| orf1ab,2260 | ctt(L) | cct(P) | 0.041 | 3 |
| orf1ab,2385 | atc(I) | att(I) | 0.002 | 85 |
| orf1ab,2421 | gtc(V) | gtt(V) | 0.005 | 96 |
| orf1ab,2501 | atc(I) | acc(T) | 0.012 | 11 |
| orf1ab,2594 | tac(Y) | tat(Y) | 0.011 | 14 |
| orf1ab,2839 | agt(S) | agc(S) | 0.893 | 21 |
| orf1ab,2884 | ttc(F) | ttt(F) | 0.004 | 87 |
| orf1ab,3055 | atc(I) | att(I) | 0.006 | 148 |
| orf1ab,3076 | cat(H) | tat(Y) | 0.001 | 91 |
| orf1ab,3087 | atg(M) | att(I) | 0.012 | 32 |
| orf1ab,3143 | gtt(V) | gct(A) | 0.072 | 6 |
| orf1ab,3278 | ggt(G) | agt(S) | 0.016 | 13 |
| orf1ab,3291 | aac(N) | aat(N) | 0.002 | 101 |
| orf1ab,3352 | ctt(L) | ttt(F) | 0.017 | 48 |
| orf1ab,3353 | aag(K) | agg(R) | 0.01 | 226 |
| orf1ab,3368 | cgc(R) | cgt(R) | 0.004 | 121 |
| orf1ab,3535 | ctg(L) | ctt(L) | 0.008 | 89 |
| orf1ab,3603 | ttc(F) | ttt(F) | 0.004 | 149 |
| orf1ab,3606 | ttt(F) | ttg(L) | 0.008 | 107 |
| orf1ab,3606 | ttg(L) | ttt(F) | 0.077 | 802 |
| orf1ab,3655 | atg(M) | att(I) | 0.013 | 32 |
| orf1ab,3690 | gtg(V) | gtt(V) | 0.001 | 86 |
| orf1ab,3718 | gtt(V) | ttt(F) | 0.005 | 129 |
| orf1ab,3744 | tac(Y) | tat(Y) | 0.011 | 18 |
| orf1ab,3796 | ctc(L) | ctt(L) | 0.001 | 94 |
| orf1ab,3884 | tca(S) | tta(L) | 0.013 | 55 |

| | | | | |
|---|---|---|---|---|
| orf1ab,4058 | ccc(P) | cct(P) | 0.002 | 88 |
| orf1ab,4065 | aca(T) | ata(I) | 0.001 | 95 |
| orf1ab,4083 | acg(T) | atg(M) | 0.002 | 85 |
| orf1ab,4317 | gag(E) | gat(D) | 0.01 | 4 |
| orf1ab,4424 | tac(Y) | tat(Y) | 0.012 | 66 |
| orf1ab,4489 | gct(A) | gtt(V) | 0.014 | 46 |
| orf1ab,4577 | gct(A) | tct(S) | 0.011 | 18 |
| orf1ab,4619 | cca(P) | cta(L) | 0.002 | 85 |
| orf1ab,4715 | cct(P) | ctt(L) | 0.785 | 26 |
| orf1ab,4779 | cta(L) | tta(L) | 0.002 | 91 |
| orf1ab,4820 | ttc(F) | ttt(F) | 0.003 | 121 |
| orf1ab,4847 | tac(Y) | tat(Y) | 0.03 | 74 |
| orf1ab,5020 | aac(N) | aat(N) | 0.034 | 91 |
| orf1ab,5152 | gac(D) | gat(D) | 0.003 | 102 |
| orf1ab,5158 | ttc(F) | ttt(F) | 0.003 | 104 |
| orf1ab,5168 | gtg(V) | ttg(L) | 0.011 | 17 |
| orf1ab,5214 | cag(Q) | cat(H) | 0.002 | 85 |
| orf1ab,5541 | tac(Y) | tat(Y) | 0.007 | 257 |
| orf1ab,5542 | aaa(K) | aga(R) | 0.011 | 4 |
| orf1ab,5585 | gag(E) | gat(D) | 0.012 | 45 |
| orf1ab,5614 | cat(H) | tat(Y) | 0.014 | 66 |
| orf1ab,5680 | gtc(V) | gtt(V) | 0.001 | 93 |
| orf1ab,5762 | ctc(L) | ctt(L) | 0.002 | 90 |
| orf1ab,5828 | ctt(L) | cct(P) | 0.94 | 6 |
| orf1ab,5865 | tgt(C) | tat(Y) | 0.939 | 1 |
| orf1ab,5932 | ctt(L) | ctc(L) | 0.937 | 5 |
| orf1ab,6054 | aat(N) | gat(D) | 0.011 | 11 |
| orf1ab,6097 | gac(D) | gat(D) | 0.008 | 107 |
| orf1ab,6205 | cta(L) | tta(L) | 0.048 | 77 |

| | | | | |
|---|---|---|---|---|
| orf1ab,6302 | ttc(F) | ttt(F) | 0.003 | 89 |
| orf1ab,6335 | aac(N) | aat(N) | 0.002 | 91 |
| orf1ab,6420 | ctc(L) | ctt(L) | 0.01 | 131 |
| orf1ab,6525 | aat(N) | aac(N) | 0.013 | 58 |
| orf1ab,6541 | aag(K) | aaa(K) | 0.893 | 1 |
| orf1ab,6573 | gtc(V) | gtt(V) | 0.002 | 95 |
| orf1ab,6579 | gtt(V) | ttt(F) | 0.001 | 86 |
| orf1ab,6638 | gtc(V) | gtt(V) | 0.003 | 108 |
| orf1ab,6668 | tta(L) | ttg(L) | 0.085 | 24 |
| orf1ab,6958 | aag(K) | agg(R) | 0.005 | 197 |
| orf1ab,6997 | gcg(A) | gcc(A) | 0.112 | 40 |
| orf1ab,7014 | cgc(R) | tgc(C) | 0.011 | 30 |
| S , 5 ctt(L) | ttt(F) | 0.016 | 522 | |
| S , 18 | ctt(L) | ttt(F) | 0.046 | 111 |
| S , 22 | act(T) | att(I) | 0.002 | 85 |
| S , 49 | cat(H) | tat(Y) | 0.003 | 89 |
| S , 54 | ttg(L) | ttt(F) | 0.003 | 95 |
| S , 95 | act(T) | att(I) | 0.003 | 105 |
| S , 98 | tct(S) | ttt(F) | 0.011 | 78 |
| S , 222 | gct(A) | gtt(V) | 0.114 | 81 |
| S , 294 | gac(D) | gat(D) | 0.014 | 46 |
| S , 439 | aac(N) | aaa(K) | 0.012 | 11 |
| S , 475 | gcc(A) | gct(A) | 0.002 | 86 |
| S , 477 | agc(S) | aac(N) | 0.013 | 41 |
| S , 543 | ttc(F) | ttt(F) | 0.003 | 92 |
| S , 614 | gat(D) | ggt(G) | 0.788 | 38 |
| S , 614 | ggt(G) | gat(D) | 0.021 | 57 |
| S , 677 | cag(Q) | cat(H) | 0.003 | 103 |
| S , 723 | acc(T) | act(T) | 0.016 | 6 |

| | | | | |
|---|---|---|---|---|
| S , 769 | gga(G) | gta(V) | 0.003 | 88 |
| S , 789 | tac(Y) | tat(Y) | 0.012 | 53 |
| S , 821 | cta(L) | tta(L) | 0.002 | 89 |
| S , 856 | aac(N) | aat(N) | 0.002 | 88 |
| S , 924 | gcc(A) | gct(A) | 0.016 | 31 |
| S , 939 | tct(S) | ttt(F) | 0.002 | 93 |
| S ,1044 | gga(G) | ggt(G) | 0.009 | 8 |
| orf3a , 13 | gta(V) | tta(L) | 0.01 | 57 |
| orf3a , 38 | caa(Q) | cga(R) | 0.009 | 9 |
| orf3a , 43 | ttc(F) | ttt(F) | 0.003 | 115 |
| orf3a , 57 | cag(Q) | cat(H) | 0.203 | 70 |
| orf3a , 64 | atc(I) | acc(T) | 0.041 | 3 |
| orf3a , 78 | cac(H) | tac(Y) | 0.002 | 86 |
| orf3a , 106 | ctc(L) | ttc(F) | 0.001 | 86 |
| orf3a , 106 | ctc(L) | ctt(L) | 0.013 | 73 |
| orf3a , 131 | tgg(W) | tgt(C) | 0.003 | 97 |
| orf3a , 151 | act(T) | att(I) | 0.002 | 97 |
| orf3a , 155 | gac(D) | tac(Y) | 0.002 | 87 |
| orf3a , 171 | tca(S) | tta(L) | 0.004 | 126 |
| orf3a , 172 | ggt(G) | cgt(R) | 0.009 | 12 |
| orf3a , 172 | ggt(G) | gtt(V) | 0.012 | 37 |
| orf3a , 175 | aca(T) | ata(I) | 0.004 | 93 |
| orf3a , 202 | gta(V) | tta(L) | 0.01 | 52 |
| orf3a , 232 | att(I) | atc(I) | 0.919 | 7 |
| orf3a , 251 | ggt(G) | gtt(V) | 0.023 | 13 |
| E , 4 ttc(F) | ttt(F) | 0.001 | 86 | |
| E , 73 | ctt(L) | ttt(F) | 0.002 | 93 |
| M , 3 | gat(D) | ggt(G) | 0.009 | 23 |
| M , 41 | aac(N) | aat(N) | 0.002 | 86 |

| | | | | | |
|---|---|---|---|---|---|
| M | , 53 | ttc(F) | ttt(F) | 0.006 | 175 |
| M | , 71 | tac(Y) | tat(Y) | 0.035 | 107 |
| M | , 93 | ctc(L) | ctt(L) | 0.003 | 96 |
| M | , 93 | ctc(L) | ctg(L) | 0.111 | 8 |
| M | , 117 | aac(N) | aat(N) | 0.001 | 98 |
| M | , 118 | att(I) | atc(I) | 0.011 | 11 |
| M | , 125 | cat(H) | tat(Y) | 0.003 | 97 |
| orf6 | , 4 | ctc(L) | ctt(L) | 0.003 | 103 |
| orf6 | , 32 | atc(I) | att(I) | 0.001 | 90 |
| orf6 | , 61 | gat(D) | gac(D) | 0.006 | 169 |
| orf7b | , 15 | gcc(A) | gca(A) | 0.009 | 6 |
| orf8 | , 17 | cat(H) | cac(H) | 0.039 | 15 |
| orf8 | , 17 | cac(H) | cat(H) | 0.073 | 49 |
| orf8 | , 24 | tca(S) | tta(L) | 0.027 | 38 |
| orf8 | , 62 | gtg(V) | ttg(L) | 0.005 | 157 |
| orf8 | , 65 | gct(A) | gtt(V) | 0.005 | 122 |
| orf8 | , 67 | tct(S) | ttt(F) | 0.003 | 138 |
| orf8 | , 84 | tca(S) | tta(L) | 0.891 | 8 |
| orf8 | , 120 | ttc(F) | ttt(F) | 0.007 | 236 |
| N | , 13 | ccc(P) | ctc(L) | 0.015 | 78 |
| N | , 35 | gcg(A) | gct(A) | 0.004 | 101 |
| N | , 67 | cct(P) | tct(S) | 0.011 | 30 |
| N | , 110 | ttc(F) | ttt(F) | 0.003 | 118 |
| N | , 126 | aac(N) | aat(N) | 0.01 | 40 |
| N | , 128 | gac(D) | gat(D) | 0.009 | 89 |
| N | , 139 | ttt(F) | ttg(L) | 0.085 | 2 |
| N | , 192 | aac(N) | aat(N) | 0.002 | 88 |
| N | , 193 | agt(S) | att(I) | 0.004 | 88 |
| N | , 194 | tca(S) | tta(L) | 0.065 | 131 |

| | | | | | |
|---|---|---|---|---|---|
| N | , 199 | cca(P) | cta(L) | 0.021 | 35 |
| N | , 202 | agt(S) | aat(N) | 0.018 | 14 |
| N | , 203 | agg(R) | aag(K) | 0.254 | 29 |
| N | , 203 | aag(K) | aaa(K) | 0.252 | 1 |
| N | , 204 | gga(G) | cga(R) | 0.251 | 10 |
| N | , 205 | act(T) | att(I) | 0.009 | 123 |
| N | , 220 | gct(A) | gtt(V) | 0.112 | 21 |
| N | , 234 | atg(M) | att(I) | 0.006 | 93 |
| N | , 234 | atg(M) | atc(I) | 0.011 | 10 |
| N | , 274 | ttt(F) | ttc(F) | 0.886 | 6 |
| N | , 274 | ttc(F) | ttt(F) | 0.009 | 159 |
| N | , 327 | tcg(S) | ttg(L) | 0.001 | 89 |
| N | , 365 | cca(P) | tca(S) | 0.01 | 37 |
| N | , 376 | gct(A) | act(T) | 0.011 | 8 |
| N | , 377 | gat(D) | tat(Y) | 0.007 | 148 |

**Table S2**

List 1, unrestricted by single substitution weight

| Location | Codon1 | Codon2 | Weight | #Events |
|---|---|---|---|---|
| S , 614 | gat(D) | ggt(G) | 0.788 | 38 |
| N , 203 | agg(R) | aag(K) | 0.254 | 29 |
| N , 204 | gga(G) | cga(R) | 0.251 | 10 |
| orf3a , 57 | cag(Q) | cat(H) | 0.203 | 70 |
| orf1ab,3606 | ttg(L) | ttt(F) | 0.077 | 802 |
| orf3a , 251 | ggt(G) | gtt(V) | 0.023 | 13 |
| S , 614 | ggt(G) | gat(D) | 0.021 | 57 |
| N , 202 | agt(S) | aat(N) | 0.018 | 14 |
| orf1ab,3278 | ggt(G) | agt(S) | 0.016 | 13 |
| S , 477 | agc(S) | aac(N) | 0.013 | 41 |
| orf1ab,3655 | atg(M) | att(I) | 0.013 | 32 |
| orf1ab, 300 | att(I) | ttt(F) | 0.012 | 50 |
| orf1ab,5585 | gag(E) | gat(D) | 0.012 | 45 |
| orf1ab,3087 | atg(M) | att(I) | 0.012 | 32 |
| orf3a , 172 | ggt(G) | gtt(V) | 0.012 | 37 |
| orf1ab,2501 | atc(I) | acc(T) | 0.012 | 11 |
| orf1ab,5168 | gtg(V) | ttg(L) | 0.011 | 17 |
| orf1ab,4577 | gct(A) | tct(S) | 0.011 | 18 |
| orf1ab,6054 | aat(N) | gat(D) | 0.011 | 11 |
| N , 376 | gct(A) | act(T) | 0.011 | 8 |
| N , 234 | atg(M) | atc(I) | 0.011 | 10 |
| orf3a , 13 | gta(V) | tta(L) | 0.01 | 57 |
| orf3a , 202 | gta(V) | tta(L) | 0.01 | 52 |
| orf1ab,3353 | aag(K) | agg(R) | 0.01 | 226 |
| orf3a , 172 | ggt(G) | cgt(R) | 0.009 | 12 |

| | | | | |
|---|---|---|---|---|
| orf3a , 38 | caa(Q) | cga(R) | 0.009 | 9 |
| M , 3 | gat(D) | ggt(G) | 0.009 | 23 |
| orf1ab,3606 | ttt(F) | ttg(L) | 0.008 | 107 |
| N , 194 | tta(L) | tca(S) | 0.008 | 29 |
| S , 262 | gct(A) | tct(S) | 0.008 | 38 |
| orf8 , 92 | gaa(E) | aaa(K) | 0.007 | 9 |
| orf1ab,5922 | gca(A) | tca(S) | 0.007 | 21 |
| N , 377 | gat(D) | tat(Y) | 0.007 | 148 |
| orf1ab,4241 | atg(M) | att(I) | 0.007 | 10 |
| orf1ab,2702 | cag(Q) | cat(H) | 0.007 | 27 |
| N , 292 | atc(I) | acc(T) | 0.007 | 13 |
| orf6 , 33 | ata(I) | aca(T) | 0.006 | 12 |
| orf3a , 75 | aag(K) | aat(N) | 0.006 | 26 |
| orf1ab,5048 | gct(A) | tct(S) | 0.006 | 40 |
| N , 234 | atg(M) | att(I) | 0.006 | 93 |
| orf1ab,3334 | ggt(G) | agt(S) | 0.006 | 23 |
| orf8 , 62 | gtg(V) | ctg(L) | 0.006 | 18 |
| orf3a , 196 | gga(G) | gta(V) | 0.006 | 8 |
| orf8 , 52 | aga(R) | ata(I) | 0.005 | 31 |
| S , 501 | aat(N) | tat(Y) | 0.005 | 8 |
| orf1ab,6958 | aag(K) | agg(R) | 0.005 | 197 |
| orf1ab,3934 | atg(M) | att(I) | 0.005 | 37 |
| orf8 , 62 | gtg(V) | ttg(L) | 0.005 | 157 |
| N , 103 | gat(D) | tat(Y) | 0.005 | 25 |
| orf1ab,3718 | gtt(V) | ttt(F) | 0.005 | 129 |
| orf1ab,2606 | atg(M) | ata(I) | 0.005 | 9 |
| S ,1176 | gtt(V) | ttt(F) | 0.005 | 52 |
| orf1ab,3839 | aaa(K) | aga(R) | 0.004 | 9 |
| orf1ab, 519 | ggt(G) | agt(S) | 0.004 | 125 |

| | | | | |
|---|---|---|---|---|
| orf1ab,1202 | aag(K) | aat(N) | 0.004 | 58 |
| orf1ab, 315 | atg(M) | att(I) | 0.004 | 9 |
| orf1ab,2873 | ata(I) | aca(T) | 0.004 | 20 |
| orf1ab,3353 | agg(R) | aag(K) | 0.004 | 9 |
| N , 193 | agt(S) | att(I) | 0.004 | 88 |
| orf1ab,3712 | atg(M) | att(I) | 0.003 | 12 |
| S ,1163 | gat(D) | tat(Y) | 0.003 | 54 |
| N , 209 | aga(R) | ata(I) | 0.003 | 70 |
| orf1ab,4653 | tta(L) | tgc(C) | 0.003 | 39 |
| orf1ab,6426 | atg(M) | att(I) | 0.003 | 34 |
| orf1ab, 379 | gga(G) | gaa(E) | 0.003 | 29 |
| S , 54 | ttg(L) | ttt(F) | 0.003 | 95 |
| S , 153 | atg(M) | acg(T) | 0.003 | 45 |
| S , 677 | cag(Q) | cat(H) | 0.003 | 103 |
| S , 583 | gag(E) | gat(D) | 0.003 | 73 |
| S , 583 | gag(E) | gac(D) | 0.003 | 13 |
| orf1ab,4080 | tat(Y) | cat(H) | 0.003 | 25 |
| S , 21 | aga(R) | ata(I) | 0.003 | 49 |
| orf1ab,1247 | aag(K) | aat(N) | 0.003 | 16 |
| S , 80 | gat(D) | tat(Y) | 0.003 | 59 |
| orf3a , 131 | tgg(W) | tgt(C) | 0.003 | 97 |
| S , 769 | gga(G) | gta(V) | 0.003 | 88 |
| orf1ab,1655 | aag(K) | aat(N) | 0.002 | 47 |
| orf1ab,5058 | atg(M) | att(I) | 0.002 | 29 |
| orf1ab,2796 | atg(M) | att(I) | 0.002 | 19 |
| orf1ab, 190 | ttc(F) | ctc(L) | 0.002 | 13 |
| orf1ab,5568 | gag(E) | gat(D) | 0.002 | 18 |
| S ,1078 | gct(A) | tct(S) | 0.002 | 84 |
| orf3a , 224 | ggt(G) | tgt(C) | 0.002 | 82 |

| | | | | |
|---|---|---|---|---|
| orf1ab,5784 | aag(K) | agg(R) | 0.002 | 26 |
| orf8 , 65 | gct(A) | tct(S) | 0.002 | 67 |
| N , 180 | agt(S) | att(I) | 0.002 | 60 |
| N , 190 | agt(S) | att(I) | 0.002 | 11 |
| orf1ab,4715 | ctt(L) | cct(P) | 0.002 | 67 |
| orf1ab, 673 | gag(E) | aag(K) | 0.002 | 10 |
| orf1ab,4646 | gag(E) | gat(D) | 0.002 | 9 |
| orf3a , 122 | aga(R) | ata(I) | 0.002 | 15 |
| S ,1167 | ggt(G) | gtt(V) | 0.002 | 14 |
| orf8 , 84 | tta(L) | tca(S) | 0.002 | 23 |
| orf1ab,5620 | gct(A) | tct(S) | 0.002 | 62 |
| S , 839 | gat(D) | tat(Y) | 0.002 | 33 |
| orf1ab,6826 | caa(Q) | cac(H) | 0.002 | 18 |

List 2, restricted by single substitution weight

| | | | | |
|---|---|---|---|---|
| S , 614 | gat(D) | ggt(G) | 0.788 | 38 |
| N , 203 | agg(R) | aag(K) | 0.254 | 29 |
| N , 204 | gga(G) | cga(R) | 0.251 | 10 |
| orf3a , 57 | cag(Q) | cat(H) | 0.203 | 70 |
| orf3a , 251 | ggt(G) | gtt(V) | 0.023 | 13 |
| N , 202 | agt(S) | aat(N) | 0.018 | 14 |
| orf1ab,3278 | ggt(G) | agt(S) | 0.016 | 13 |
| orf1ab,2501 | atc(I) | acc(T) | 0.012 | 11 |
| orf1ab,5168 | gtg(V) | ttg(L) | 0.011 | 17 |
| orf1ab,4577 | gct(A) | tct(S) | 0.011 | 18 |
| orf1ab,6054 | aat(N) | gat(D) | 0.011 | 11 |
| N , 376 | gct(A) | act(T) | 0.011 | 8 |
| N , 234 | atg(M) | atc(I) | 0.011 | 10 |
| orf3a , 172 | ggt(G) | cgt(R) | 0.009 | 12 |

| | | | | |
|---|---|---|---|---|
| orf3a , 38 | caa(Q) | cga(R) | 0.009 | 9 |
| orf8 , 92 | gaa(E) | aaa(K) | 0.007 | 9 |
| orf1ab,4241 | atg(M) | att(I) | 0.007 | 10 |
| N , 292 | atc(I) | acc(T) | 0.007 | 13 |
| orf6 , 33 | ata(I) | aca(T) | 0.006 | 12 |
| orf3a , 196 | gga(G) | gta(V) | 0.006 | 8 |
| S , 501 | aat(N) | tat(Y) | 0.005 | 8 |
| orf1ab,2606 | atg(M) | ata(I) | 0.005 | 9 |

**Table S3**

state 1 state 2 #simultaneous,#1Follows2,#2Follows1,#1alone,#2alone,Poisson Score,Binomial Score

| | | | | | | | | |
|---|---|---|---|---|---|---|---|---|
| orf1ab\|N6054D | orf3a\|G172V | 8 | 1 | 0 | 2 | 29 | 4.500297 | 54.71266 |
| N\|M234I | N\|A376T | 6 | 0 | 0 | 4 | 2 | 4.570825 | 45.70371 |
| orf3a\|G172R | orf3a\|V202L | 6 | 0 | 1 | 6 | 45 | 4.71351 | 35.39944 |
| orf3a\|Q38R | orf3a\|G172R | 5 | 1 | 0 | 3 | 7 | 4.716511 | 40.63884 |
| orf3a\|Q38R | orf3a\|V202L | 4 | 1 | 1 | 4 | 47 | 4.713664 | 27.3363 |
| orf1ab\|A4577S | orf1ab\|V5168L | 4 | 0 | 0 | 14 | 13 | 4.507175 | 20.22504 |
| orf6\|I33T | N\|I292T | 3 | 2 | 0 | 7 | 10 | 5.094976 | 28.08009 |
| orf3a\|Q57H | N\|A376T | 3 | 0 | 5 | 67 | 0 | 4.507881 | 15.89292 |
| orf1ab\|M3087I | N\|M234I | 3 | 0 | 0 | 29 | 7 | 4.576141 | 13.96507 |
| orf1ab\|V5168L | orf1ab\|E5585D | 3 | 0 | 1 | 14 | 41 | 4.508892 | 13.21509 |
| orf1ab\|A4577S | orf1ab\|E5585D | 3 | 0 | 1 | 15 | 41 | 4.510709 | 13.05832 |
| orf1ab\|M3087I | S\|S477N | 3 | 1 | 0 | 28 | 38 | 4.512101 | 11.51581 |
| orf1ab\|F3606L | orf3a\|G251V | 2 | 8 | 2 | 97 | 9 | 6.000492 | 21.155 |
| orf1ab\|V5168L | N\|A376T | 2 | 0 | 2 | 15 | 4 | 4.516232 | 17.86805 |
| orf1ab\|A4577S | N\|A376T | 2 | 0 | 2 | 16 | 4 | 4.515504 | 17.77572 |
| orf1ab\|V5168L | N\|M234I | 2 | 0 | 2 | 15 | 6 | 4.573518 | 16.88806 |
| orf1ab\|A4577S | N\|M234I | 2 | 0 | 2 | 16 | 6 | 4.572747 | 16.79526 |
| orf1ab\|E5585D | N\|A376T | 2 | 0 | 2 | 43 | 4 | 4.516768 | 15.89794 |
| S\|S477N | N\|A376T | 2 | 0 | 2 | 39 | 4 | 4.518391 | 15.71228 |
| orf1ab\|M3087I | N\|A376T | 2 | 0 | 1 | 30 | 5 | 4.518707 | 12.344 |
| orf1ab\|M3087I | orf1ab\|V5168L | 2 | 2 | 0 | 28 | 15 | 4.508153 | 11.72508 |
| orf1ab\|M3087I | orf1ab\|A4577S | 2 | 2 | 0 | 28 | 16 | 4.509245 | 11.63222 |
| orf3a\|Q57H | N\|M234I | 2 | 0 | 6 | 68 | 2 | 4.566414 | 11.12497 |
| orf1ab\|V5168L | S\|S477N | 2 | 1 | 1 | 14 | 38 | 4.51357 | 11.08201 |
| orf1ab\|E5585D | N\|M234I | 2 | 0 | 1 | 43 | 7 | 4.580214 | 11.05084 |
| S\|S477N | N\|M234I | 2 | 0 | 1 | 39 | 7 | 4.581944 | 10.9561 |
| orf1ab\|A4577S | S\|S477N | 2 | 1 | 1 | 15 | 38 | 4.514669 | 10.92172 |

| | | | | | | | | |
|---|---|---|---|---|---|---|---|---|
| orf1ab\|E5585D | S\|S477N | 2 | 1 | 1 | 42 | 38 | 4.514105 | 8.188651 |
| orf1ab\|M3087I | orf1ab\|E5585D | 2 | 1 | 0 | 29 | 43 | 4.51442 | 7.328134 |
| orf1ab\|M2606I | N\|D377Y | 1 | 1 | 7 | 7 | 140 | 6.263857 | 20.83678 |
| orf3a\|G172V | N\|D377Y | 1 | 0 | 12 | 36 | 135 | 6.238693 | 18.84826 |
| S\|L54F | N\|G204R | 1 | 46 | 0 | 48 | 9 | 6.73894 | 18.65395 |
| S\|D614G | S\|G769V | 1 | 0 | 84 | 37 | 3 | 6.021858 | 15.57247 |
| orf1ab\|V3718F | S\|D614G | 1 | 120 | 0 | 8 | 37 | 5.52613 | 14.76006 |
| orf1ab\|M3655I | orf3a\|K75N | 1 | 3 | 0 | 28 | 25 | 5.519532 | 11.20034 |
| N\|G204R | N\|D377Y | 1 | 0 | 54 | 9 | 93 | 6.29775 | 9.879498 |
| orf3a\|Q57H | N\|D377Y | 1 | 0 | 44 | 69 | 103 | 5.656636 | 6.994133 |
| S\|D614G | orf8\|R52I | 1 | 0 | 29 | 37 | 1 | 5.266064 | 6.870319 |
| orf1ab\|E4646D | orf1ab\|M6426I | 1 | 0 | 0 | 8 | 33 | 6.198232 | 6.284282 |
| S\|D1163Y | S\|G1167V | 1 | 0 | 0 | 53 | 13 | 6.341247 | 5.391188 |
| orf1ab\|I2873T | orf1ab\|L4653C | 0 | 4 | 9 | 16 | 30 | 6.259714 | 45.86258 |
| orf1ab\|K3353R | orf1ab\|R3353K | 0 | 2 | 9 | 224 | 0 | 5.610998 | 43.42658 |
| S\|M153T | N\|G204R | 0 | 35 | 0 | 10 | 10 | 5.958639 | 29.31147 |
| S\|M153T | N\|R203K | 0 | 35 | 0 | 10 | 29 | 5.958639 | 28.9565 |
| orf1ab\|Y4080H | N\|G204R | 0 | 22 | 0 | 3 | 10 | 5.938512 | 23.50665 |
| orf1ab\|Y4080H | N\|R203K | 0 | 22 | 0 | 3 | 29 | 5.933536 | 23.27143 |
| orf1ab\|K6958R | S\|D614G | 0 | 187 | 0 | 10 | 38 | 5.402686 | 22.38333 |
| orf8\|V62L | N\|G204R | 0 | 73 | 0 | 84 | 10 | 6.119245 | 19.05969 |
| orf1ab\|K3353R | N\|R203K | 0 | 97 | 0 | 129 | 29 | 5.892221 | 18.77014 |
| orf1ab\|K3353R | N\|G204R | 0 | 96 | 0 | 130 | 10 | 5.90064 | 18.56422 |
| orf8\|V62L | N\|R203K | 0 | 73 | 0 | 84 | 29 | 6.119245 | 18.55167 |
| S\|D614G | orf8\|V62L | 0 | 0 | 149 | 38 | 8 | 5.449372 | 18.05377 |
| S\|D614G | N\|D377Y | 0 | 0 | 141 | 38 | 7 | 5.034589 | 17.96663 |
| orf1ab\|F190L | orf3a\|Q57H | 0 | 12 | 0 | 1 | 70 | 6.03815 | 16.77026 |
| S\|D614G | S\|Q677H | 0 | 0 | 100 | 38 | 3 | 5.935846 | 16.30013 |
| S\|L54F | N\|R203K | 0 | 46 | 1 | 49 | 28 | 6.742405 | 16.20734 |

| | | | | | | | | |
|---|---|---|---|---|---|---|---|---|
| orf1ab\|N6054D | N\|D377Y | 0 | 0 | 12 | 11 | 136 | 6.245713 | 15.72337 |
| orf3a\|K75N | N\|G204R | 0 | 19 | 0 | 7 | 10 | 5.106553 | 14.77935 |
| S\|L54F | S\|D614G | 0 | 92 | 0 | 3 | 38 | 5.861692 | 14.62789 |
| orf3a\|K75N | N\|R203K | 0 | 19 | 0 | 7 | 29 | 5.105208 | 14.59149 |
| S\|D614G | S\|A1078S | 0 | 0 | 82 | 38 | 2 | 6.09119 | 14.40707 |
| S\|E583D | S\|D614G | 0 | 71 | 1 | 2 | 37 | 5.937666 | 14.33601 |
| orf1ab\|G3334S | N\|G204R | 0 | 17 | 0 | 6 | 10 | 5.263173 | 13.60533 |
| orf1ab\|G3334S | N\|R203K | 0 | 17 | 0 | 6 | 29 | 5.263173 | 13.43616 |
| orf1ab\|M2606I | orf1ab\|N6054D | 0 | 4 | 0 | 5 | 11 | 5.400463 | 13.152 |
| orf1ab\|M2606I | orf3a\|G172V | 0 | 4 | 0 | 5 | 37 | 5.408572 | 12.98435 |
| orf1ab\|G519S | N\|R203K | 0 | 55 | 0 | 70 | 29 | 6.321092 | 12.28335 |
| orf1ab\|G519S | N\|G204R | 0 | 54 | 0 | 71 | 10 | 6.323203 | 11.78333 |
| orf1ab\|R3353K | S\|N501Y | 0 | 3 | 0 | 6 | 8 | 5.660511 | 11.4252 |
| orf1ab\|R3353K | orf8\|R52I | 0 | 3 | 0 | 6 | 31 | 5.660511 | 11.36166 |
| orf1ab\|K6958R | N\|R203K | 0 | 77 | 0 | 120 | 29 | 6.282133 | 11.04242 |
| orf1ab\|K1202N | S\|D614G | 0 | 57 | 0 | 1 | 38 | 5.513131 | 11.01896 |
| orf1ab\|K6958R | N\|G204R | 0 | 76 | 0 | 121 | 10 | 6.291696 | 10.80214 |
| S\|G769V | N\|R203K | 0 | 40 | 0 | 48 | 29 | 6.67367 | 10.25443 |
| orf1ab\|A5620S | S\|D614G | 0 | 60 | 0 | 2 | 38 | 6.260371 | 9.741035 |
| S\|G769V | N\|G204R | 0 | 39 | 0 | 49 | 10 | 6.689564 | 9.610466 |
| S\|D614G | N\|M234I | 0 | 0 | 87 | 38 | 6 | 5.19501 | 9.320778 |
| orf1ab\|R3353K | orf1ab\|K5784R | 0 | 0 | 3 | 9 | 23 | 6.414769 | 9.035853 |
| orf1ab\|L4715P | S\|D614G | 0 | 64 | 0 | 3 | 38 | 6.133894 | 8.959117 |
| S\|D614G | orf3a\|G224C | 0 | 0 | 77 | 38 | 5 | 6.170657 | 8.735977 |
| S\|D614G | orf3a\|W131C | 0 | 0 | 90 | 38 | 7 | 6.142963 | 8.720943 |
| orf1ab\|K1655N | S\|D614G | 0 | 46 | 0 | 1 | 38 | 6.019098 | 8.592451 |
| orf3a\|Q57H | N\|M234I | 0 | 0 | 34 | 70 | 59 | 6.360114 | 8.447752 |
| orf1ab\|L4653C | orf3a\|Q57H | 0 | 18 | 0 | 21 | 70 | 6.545867 | 8.285261 |
| S\|D614G | S\|D1163Y | 0 | 0 | 52 | 38 | 2 | 5.833373 | 8.094074 |

| | | | | | | | | |
|---|---|---|---|---|---|---|---|---|
| orf1ab\|K5784R | S\|N501Y | 0 | 3 | 0 | 23 | 8 | 6.411709 | 8.057716 |
| orf1ab\|K5784R | orf8\|R52I | 0 | 3 | 0 | 23 | 31 | 6.411709 | 7.995575 |
| orf1ab\|M3655I | orf1ab\|M3712I | 0 | 0 | 3 | 32 | 9 | 5.803803 | 7.827436 |
| orf1ab\|M3934I | N\|G204R | 0 | 19 | 0 | 18 | 10 | 5.430497 | 7.536757 |
| S\|D80Y | S\|D614G | 0 | 56 | 0 | 3 | 38 | 6.053853 | 7.414205 |
| orf1ab\|M3934I | N\|R203K | 0 | 19 | 0 | 18 | 29 | 5.413549 | 7.386413 |
| S\|R21I | N\|G204R | 0 | 23 | 0 | 26 | 10 | 6.15872 | 7.189978 |
| S\|V1176F | N\|G204R | 0 | 24 | 0 | 28 | 10 | 5.6205 | 7.14312 |
| S\|R21I | S\|D614G | 0 | 47 | 0 | 2 | 38 | 5.963357 | 7.083728 |
| S\|R21I | N\|R203K | 0 | 23 | 0 | 26 | 29 | 6.15872 | 7.023278 |
| S\|V1176F | N\|R203K | 0 | 24 | 0 | 28 | 29 | 5.61286 | 6.972379 |
| orf1ab\|M5058I | S\|D614G | 0 | 29 | 0 | 0 | 38 | 6.018445 | 6.914705 |
| N\|R203K | N\|D377Y | 0 | 0 | 55 | 29 | 93 | 6.29775 | 6.904404 |
| N\|G204R | N\|I292T | 0 | 0 | 9 | 10 | 4 | 5.048052 | 6.892418 |
| orf1ab\|L4653C | S\|D614G | 0 | 38 | 0 | 1 | 38 | 5.802561 | 6.856622 |
| N\|R203K | N\|I292T | 0 | 0 | 9 | 29 | 4 | 5.048052 | 6.804806 |
| orf1ab\|G519S | S\|D614G | 0 | 112 | 0 | 13 | 38 | 5.70681 | 6.772864 |
| orf1ab\|R3353K | N\|G204R | 0 | 7 | 0 | 2 | 10 | 5.627283 | 6.591458 |
| orf1ab\|E4646D | N\|G204R | 0 | 7 | 0 | 2 | 10 | 6.16426 | 6.591458 |
| orf1ab\|E4646D | N\|R203K | 0 | 7 | 0 | 2 | 29 | 6.16426 | 6.51952 |
| orf1ab\|R3353K | N\|R203K | 0 | 7 | 0 | 2 | 29 | 5.627283 | 6.51952 |
| S\|M153T | S\|D614G | 0 | 43 | 0 | 2 | 38 | 5.882746 | 6.288292 |
| S\|E583D | N\|G204R | 0 | 30 | 0 | 43 | 10 | 6.588477 | 6.238237 |
| orf1ab\|K5784R | S\|D614G | 0 | 26 | 0 | 0 | 38 | 6.045193 | 6.199391 |
| S\|D614G | orf3a\|K75N | 0 | 0 | 26 | 38 | 0 | 5.072081 | 6.199391 |
| S\|D614G | S\|V1176F | 0 | 0 | 49 | 38 | 3 | 5.420556 | 6.10242 |
| S\|E583D | N\|R203K | 0 | 30 | 0 | 43 | 29 | 6.588477 | 6.053632 |
| orf1ab\|M3712I | orf3a\|K75N | 0 | 2 | 0 | 10 | 26 | 5.806855 | 5.990137 |
| orf1ab\|Y4080H | S\|D614G | 0 | 25 | 0 | 0 | 38 | 5.893439 | 5.960952 |

| | | | | | | | | |
|---|---|---|---|---|---|---|---|---|
| orf1ab\|Q2702H | orf3a\|Q57H | 0 | 12 | 0 | 15 | 70 | 5.166565 | 5.533389 |
| S\|N501Y | N\|G204R | 0 | 6 | 0 | 2 | 10 | 5.327998 | 5.448073 |
| orf3a\|Q57H | N\|R209I | 0 | 0 | 24 | 70 | 46 | 6.823591 | 5.402 |
| S\|N501Y | N\|R203K | 0 | 6 | 0 | 2 | 29 | 5.327998 | 5.387137 |
| orf1ab\|I300F | orf1ab\|E4646D | 0 | 0 | 2 | 50 | 7 | 6.191473 | 5.314163 |
| orf1ab\|A5048S | S\|D614G | 0 | 38 | 0 | 2 | 38 | 5.097618 | 5.31327 |

**Table S4**

List 1

| Variant_Mutation | Variant_ID |
|---|---|
| S\|N501Y | B.1.1.7,B.1.1.7_E484K,B.1.351,P.1,v2,vAfrica |
| S\|D614G | B.1.1.7,B.1.1.7_E484K,B.1.351,P.1,P.2 |
| S\|E484K | B.1.1.7_E484K,B.1.351,P.1,P.2,vAfrica |
| DELETION_ORF1A(NSP6)_3675-3677 | B.1.1.7,B.1.1.7_E484K,B.1.351,P.1 |
| S\|A570D | B.1.1.7,B.1.1.7_E484K,v2,v3 |
| N\|D3L | B.1.1.7,B.1.1.7_E484K,v2 |
| N\|S235F | B.1.1.7,B.1.1.7_E484K,v2 |
| S\|D1118H | B.1.1.7,B.1.1.7_E484K,v2 |
| S\|H69- | B.1.1.7,B.1.1.7_E484K,B.1.258_DELTA |
| S\|L18F | B.1.351,P.1,vAfrica |
| S\|P681H | B.1.1.7,B.1.1.7_E484K,v2 |
| S\|S982A | B.1.1.7,B.1.1.7_E484K,v2 |
| S\|T716I | B.1.1.7,B.1.1.7_E484K,v2 |
| S\|V70- | B.1.1.7,B.1.1.7_E484K,B.1.258_DELTA |
| N\|M234I | P.2,v1 |
| N\|T205I | B.1.351,vAfrica |
| ORF8\|Q27* | B.1.1.7,B.1.1.7_E484K |
| S\|A701V | B.1.351,vAfrica |
| S\|D215G | B.1.351,vAfrica |
| S\|D80A | B.1.351,vAfrica |
| S\|K417N | B.1.351,vAfrica |
| S\|Y144- | B.1.1.7,B.1.1.7_E484K |
| DELETION_S_242-245 | B.1.351 |
| E\|P71L | vAfrica |
| M\|R44S | vOceania |

| | |
|---|---|
| M\|T208I | vOceania |
| NSP12\|V720I | B.1.258_DELTA |
| NSP13\|A598S | B.1.258_DELTA |
| NSP9\|M101I | B.1.258_DELTA |
| N\|A119S | P.2 |
| N\|A376T | v1 |
| N\|A398V | v3 |
| N\|P67S | vOceania |
| N\|P80R | P.1 |
| N\|S206F | vOceania |
| ORF1A\|I4205V | B.1.429 |
| ORF1A\|L3458V | P.2 |
| ORF1A\|L3930F | P.2 |
| ORF1B\|D1183Y | B.1.429 |
| ORF3A\|G172V | vOceania |
| ORF3A\|S171L | vAfrica |
| ORF3A\|T223I | v3 |
| ORF8\|R52I | v2 |
| ORF8\|Y73C | v2 |
| S\|D138Y | P.1 |
| S\|H655Y | P.1 |
| S\|K417T | P.1 |
| S\|L452R | B.1.429 |
| S\|N439K | B.1.258_DELTA |
| S\|N501T | vOceania |
| S\|P26S | P.1 |
| S\|R190S | P.1 |
| S\|R246I | B.1.351 |
| S\|S13I | B.1.429 |

| | |
|---|---|
| S\|S477N | v1 |
| S\|T1027I | P.1 |
| S\|T20N | P.1 |
| S\|V1176F | P.2 |
| S\|W152C | B.1.429 |

List 2

| Variant_Mutation_1 | Variant_Mutation_2 | Variant_ID |
|---|---|---|
| S\|N501Y | S\|D614G | P.1,B.1.1.7,B.1.351,B.1.1.7_E484K |
| S\|N501Y | S\|E484K | P.1,B.1.351,B.1.1.7_E484K |
| S\|N501Y | S\|L18F | P.1,B.1.351 |
| S\|N501Y | S\|K417T | P.1 |
| S\|N501Y | S\|T20N | P.1 |
| S\|N501Y | S\|P26S | P.1 |
| S\|N501Y | S\|D138Y | P.1 |
| S\|N501Y | S\|R190S | P.1 |
| S\|N501Y | S\|H655Y | P.1 |
| S\|N501Y | S\|T1027I | P.1 |
| S\|N501Y | N\|P80R | P.1 |
| S\|N501Y | S\|H69- | B.1.1.7,B.1.1.7_E484K |
| S\|N501Y | S\|V70- | B.1.1.7,B.1.1.7_E484K |
| S\|N501Y | S\|Y144- | B.1.1.7,B.1.1.7_E484K |
| S\|N501Y | S\|A570D | B.1.1.7,B.1.1.7_E484K |
| S\|N501Y | S\|P681H | B.1.1.7,B.1.1.7_E484K |
| S\|N501Y | S\|T716I | B.1.1.7,B.1.1.7_E484K |
| S\|N501Y | S\|S982A | B.1.1.7,B.1.1.7_E484K |
| S\|N501Y | S\|D1118H | B.1.1.7,B.1.1.7_E484K |
| S\|N501Y | ORF8\|Q27* | B.1.1.7,B.1.1.7_E484K |
| S\|N501Y | N\|D3L | B.1.1.7,B.1.1.7_E484K |

| | | |
|---|---|---|
| S\|N501Y | N\|S235F | B.1.1.7,B.1.1.7_E484K |
| S\|N501Y | S\|K417N | B.1.351 |
| S\|N501Y | S\|D80A | B.1.351 |
| S\|N501Y | S\|D215G | B.1.351 |
| S\|N501Y | S\|R246I | B.1.351 |
| S\|N501Y | S\|A701V | B.1.351 |
| S\|N501Y | N\|T205I | B.1.351 |
| S\|N501Y | DELETION_S_242-245 | B.1.351 |
| S\|L452R | S\|S13I | B.1.429 |
| S\|L452R | S\|W152C | B.1.429 |
| S\|L452R | S\|L452R | B.1.429 |
| S\|E484K | S\|D614G | P.2 |
| S\|E484K | S\|V1176F | P.2 |
| S\|E484K | N\|A119S | P.2 |
| S\|E484K | N\|M234I | P.2 |
| S\|H69- | S\|N439K | B.1.258_DELTA |
| S\|V70- | S\|N439K | B.1.258_DELTA |

List 3

| Variant_Mutation_1 | Epistatically_Linked_Mutation | Variant_ID |
|---|---|---|
| N\|M234I | N\|A376T | P.2,v1 |
| S\|N501Y | N\|S235F | B.1.1.7,B.1.1.7_E484K,B.1.351,P.1,v2,vAfrica |
| S\|L18F | ORF7B\|S5L | B.1.351,P.1,vAfrica |
| S\|P681H | N\|G204R | B.1.1.7,B.1.1.7_E484K,v2 |
| S\|P681H | N\|R203K | B.1.1.7,B.1.1.7_E484K,v2 |
| ORF8\|Q27* | N\|G204R | B.1.1.7,B.1.1.7_E484K |
| S\|V1176F | N\|G204R | P.2 |
| ORF8\|Q27* | N\|R203K | B.1.1.7,B.1.1.7_E484K |
| S\|V1176F | N\|R203K | P.2 |

| | | |
|---|---|---|
| S\|L18F | ORF8\|A65V | B.1.351,P.1,vAfrica |
| S\|N501Y | N\|G204R | B.1.1.7,B.1.1.7_E484K,B.1.351,P.1,v2,vAfrica |
| S\|N501Y | N\|R203K | B.1.1.7,B.1.1.7_E484K,B.1.351,P.1,v2,vAfrica |
| N\|M234I | ORF3A\|Q57H | P.2,v1 |
| N\|M234I | S\|S477N | P.2,v1 |
| N\|T205I | ORF3A\|Q57H | B.1.351,vAfrica |
| N\|M234I | ORF7A\|T14I | P.2,v1 |
| N\|M234I | ORF3A\|Q57H | P.2,v1 |
| N\|M234I | ORF8\|S84L | P.2,v1 |

**Table S5**

| ORF | FirstNuc | SecondNuc | #Variants |
|---|---|---|---|
| ORF1ab | 12621 | 12747 | 1043 |
| ORF1ab | 13342 | 13460 | 1255 |
| ORF1ab | 14010 | 14136 | 1277 |
| ORF1ab | 15431 | 15530 | 1897 |
| ORF1ab | 18778 | 18909 | 8494 |
| E | 26269 | 26381 | 1640 |
| N | 28287 | 28358 | 3944 |
| N | 28681 | 28752 | 4026 |
| N | 28881 | 28979 | 61834 |
| N | 29125 | 29282 | 6332 |
